# An APEX2-based proximity-dependent biotinylation assay with temporal specificity to study protein interactions during autophagy in the yeast *Saccharomyces cerevisiae*

**DOI:** 10.1080/15548627.2024.2366749

**Published:** 2024-07-03

**Authors:** Yasmina Filali-Mouncef, Alexandre Leytens, Prado Vargas Duarte, Mattia Zampieri, Jörn Dengjel, Fulvio Reggiori

**Affiliations:** a Department of Biomedical Sciences of Cells and Systems, University of Groningen, University Medical Center Groningen, Groningen, AV, The Netherlands; b Department of Biology, University of Fribourg, Fribourg, Switzerland; c Department of Biomedicine, Aarhus University, Aarhus C, Denmark; d Department of Biomedicine, University of Basel, Basel, Switzerland; e Aarhus Institute of Advanced Studies (AIAS), Aarhus University, Aarhus C, Denmark

**Keywords:** Atg proteins, Atg8, Atg9, mass spectrometry, proteomics, proximity labeling

## Abstract

Autophagosome biogenesis is a complex process orchestrated by dynamic interactions between Atg (autophagy-related) proteins and characterized by the turnover of specific cargoes, which can differ over time and depending on how autophagy is stimulated. Proteomic analyses are central to uncover protein-protein interaction networks and when combined with proximity-dependent biotinylation or proximity labeling (PL) approaches, they also permit to detect transient and weak interactions. However, current PL procedures for yeast *Saccharomyces cerevisiae*, one of the leading models for the study of autophagy, do not allow to keep temporal specificity and thus identify interactions and cargoes at a precise time point upon autophagy induction. Here, we present a new ascorbate peroxidase 2 (APEX2)-based PL protocol adapted to yeast that preserves temporal specificity and allows uncovering neighbor proteins by either western blot or proteomics. As a proof of concept, we applied this new method to identify Atg8 and Atg9 interactors and detected known binding partners as well as potential uncharacterized ones in rich and nitrogen starvation conditions. Also, as a proof of concept, we confirmed the spatial proximity interaction between Atg8 and Faa1. We believe that this protocol will be a new important experimental tool for all those researchers studying the mechanism and roles of autophagy in yeast, but also other cellular pathways in this model organism.

**Abbreviations**: APEX2, ascorbate peroxidase 2, Atg, autophagy-related; BP, biotin phenol; Cvt, cytoplasm-to-vacuole targeting; ER, endoplasmic reticulum; LN2, liquid nitrogen; MS, mass spectrometry; PAS, phagophore assembly site; PL, proximity labeling; PE, phosphatidylethanolamine; PPINs, protein-protein interaction networks; PPIs, protein-protein interactions; RT, room temperature; SARs, selective autophagy receptors; WT, wild-type.

## Introduction

Macroautophagy, hereafter autophagy, is an intracellular catabolic process highly conserved among eukaryotes, which is involved in the turnover of cytoplasmic material, including unnecessary or aberrant protein complexes, dysfunctional or excess organelles and intracellular pathogens [1,2]. Upon autophagy induction, *or* cytoplasmic material targeted for destruction are sequestered by membranous cisterns called phagophores; these mature into autophagosomes that deliver their cargo into yeast and plant vacuoles or mammalian lysosomes, where they are degraded [1,2]. The resulting metabolites are recycled and used for either biosynthesis of new cellular components or energy production [1,2]. As a result, autophagy is key in preserving cellular homeostasis, sustaining cellular adaptations to stress and preventing cell aging, as well as being important in cell renewal, differentiation, and development [3,4]. Given its crucial physiological roles, it is not surprising that dysfunctional autophagy has been associated with several human diseases, including neurodegenerative disorders and cancer [3–6].

The hallmark of autophagy is the *de novo* biogenesis of autophagosomes [1,2]. Cargo sequestration is either nonselective and stimulated by cellular signals, or selective and triggered by the binding of the so-called selective autophagy receptors (SARs) to the specific cargoes [7]. The induction of autophagy leads to the nucleation of a small membranous cytoplasmic cisterna called the phagophore, which is formed at specific subcellular locations known as the phagophore assembly site (PAS) [8]. The phagophore then expands and closes forming an autophagosome [1,2]. Autophagosome biogenesis is orchestrated by the autophagy-related (Atg) proteins. About 20 of these proteins form the core Atg machinery, which is subdivided into 6 functional groups: the Atg1/ULK kinase complex, the phosphatidylinositol 3-kinase complex, the Atg9/ATG9A-positive vesicles, the Atg2-Atg18/ATG2 proteins-WDR45/WIPI4 complexes and two ubiquitin-like conjugation systems [1,2]. Using genetic screens, yeast *Saccharomyces cerevisiae* has been key in identifying and characterizing the *ATG* genes and their functional groups [9].

Upon autophagy induction, Golgi-derived vesicles carrying the transmembrane Atg9 homotrimers are recruited to the PAS, where they act as one of the membrane sources for phagophore nucleation but also as an organizational hub for the assembly of other components of the Atg machinery [1,2]. This recruitment is mediated via the interaction of Atg9 with either Atg17 during nonselective autophagy or Atg11 during selective autophagy, which allows Atg9-vesicles to interact with the Atg1 kinase complex pool at the PAS [10,11]. In yeast, Atg9 constitutively forms a complex with Atg23 and Atg27, which are also required for Atg9 transport from the peripheral sites to the PAS [12,13]. Atg9 localizes to the extremities of the expanding phagophore [14,15], where Atg2 is recruited by coincident binding to both Atg9 and phosphatidylinositol-3-phosphate [16]. The interaction with Atg9 is essential to also confine Atg2 to the extremities of the growing phagophore and for the subsequent association with Atg18 [16]. The Atg9-Atg2-Atg18 complex establishes membrane contact sites between the phagophore and the endoplasmic reticulum (ER), and it is central for the supply of lipids required for the phagophore to expand and close into a mature autophagosome [16]. Atg2 has lipid transfer activity *in vitro*, which is enhanced by Atg18 [17–21], while the lipid scramblase activity of Atg9 very likely distributes evenly the lipids delivered by the Atg2-Atg18 complex among the two leaflets of the phagophore membrane [17,22–24].

During autophagosome formation, the coordinated activity of the two ubiquitin-like conjugation systems leads to the covalent linkage of Atg8 with phosphatidylethanolamine (PE) present on both side of the phagophore membrane [25]. Atg8 is post-translationally processed by the Atg4 protease, which leads to the exposure of a C-terminal glycine residue [25]. Upon autophagosome formation induction, this glycine is conjugated to the amino group of PE through the concerted action of the E1-like activating enzyme Atg7, the E2-like conjugating enzyme Atg3, and the E3-like ligase complex Atg12–Atg5-Atg16 [25]. Once the autophagosome is completed, Atg4 recycles the Atg8 pool on its surface by deconjugating it from PE [25]. Atg8 participates in all steps of autophagy, i.e., phagophore nucleation and expansion as well as autophagosome maturation and fusion with vacuoles/lysosomes, by being a docking platform that recruits other Atg proteins and factors [25,26]. Atg8 also has intrinsic membrane tethering and fusogenic activities *in vitro*, which may be important for phagophore closure [27]. Finally, Atg8 has a central role in selective types of autophagy by mediating the sequestration of targeted cargoes into the autophagosome by either direct or indirect binding via SARs [7].

**Figure 1.**
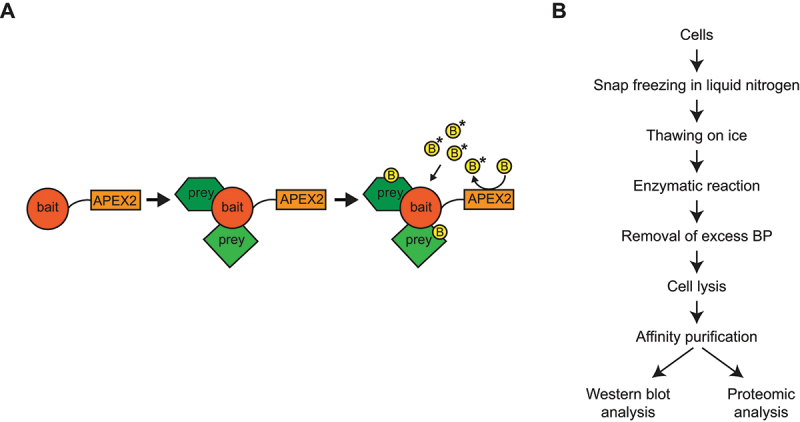
The novel APEX2-based PL assay with temporal specificity. (**A**) the principle of APEX2-mediated PL. The protein of interest or bait (e.g., Atg8 or Atg9) is fused to APEX2. Upon the addition of biotin-phenol (B) and H_2_O_2_ to the cells, APEX2 produces short-lived reactive biotin–phenoxyl molecules (B*) that react with proteins (or preys) adjacent to the bait. Biotin-labeled proteins can subsequently be isolated using streptavidin-conjugated beads and analyzed by either western blot or MS. (**B**) Stepwise overview of the novel APEX2-based PL procedure with temporal specificity.

Autophagy, like literally all cellular processes, is regulated and driven by a plethora of dynamic and often transient molecular interactions, which are also modulated by post-translational modifications such as phosphorylation by, e.g., the Atg1/ULK1 and MTORC1 kinase complexes [28]. Protein-protein interactions (PPIs) have historically been a main focus in biological research and the term interactome is often used interchangeably with protein-protein interaction networks (PPINs). The study of PPIs and PPINs is essential to understand the molecular function that proteins have within distinct cellular processes and ultimately elucidate their role in cell and organismal physiology [29]. Proximity-dependent biotinylation or proximity labeling (PL) coupled to protein mass spectrometry (MS) is an effective alternative to classical biochemical approaches such as immunoprecipitation or biochemical fractionation for proteomic analyses of PPINs [30,31]. In PL, a promiscuous labeling enzyme is fused to the protein of interest or bait. The addition of the substrate, i.e., biotin or biotin-phenol (BP) and H_2_O_2_, initiates the covalent transfer of biotin or its derivative, e.g., BP, to endogenous proteins within ca. 10 nm of the fusion protein [30,31]. Subsequently, the biotinylated proteins, or preys, are pulled down using streptavidin-conjugated beads and identified by MS [30,31]. PL-MS overcomes classical limitations of biochemical purification approaches since it bypasses the requirement to maintain PPIs intact during sample purification. This principle enables purification of the preys under harsh lysis and wash conditions because of the high affinity of the biotin-streptavidin interaction. Thus, transient and/or weak interactions can also be captured, and this is another main advantage of PL approaches [30,31]. Two enzymes commonly used for PL are APEX2 (Figure 1A), an engineered soybean ascorbate peroxidase, and TurboID, an engineered promiscuous mutant of the *Escherichia coli* biotin ligase BirA [30–32]. The advantage of APEX2 is its speed, i.e., proximal proteins can be tagged within 1 min upon the addition of BP and H_2_O_2_ [33]. However, the latter can be cytotoxic. In contrast, TurboID labeling is nontoxic and the addition of extra biotin initiates protein tagging, but on a timescale of tenths of min [34]. While PL procedures to identify PPIs by MS have become a standard approach in mammalian cells, technical difficulties still present major limitations for their wide application in yeast *S. cerevisiae*. TurboID requires relatively long labeling times and, consequently, it does not allow to examine PPIs at a precise time point, within short time windows [34–36]. APEX2, in contrast, requires the digestion or permeabilization of the cell wall [37–40], which leads to a change in the nutritional conditions and thus the experimental setup. As a result, the currently available PL procedures are not suitable for the study of the mechanism and cargoes of autophagy in changing nutrient conditions.

Here, we devised a rapid APEX2-based PL labeling protocol to examine neighbors of endogenous *S. cerevisiae* proteins by either western blot or MS, which preserves temporal and spatial specificity, and growth conditions. As a proof-of-principle, we analyzed the interactors of endogenous Atg8 and Atg9 in both rich and starvation conditions and detected numerous established binding partners of these two proteins. In addition, we validated the acyl-CoA synthetase Faa1, which is known to be involved in autophagy and to localize onto phagophore membranes [41], as a potential novel Atg8 interactor. Altogether, we have established a new methodology that allows mapping PPINs under different physiological conditions in *S. cerevisiae* cells.

## Results

### An APEX2-mediated PL procedure for temporal specificity

The APEX2-mediated PL procedures developed for mammalian cells (e.g., [33]) do not allow direct delivery of the co-substrate BP into living yeast because of the poor permeability of the cell wall and plasma membrane to this compound. Thus, yeast needs to be semi-permeabilized so that BP can enter cells. Two approaches have been developed to overcome this problem. The first relies on the digestion of the cell wall in a glucose-free, high-osmolarity solution [37,38], which has the disadvantage that it leads to metabolic changes, an osmotic shift, and a loss of temporal specificity as a consequence of incubating cells with the enzyme digesting the cell wall for 30–60 min. The second approach to semi-permeabilize yeast is to gradually freeze it in a glucose- and glycerol-containing buffer [39,40], but this approach also causes a loss of temporal specificity and/or to cellular alterations such as metabolic changes.

To overcome these problems, we developed a modified version of the methodology described previously [35,39], in which yeast is snap-frozen in liquid nitrogen (LN2) before subjecting semi-intact cells to APEX2-mediated PL. To test our novel procedure, we fused the N-terminus of Atg8 with the FLAG-APEX2 module and expressed it from the authentic *ATG8* promoter to avoid artifacts resulting from protein overexpression. The FLAG tag allows the detection of the fused protein using anti-FLAG antibodies. Western blot analysis using anti-FLAG antibodies confirmed the successful genomic insertion of DNA sequence encoding for FLAG-APEX2-Atg8, which has a molecular weight of approximately 45 kDa (Figure S1A). This chimera also complemented the maturation defect of the precursor Ape1 protease, the major cargo of the biosynthetic selective type of autophagy known as the cytoplasm-to-vacuole targeting (Cvt) pathway [42], of cells lacking *ATG8*, indicating that this fusion protein is functional (Figure S1A).

Next, we examined APEX2-mediated biotinylation by western blot by modifying the methodology described before as follows [35,39]: To preserve temporal specificity, we semi-permeabilized the equivalent of 25 OD_600_ of cells by snap freezing them in LN2 in a glycerol- and glucose-containing buffer, immediately upon collection, instead of gradually freezing them to − 80°C upon the washing of the harvested cells (Figure 1B). Subsequently, cells were thawed on ice instead of at room temperature (RT) to avoid changes in PPIs due to a reactivation of the cellular metabolism. Defrosted cells were briefly centrifuged at 16,200 *g* for 1 min at 4°C to discard the supernatant and quickly resuspended in the buffer used for snap freezing at RT to ensure APEX2-mediated enzymatic activity (Figure 1B). The PL reaction was rapidly performed by adding BP and H_2_O_2_. To simplify the protocol and make it faster, the excess BP and phenoxyl radicals were then removed by gently washing cells 5 times in an ice-cold quenching buffer by centrifugation at 16,200 *g* for 1 min at 4°C (Figure 1B), rather than filtering them 3 times at RT and subsequently washing them off from the filter before recovering the cells by centrifugation, as done previously [39]. Cell lysis was finally carried out by heating the cells at 55°C for 30 min under agitation in a lysis buffer and the presence of glass beads, with an interval every 10 min in which the samples were vortexed at RT for 1 min. Finally, the isolation of biotinylated proteins was performed using streptavidin-conjugated beads (Figure 1B).

**Figure 2.**
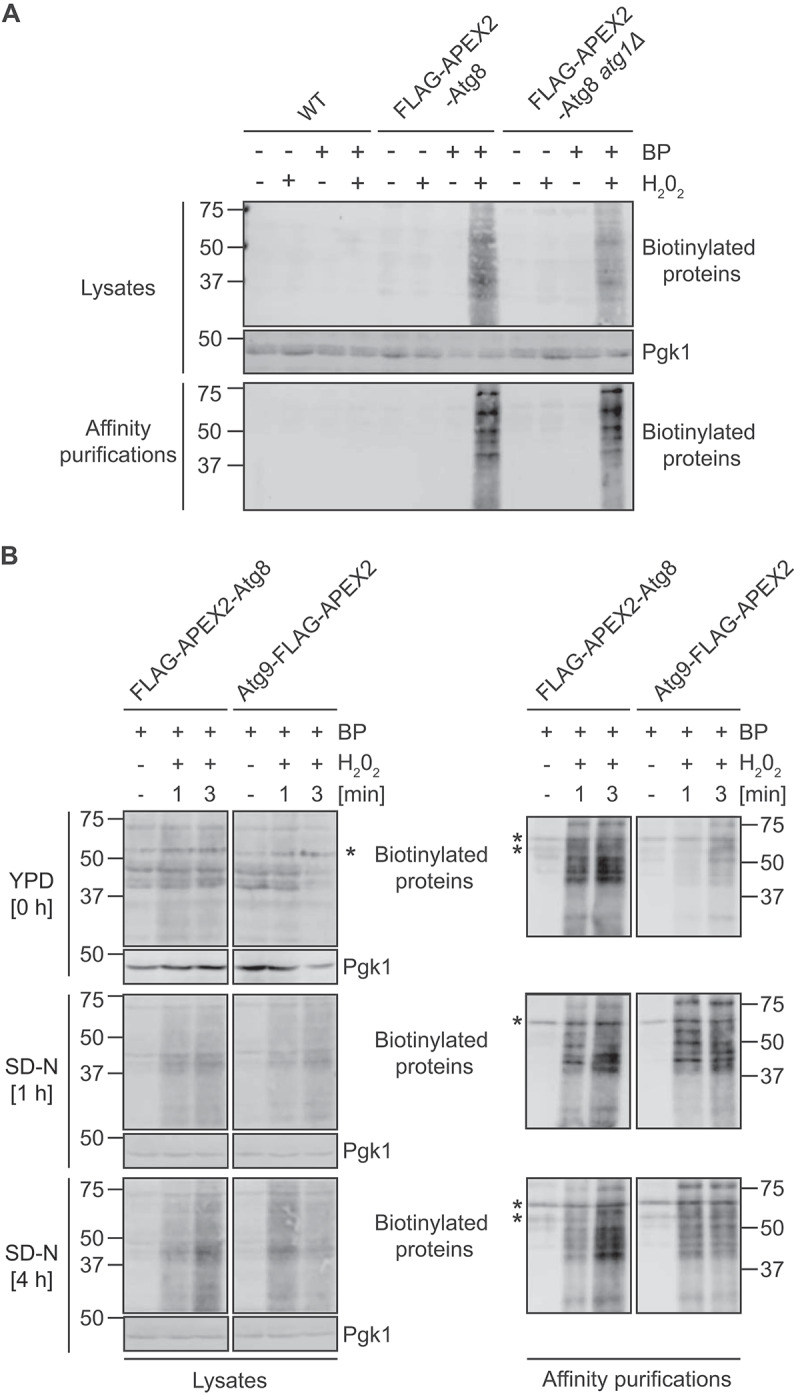
APEX2-mediated PL allows the tagging of neighboring proteins with temporal specificity under different physiological conditions. (A) Cells carrying endogenous FLAG-APEX2-NES-Atg8 and expressing (YFMLY141) or not Atg1 (YFMLY142, atg1∆) were grown overnight to a log phase in SMD medium containing 1% glucose. WT cells were also grown as a negative control. Cells were then nitrogen starved in SD-N medium for 1 h. The equivalent of 25 OD_600_ of cells were harvested and processed for APEX2-mediated PL for 3 min using BP and H_2_O_2_. Negative controls in which H_2_O_2_ and/or BP was omitted were also analyzed. Biotinylated proteins were then purified as described in Material and Methods. The lysates and the affinity-purified samples were resolved by SDS–PAGE before probing the western blot membranes with anti-biotin and anti-Pgk1 antibodies. Pgk1 served as a loading control. (B) Cells expressing FLAG-APEX2-NES-Atg8 (YFMLY141) or Atg9-FLAG-APEX2 (YFMLY170) were grown overnight to a log phase in SMD medium containing 1% glucose before being nitrogen starved in SD-N medium for 4 h. The equivalent of 25 OD_600_ of cells were harvested by centrifugation after 0, 1 and 4 h of nitrogen starvation. Cells were processed for APEX2-mediated PL by adding BP and H_2_O_2_ for 1 or 3 min and biotinylated proteins were purified as described in Materials and Methods. Both lysates and affinity purified samples were resolved by SDS–PAGE before probing the western blot membranes with anti-biotin and anti-Pgk1 antibodies. Pgk1 served as a loading control. Asterisks indicate unspecific bands also present in the biotin-containing loading buffer.

Initially, as a proof-of-principle, wild-type (WT) and *atg1Δ* cells expressing FLAG-APEX2-NES-Atg8 were grown to log phase (OD_600_ 0.8–1.2) and then nitrogen starved for 1 h before semi-permeabilization and PL by addition of BP and H_2_O_2_ for 3 min. WT cells expressing no FLAG-APEX2-NES-tagged construct were used as a negative control and processed in parallel. Western blot analysis of the whole cell extracts and the samples affinity purified with streptavidin-coated beads using anti-biotin antibodies revealed the specific appearance of biotinylated proteins upon addition of both BP and H_2_O_2_, and only in cells expressing FLAG-APEX2-NES-Atg8 (Figure 2A).

Then, we compared the biotinylation patterns obtained after different incubation times, i.e., 1 and 3 min, in different nutritional conditions, employing two different APEX2 tagged proteins, i.e., Atg8 and Atg9. Western blot analysis using anti-Ape1 antibodies confirmed endogenous tagging of Atg9-FLAG-APEX2 and the functionality of the resulting fusion since Ape1 was processed normally (Figure S1B); dysfunctional Atg9 causes complete Cvt pathway block [43]. Cells expressing FLAG-APEX2-Atg8 or Atg9-FLAG-APEX2 were grown to log phase and then nitrogen starved for 4 h. Cells were collected in growing conditions and immediately snap frozen after both 1 and 4 h of nitrogen starvation. Cells were then processed for PL as described above. Western blot analysis of affinity-purified samples using streptavidin-coated beads revealed the specific appearance of biotinylated proteins upon the addition of both BP and H_2_O_2_ (Figure 2B). These data also showed that 1 min reaction time is sufficient to detect biotinylated proteins under the different tested nutritional conditions for the two different membrane-associated fusion proteins and that after 3 min increased levels of biotinylated proteins were detectable.

Altogether, these results show that the developed method allows APEX2-mediated PL of proteins under different physiological conditions and temporal resolutions.

#### APEX2-mediated PL followed by MS allows the detection of specific neighbor proteins

**Figure 3.**
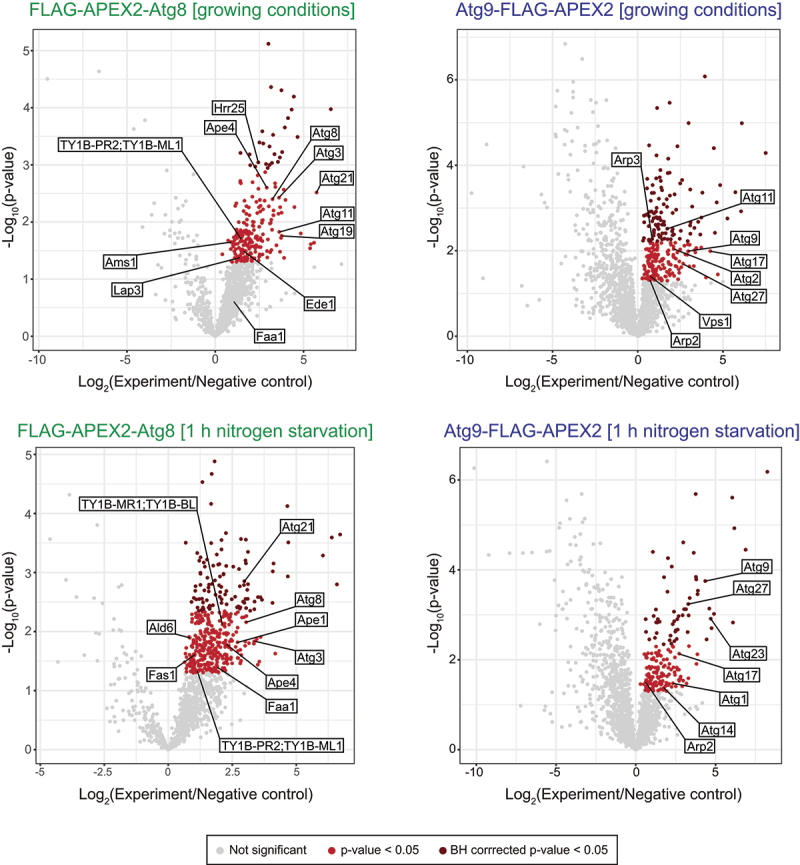
MS-based proteomic analyses of the proximity labeling experiments. Volcano plots showing normalized log_2_-transformed protein abundances of FLAG-APEX2-NES-Atg8 (YFMLY141) and Atg9-FLAG-APEX2-NES (YFMLY170) compared to their respective negative control in which H_2_O_2_ was not added at both growing conditions and 1 h nitrogen starvation. Dark and light red dots represent significantly enriched proteins by q-value <0.05 or p-value <0.05, respectively, in APEX2-Atg8 and Atg9-APEX2 cells compared to their respective negative control. Grey dots represent proteins that were not significantly enriched. Values from at least 3 independent experiments were used to determine statistical significance, which was evaluated using the two-tailed t-test to calculate the p-values and the Benjamini-Hochberg method to obtain the q-values. Highlighted proteins correspond to known interactors of Atg8 or Atg9, which were successfully enriched using the new APEX2-based PL procedure coupled to MS.

To test if we could identify known neighbor proteins of Atg8 or Atg9 using our procedure, we coupled APEX2-mediated PL to MS. For this, we employed both strains expressing FLAG-APEX2-Atg8 or Atg9-FLAG-APEX2 as a bait to be able to evaluate the labeling specificity. The procedure was scaled up to have sufficient material for the proteomic analysis, i.e., the equivalent of 100 OD_600_ of cells were used for the PL labeling (Figure 1B). Moreover, three aliquots of the equivalent of 100 OD_600_ of cells were collected per experiment. The PL labeling was carried out in two of these three samples while the third was kept untreated and used as the background for the proteomic analysis. Of the two samples in which the biotinylation reaction was performed, one was used to verify that the PL labeling worked using the above protocol coupled to western blot analysis before using the second one for the proteomic analysis. For this proof-of-principle experiment, we decided to analyze cells grown in rich medium or nitrogen starved for 1 h to induce autophagy (*n*=four biological replicates). The biotinylated proteomes were affinity purified using streptavidin-coated beads upon cell lysis and analyzed by MS (Figure 1B). Values from at least 3 repeats were used to identify those proteins that were significantly enriched (p < 0.05) in comparison to the control. Hits were also subjected to multiple testing correction using the Benjamini-Hochberg method (FDR <0.05; Figure 3 and Table S1).

**Figure 4.**
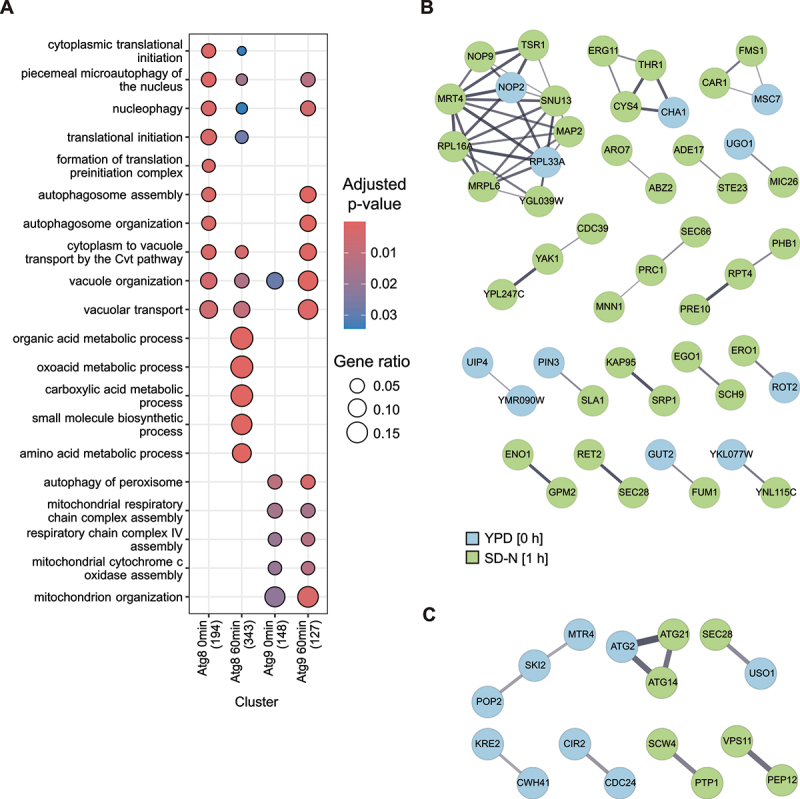
GO enrichment and PPI network. (A) Proteins found in the PL experiments with a 2-fold or higher enrichment and a p-value <0.05 were used to perform GO Biological Processes enrichment analysis. The numbers between brackets indicate the number of proteins in a particular cluster. (B-C) Time-specific protein-protein interaction networks. PPINs from the STRING database (confidence cutoff 0.4) were retrieved for proteins being enriched in only one of the two timepoints for the FLAG-APEX2-Atg8 (B) and Atg9-FLAG-APEX2 PL experiments (C). Edge widths are proportional to the StringDB interaction score. Non-connected proteins are not shown.

With our procedure, we identified 219 and 403 Atg8 neighbor proteins in rich and nitrogen starvation conditions, respectively, and 255 and 180 proteins in the proximity of Atg9 in rich and nitrogen starvation conditions, respectively (Figures S2A and S2B). Some of the hits were detected in the 3 or 4 of the samples and they may represent background labeling of APEX2 (Figures S2C and S2D). Since both Atg8 and Atg9 are present on all autophagosomal intermediates, however, these two proteins may share some common neighbor proteins. Atg2 is one of these cases (Tables S2-S5). Nonetheless, our proteomic analyses identified an important number of proteins that are specifically proximal to Atg8 or Atg9, which in several instances were condition-specific (Figure S2D). Detailed examination of all the hits revealed that numerous of them are known Atg8 or Atg9 interactors, and/or have been associated to autophagy in yeast (Tables S2-S5). For example, we detected as an Atg8 interactors Atg3, the E2-like conjugating enzyme that links Atg8 to PE [25]. We have also identified different SARs as Atg8 binding partners, such as the ones involved in the Cvt pathway (Atg19 [44]) and selective turnover of the aberrant clathrin-mediated endocytosis/CME protein condensates by autophagy (Ede1 [45]) (Figure 3, Tables S2 and S3). Consistently, all the known cargo proteins of the Cvt pathway (Ape1, Ape4, Ams1, Lap3 and the Ty retrotransposon) [42], as well as several components of the aberrant clathrin-mediated endocytosis condensates (Chc1, End3, Sla1 and Syp1) [45], were also found in the APEX2-Atg8 samples (Tables S2 and S3). We also detected three of the cargoes, Ald6, Fas1, and Pfk2, that are selectively turned over during bulk autophagy as Atg8 neighbor proteins [46–48] (Figure 3, Tables S2 and S3). In the case of Atg9, we identified several proteins, Atg1, Atg2, Atg14, Atg23 and Atg27 [12,13,16,49], which are known to directly or indirectly interact with Atg9, but also factors such as Atg11, Atg17, the Arp2-Arp3 complex and Vps1 that play a role in Atg9 trafficking [10,11,50,51] (Figure 3, Tables S4 and S5). Finally, several of the potentially new and uncharacterized Atg8 and Atg9 neighbor proteins have been detected in other autophagy-related high-throughput proteomic studies (Tables S2-S5). GO *Biological Processes* enrichment analysis of the detected proteins showed that we enriched proteins involved in selective and bulk autophagy, underscoring the quality of the data and the strength of the approach (Figure 4A). Moreover, this and a STRING analysis (Figures 4B,C) highlighted different time- and condition-specific PPIs for Atg8 and Atg9, which are consistent with the different roles that these two proteins have in autophagy. Altogether, these data prove the validity of our APEX2-based PL procedure and its sensitivity in examining the process of autophagy in yeast.

**Figure 5.**
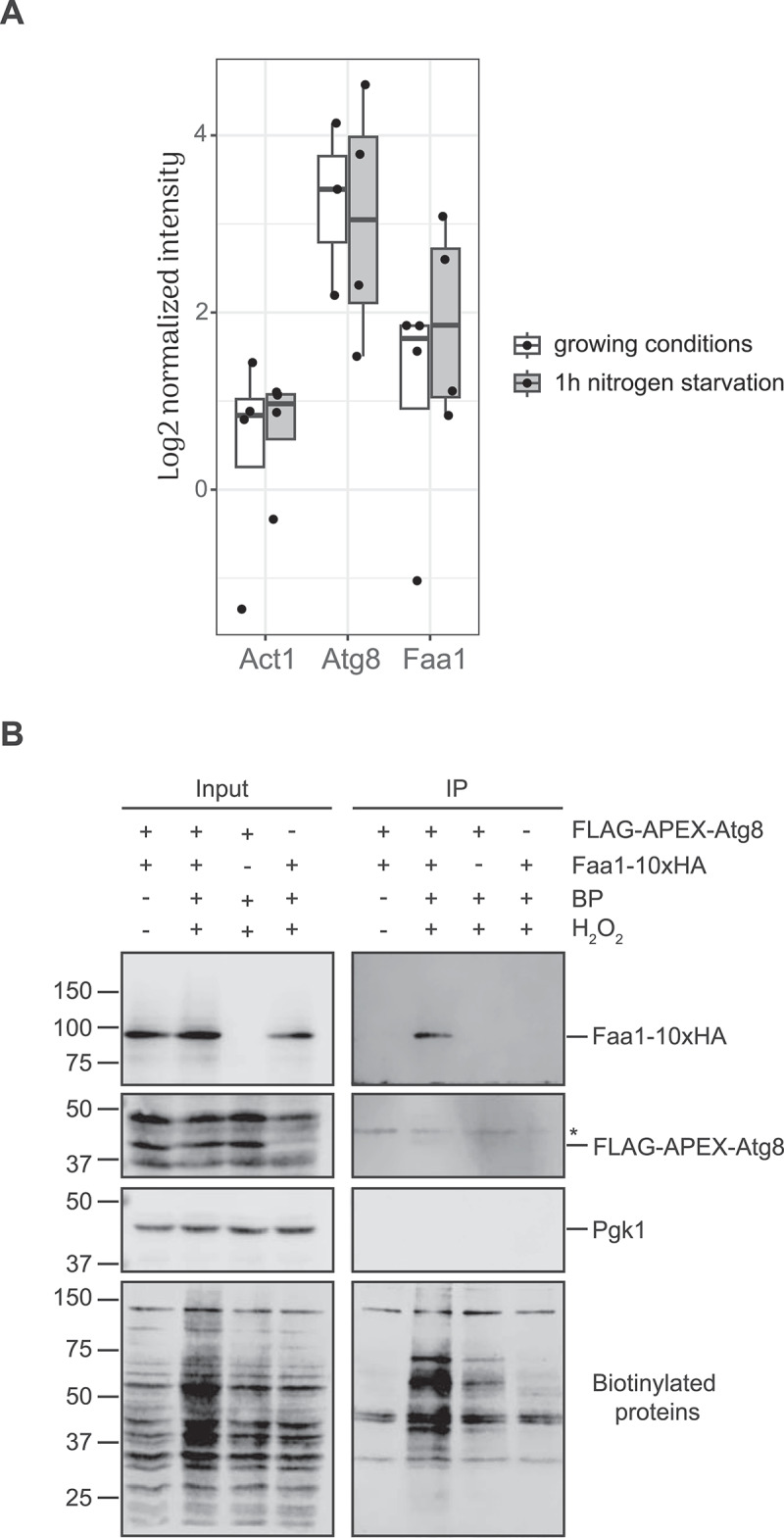
Faa1 displays proximity to Atg8 under autophagy-inducing conditions. (A) Boxplot visualization showing the enrichments for Atg8, Faa1 and Act1. Intensities from the PL samples were normalized to their controls. Act1 was chosen as an example for a protein that is not enriched. Boxes represent median values and 25^th^ and 75^th^ percentiles. (B) SEY6210 strains expressing individually endogenous FLAG-APEX2-NES-Atg8 and Faa1-10×HA (YFMLY080 and PVY023, respectively) or in combination (PVY029), were grown overnight to log phase in YPD medium and then nitrogen starved in SD-N medium for 1 h. The equivalent of 25 OD_600_ of cells were collected by centrifugation and processed for APEX2-mediated PL as in Figure 2. The PL was conducted for 1 min and as a negative control, the PVY029 strain was also processed as the rest of the samples but without adding H_2_O_2_ and BP during the PL reaction. The lysates and the affinity purified samples were finally resolved by SDS – PAGE before probing the western blot membranes with anti-biotin, anti-HA (to detect the Faa1-10×HA fusion), anti-Atg8 (to detect the FLAG-APEX2-Atg8 chimera) and anti-Pgk1 antibodies. Pgk1 served as a loading control. The asterisk indicates a protein present in eluates that it is recognized by the anti-Atg8 antiserum. The experiment was repeated 5 times, and one repeat is shown in the figure.

To validate our findings and underline the potential of our procedure in identifying new proteins either involved in yeast autophagy or being autophagosomal cargoes, we decided to focus on the proximity between Atg8 and Faa1 detected upon autophagy induction by nitrogen starvation (Figures 3 and 5A, Table S3). Faa1 is an acyl-CoA synthetase that redistributes to the forming autophagosome, and it is important to locally activate the fatty acids required for the *de novo* phospholipid biosynthesis and phagophore expansion [41]. However, it remains to be established how this enzyme is recruited onto autophagosomal membranes. To validate Faa1 as a neighbor protein of Atg8, Faa1 was endogenously fused with the 10×HA tag in a strain expressing FLAG-APEX2-Atg8 under the control of the authentic promoter. The functionality of the Faa1-10×HA fusion was assessed by creating the same chimera in a strain carrying the Pho8∆60 reporter [52], which allows to measure bulk autophagy (Figure S3). We then performed the small-scale APEX2-based PL (Figure 2) in cells nitrogen-starved for 1 h. Biotinylated proteins were isolated with streptavidin-conjugated beads and upon separation by SDS-PAGE, the western bot membrane was probed with anti-biotin, anti-HA, and anti-Pgk1 antibodies. This analysis specifically detected Faa1-10×HA in the sample in which H_2_O_2_ and BP were added and not in the one in which these two compounds were omitted (Figure 5B). Moreover, this fusion protein was not found in the samples from cells only expressing FLAG-APEX2-Atg8 or Faa1-10×HA (Figure 5B). This result revealed that Faa1 is a protein proximal to Atg8 and confirmed the suitability of the developed proteomic approach in identifying established and novel Atg protein interactors.

Taken together, these results show that the developed method allows the detection of neighbor proteins with temporal specificity under different physiological conditions.

## Discussion

In this study, we present an improved protocol for APEX2-mediated PL followed by western blot or MS to map PPINs in budding yeast *S. cerevisiae*. Our protocol preserves temporal specificity, which is crucial to study PPINs between Atg proteins and to identify autophagosomal cargos under specific autophagy inducing conditions and at determined time points upon triggering this degradative pathway. In addition to the preservation of temporal specificity, our procedure is simpler and faster in comparison to previously described APEX2-mediated PL approaches [37–40,53,54]. Two detailed step-by-step protocols are presented in Information S1 and S2.

The temporal specificity is preserved within a few minutes of time scale. This means that the assay is carried out so that the interactors at a particular time point in an experiment in which conditions change over time, remain the same. That is, it practically allows to biotinylate the interactors at the precise chosen time points of the experiments. Previous protocols involve steps at RT and/or change of growing conditions with living cells after collection [37–40,53,54], which can lead to a change of the initial experimental condition and ultimately the microenvironment of the bait, i.e., the observed PPINs. The temporal specificity has been achieved by immediately snap-freezing collected cells in LN2. Moreover, cells are thawed on ice, and they are incubated at RT for the APEX2-mediated PL reaction for only 1 min. However, the PL time depends on the abundance of the tagged protein and a pilot experiment similar to the one carried out with FLAG-APEX2-Atg8 and Atg9-FLAG-APEX2 (Figure 2B), has to be initially performed to estimate which PL time is optimal. One possible drawback of our approach could be that semi-permeabilization by snap freezing might destroy cellular integrity and thus alter subcellular proximities. However, this seems not the case in our experiments, since we identified known interactors of both Atg8 and Atg9 using our procedure (Figure 3 and Tables S2-S5). An additional advantage of our PL labeling protocol is that it is also simpler and faster than what has been previously published [37,39,40,53,54], which also helps in avoiding cellular changes and thus contributes in maintaining temporal specificity.

More recently, an alternative and clickable APEX2 substrate, Alk-Ph, which is water soluble and therefore cell wall permeable, has been developed [53,54]. However, this probe, which is not commercially available yet, has to be pre-incubated for 30 min at 25°C in PBS before adding H_2_O_2_ to initiate the PL labeling [53,54], leading to a loss of the temporal specificity. Additionally, APEX2 fusion proteins have to be overexpressed to obtain sufficient labeling [54] and overexpression has the disadvantage that it can cause experimental artifacts.

Despite their low abundance, our APEX2-based PL procedure has allowed identifying the interacting partners of endogenous Atg8 and Atg9 when coupled to MS (Figure 3). Thus, contrary to some of the other available approaches [37,53,54], our protocol permits to study cellular processes avoiding possible artifacts resulting from the overexpression of fusion proteins. We were able to map known PPINs not only when the expression of Atg8 and Atg9 is upregulated, i.e., upon autophagy induction [55], but also during growing conditions (Figure 3). We found for instance SARs, established and potential autophagosomal cargoes as Atg8 neighbor proteins, and proteins that are in complex with and are involved in the trafficking of Atg9 (Tables S2-S5). However, we did not detect all the expected binding partners. For example, we did not find in our datasets Atg7 and the components of the Atg12–Atg5-Atg16 complex, which are involved in the conjugation of Atg8 to PE [25]. Atg18, which together with Atg9 and Atg2 establishes membrane contact sites between the phagophore and the ER [16], was also not uncovered as an Atg9 neighbor. One plausible explanation is that the positioning of the APEX2 fused with either Atg8 or Atg9 outdistance the tyrosines present on these proteins impeding their biotinylation. Another not mutually exclusive explanation is that the amounts of these proteins make them more difficult to be detected by MS. This notion is supported by the observation that two Atg proteins, i.e. Atg14 and Atg21, were only detected as interactors of Atg9 in the sample from cells starved for nitrogen, a condition that increases the expression of the Atg proteins [55]. A strategy to bypass these putative difficulties could be to fuse APEX2 with the other terminus of the bait if this still allows having functional chimeras, something possible with Atg9 but not Atg8, and/or to scale up the amount of OD_600_ equivalents of cells used for the proteomic analysis.

We found the acyl-CoA synthetase Faa1 among the neighbor proteins of Atg8 under nitrogen starvation conditions (Table S3), and we confirmed this proximity by APEX2-based pull-down (Figure 5). As Atg8, Faa1 is distributed over the surface of the expanding phagophore [41] and consequently, this result is not totally unexpected. How Faa1 is associated with phagophore membranes is unknown and our findings point to Atg8 possibly being involved in Faa1 recruitment. Future studies are necessary to determine whether Atg8 directly or indirectly binds Faa1, and how. However, it cannot be excluded that those two proteins are simply proximal without interacting. Because PL cannot distinguish between an interaction and proximity, potential direct or indirect binding partners must be verified with immunoprecipitation experiments. The latter, however, are not optimal to detect transient and/or weak interactions, and, consequently, other approaches such as bimolecular fluorescence complementation or *in vitro* binding assays may also be considered.

The development of a catalytically efficient APEX2 peroxidase has permitted to increase the sensitivity of PL and since, this enzyme has been widely used for proteomic analyses in mammalian cells, also for the analysis of autophagy relevant PPINs [32]. However, examples of applications of not only APEX2- but also TurboID-mediated PL in yeast are rare because of the lengthy labeling and processing time, and the strongly limited temporal specificity. Our procedure overcomes the current limitations and therefore it will help to expand the protocol toolkit for detection of PPIs by MS in this model organism. Importantly, our protocols open the opportunity for kinetic studies aimed at understanding the changes in interactions between proteins within a few minutes of resolution and consequently are well suited for studies on autophagy.

## Materials and methods

### Plasmids

To generate the pFA6a-FLAG-APEX2-NES-NatMX6 vector, the DNA sequence encoding *FLAG-APEX2-NES* was generated by PCR using the pDP287 (pRS415-FLAG-APEX2-NES-APE1, a kind gift from Claudine Kraft) plasmid as the template and cloned as a PacI-AscI fragment into pFA6a-GFP(S65T)-NatMX6 plasmid [56] digested with the same restriction enzymes. The nuclear export sequence/NES is necessary to allow APEX2 fusions to localize to the cytoplasm.

The pYFML034 (pRS404 1000bp_KanMX6_ATG8prom-FLAG-APEX2-NES-ATG8_1000bp) plasmid was created by sequentially cloning a DNA fragment encoding a 1000 bp homology region upstream of the *ATG8* promoter as a ApaI-ApaI fragment, the drug resistance gene *KanMX6* as a ApaI-XhoI fragment, the endogenous *ATG8* promoter as a XhoI-ClaI fragment, the *FLAG-APEX2* tag as a ClaI-NotI fragment and the *ATG8* gene followed by a 1000 bp homology region downstream of *ATG8* as a NotI-SacII fragment in the pRS404 vector [57]. The (GGGGS)_4_ linker was inserted in front of *ATG8* with an appropriate primer when this gene and the 1000 bp downstream were amplified by PCR from the *S. cerevisiae* genomic DNA. The *S. cerevisiae* genomic DNA was also used as the template to amplify by PCR both the 1000 bp homology region upstream of the *ATG8* promoter and the *ATG8*. The pDP287 vector was used as the template for the amplification of the *FLAG-APEX2-NES* tag.

The integrative pYFML018 vector was created by cloning in succession the *ATG8* promoter (*ATG8prom*), the *FLAG-APEX2-NES* coding sequence, and the *ATG8* gene as XhoI-ClaI, ClaI-NotI, and NotI-SacII fragments, respectively, in the pRS403 vector [57]. The primer used to amplify *ATG8* introduced the (GA)_5_ amino acid linker at the N-terminus of Atg8. *G*enomic DNA was used to amplify the *ATG8* promoter and gene whereas the pDP287 vector was used as the template for the amplification of the *FLAG-APEX2-NES* tag.

The pFA6a-10×HA-KanMX6 and pFA6a-10×HA-TRP1 vectors were created by excising *GFP-ADH1* terminator (*ADH1ter*) with PacI and BglII from the pFA6a-GFP-KanMX6 and pFA6a-GFP-TRP1 plasmids [58] and replacing it with the *10×HA-ADH1ter* fragment generated by PCR from the genomic DNA from a strain expressing the *ERD2-10×HA* fusion followed by *ADH1ter* (a kind gift of Manfred Schmidt) and digested with PacI-BglII as well.

### Strains and growth

The *S. cerevisiae* strains used in this study are listed in Table 1. The *ATG8* gene was knocked out in the YSBN5 strain by replacing the coding region with the antibiotic resistance *hphNT1* gene using PCR primers containing 60 bases of identity to the regions flanking the open reading frame, and generating the YFMLY099 strain [58]. Chromosomal tagging of the *ATG9* gene at the 3′ end with *FLAG-APEX2-NES* was performed using PCR-based integration by homologous recombination of the sequence encoding for the tag using the pFA6a-FLAG-APEX2-NES-NatMX6 plasmid as the template. Chromosomal tagging of *ATG8* at the 5’ end was carried out by replacing the *hphNT1* cassette used to knock out the *ATG8* gene with the *KanMX6-ATG8pr-FLAG-APEX2-(GGGGS)*_*4*_*-ATG8* fragment by homologous recombination of 1000 bp of identity upstream the *ATG8* promoter and downstream the *ATG8* gene. This fragment was amplified by PCR using pYFML034 as a template. Gene knockouts and modifications were verified by western blot using specific antibodies and/or PCR analysis.

**Table 1.** Strains used in this study.

Name	Genotype	Origin
PVY023	SEY6210 *FAA1-10×HA:KanMX6*	This study
PVY029	SEY6210 *ATG8prom-FLAG-APEX2-NES-ATG8:HIS3 atg8Δ::LEU2 FAA1-10×HA:KanMX6*	This study
PVY048	SEY6210 *PHO8Δ60 pho13Δ::HIS3 FAA1-10×HA:TRP1*	This study
RGY352	SEY6210 *PHO8Δ60 pho13Δ::HIS3 atg1Δ::hphNT1*	This study
SAY086	SEY6210 *atg8Δ::LEU2*	This study
SEY6210	*MATa ura3–52 leu2–3,112 his3-∆200 trp1-∆901 lys2–801 suc2-∆9 mel GAL*	[59]
YFMLY080	SEY6210 *ATG8prom-FLAG-APEX2-NES-ATG8:HIS3 atg8Δ::LEU2*	This study
YFMLY099	YSBN5 *atg8Δ::hphNT1*	This study
YFMLY141	YSBN5 *atg8Δ::hphNT1 hphNT1:KanMX6-FLAG-APEX2-NES-ATG8*	This study
YFMLY142	YSBN5 *atg8Δ::hphNT1 atg1Δ::NatMX6 hphNT1:KanMX6-FLAG-APEX2-NES-ATG8*	This study
YFMLY170	YSBN5 *ATG9-FLAG-APEX2-NES:NatMX6*	This study
YSBN5	*MATa HO:loxP-TEF1p-ble-TEF1t-loxP ura3–52:URA3*	Euroscarf [60]
WLY176	SEY6210 *PHO8∆60 pho13∆:HIS3*	[61]

To delete *ATG8* in the SEY6210 strain, the coding region was replaced with the *K. lactis LEU2* gene flanked by *loxP* sites *(loxP-kanMX-loxP)* amplified by PCR using the pUG73 vector as a template and primers containing 60 bases of identity to the regions flanking the open reading frame [62]. The knockout of *ATG8* was verified by western blot using an anti-Atg8 antiserum [63]. PCR-based integration of the 10×HA tag at the 3’ end of *FAA1* was used to generate strains expressing C-terminal Faa1-10×HA fusion protein under the control of the authentic promoter [58]. The plasmid template for integration were pFA6a-10×HA-KanMX6 and pFA6a-10×HA-TRP1. The *ATG8prom-FLAG-APEX2-NES-(GA)*_*5*_*-ATG8* construct was integrated in the yeast genome of the *atg8∆* strain SAY086 upon linearization of the pYFML018 integrative plasmid with a single restriction site in the selectable marker and subsequent transformation with the linear DNA. Gene knockouts and modifications were verified by western blot using specific antibodies and/or PCR analysis.

Yeast was grown in rich medium (YPD; 1% yeast extract [Formedium, YEA03], 2% peptone [Formedium, PEP03], 2% glucose) or synthetic minimal medium (biotin-free SMD; 1% glucose, 0.69% yeast nitrogen base without amino acids and biotin (Formedium, CYN0310), and supplemented with amino acids and vitamins except for biotin) at 30°C and starved in synthetic minimal medium lacking nitrogen (biotin-free SD-N; 1% glucose, 0.19% yeast nitrogen base without amino acids, ammonium sulfate and biotin [Formedium, CYN3210]) also at 30°C.

Yeast was grown either in rich (YPD; 1% yeast extract, 2% peptone, and 2% glucose) or synthetic minimal medium (SMD; 0.69% yeast nitrogen base without amino acids, 1% glucose and supplemented with amino acids and vitamins except biotin) at 30°C. Starvation experiments were conducted in synthetic minimal medium lacking nitrogen (SD-N; 0.19% yeast nitrogen base without amino acids, and ammonium sulfate, and 2% glucose or 0.19% yeast nitrogen base without amino acids, ammonium sulfate and biotin, and 1% glucose) at 30°C.

### Western blot analyses

For western blot analyses, the equivalent of 5 OD_600_ of cells were collected by centrifugation at 13,000 *g* for 1 min, resuspended in 500 μl of ice-cold 10% trichloroacetic acid and incubated on ice for 30 min. Samples were centrifuged at 13,000 *g* for 5 min at 4°C and protein pellets resuspended in 1 ml of ice-cold acetone by sonication. Samples were then stored at − 20°C for at least 30 min before being centrifuged at 13,000 *g* for 5 min at 4°C. After removal of the acetone and drying, the protein pellets were resuspended in 80 μl of Laemmli sample buffer (2% SDS, 10% glycerol, 100 mM Tris-HCl, pH 6.8, 1% 2-mercaptoethanol, 0.002% bromophenol blue) and boiled before loading the samples on SDS-PAGE gels. Western blot membranes were probed with anti-Ape1 (1:3000 [64];), anti-FLAG (1:1000; Sigma Aldrich, F1804) and anti-Pgk1 (1:3000 [65];). The secondary antibodies used were Alexa Fluor^TM^ 680-conjugated goat anti-mouse-IgG or goat anti-rabbit IgG (1:7500; Thermo Fisher Scientific, A-21058 or A-21076). Detection of proteins and quantification of non-saturated images were performed using an Odyssey® Fc Imaging System (LI-COR Biosciences).

### Pho8∆60 assay

Yeast was grown to log phase in YPD medium and nitrogen starved in SD-N medium for 4 h. The equivalent of 5 OD_600_ of cells were harvested by centrifugation at 3,200 *g* for 5 min before and after starvation. Cells were then lysed in 200 μl of the lysis buffer (20 mM PIPES, pH 6.8, 0.5% Triton X-100 [Sigma-Aldrich, X100-500 ML], 50 mM KCl, 100 mM potassium acetate, 10 mM MgSO_4_, 10 µM ZnSO_4_, 2 mM PMSF [Sigma-Aldrich, P7626]) by adding 100 μl of glass beads (0.4–0.6 mm in diameter; VWR, 412–0069) and vortexing at 4°C for 5 min. Lysates were then centrifuged at 13,000 *g* for 5 min at 4°C. 50 μl supernatant were mixed with 200 μl alkaline phosphatase reaction buffer (250 mM Tris-HCl, pH 8.5, 0.4% Triton X-100, 10 mM MgSO_4_, 10 µM ZnSO_4_, an freshly added 1.25 mM *p*-nitrophenyl phosphate [Sigma-Aldrich, N2765-100TAB]) prewarmed at 37°C in 96 wells plate and the solution color was measured at 404 nm and 37°C using GloMax® Discover Microplate Reader (Promega, Madison, Wisconsin, USA) at 1-min intervals during 40 min. The protein concentration in the samples was determined using the BCA protein assay kit (Thermo Fisher Scientific 23,227) and the GloMax® Discover Microplate Reader following the manufacturer instructions. The Pho8∆60 activity was calculated as following: The blank was subtracted to all of values before to correct them with the protein concentration. The corrected values were then represented in a linear plot and the slope for each sample was determined as arbitrary units.

### APEX2-mediated PL followed by western blot

Cells were grown overnight to log phase in biotin-free SMD medium and nitrogen starved in biotin-free SD-N medium for the indicated time. Cells were then prepared and processed for APEX2-mediated PL using a modified version of a protocol previously described [39]. The equivalent of 25 OD_600_ equivalents of growing or nitrogen starved cells were harvested by centrifugation at 3,200 *g* for 2 min at 4°C. Upon collection, cells were immediately resuspended in 300 µl of freezing buffer (15% glycerol, 150 mM potassium acetate, 2 mM magnesium acetate, 20 mM HEPES/NaOH, pH 7.2, 1% glucose) and snap frozen in LN2 to semi-permeabilize them to allow BP uptake. Storage of snap frozen cells at − 80°C is possible for months without effecting biotin tagging. Cells were stored at − 80°C for at least 1 day and then thawed on ice, centrifuged at 16,200 *g* for 1 min at 4°C and upon discarding the supernatant, resuspended in 300 µl of freezing buffer at RT before immediately adding 1.2 µl of 125 mM BP (final concentration: 0.5 mM; Iris Biotech, LS-3500). After rapid but gentle mixing, 3.3 µl of 100 mM H_2_O_2_ (final concentration: 1 mM) was added and cells were gently mixed again. After a 1- or 3-min incubation at RT, 1 ml of ice-cold quenching buffer was added to stop the PL reaction. The quenching buffer was prepared by supplementing PBS (0.8% NaCl, 0.02% KCl, 0.15% Na_2_HPO_4,_ 0.024% KH_2_PO_4_, pH 7.2) with 10 mM sodium ascorbate, 10 mM NaN_3_ and 5 mM Trolox (Thermo Scientific 10,782,831). To remove the excess BP and phenoxyl radicals, cells were washed 5 times by resuspending them in 1 ml of ice-cold quenching buffer by gentle pipetting before centrifugation at 16,200 *g* for 1 min at 4°C. Cells were then resuspended in 50 μl of SDS-lysis buffer (5% SDS, 50 mM Tris-HCl, pH 9, 100 mM dithiothreitol) supplemented with 10 mM sodium ascorbate, 10 mM NaN_3_, 5 mM Trolox, 1 mM PMSF and 1× complete™ EDTA-free Protease Inhibitor Cocktail (Roche 11,836,170,001), and 25 µl of acid-washed glass beads were added. Cells were lysed by heating at 55°C for 30 min under agitation (700 rpm) using a thermo-shaker (VWR® Thermal Shake Touch, Thorofare, NJ, USA), with intervals every 10 min in which samples were vortexed at RT for 1 min. The lysates were diluted with 100 μl of RIPA buffer (50 mM Tris-HCl, pH 7.5, 150 mM NaCl, 0.1% SDS, 0.5% sodium deoxycholate [Thermo Scientific 218,590,250], 1% Triton X-100) supplemented with 10 mM sodium ascorbate, 10 mM NaN_3_, 5 mM Trolox, 1 mM PMSF and 1× cOmplete™ EDTA-free Protease Inhibitor Cocktail before being clarified by centrifugation at 17,000 *g* for 5 min at 4°C. For the total lysate control, 10% of the clarified lysates (i.e., 15 μl) were mixed with 45 µl of 4× Laemmli sample buffer and boiled for 5 min at 95°C. The remaining 90% of the clarified lysates was incubated with 100 μl of Pierce™ High-capacity Streptavidin agarose beads (Cytiva 17,511,301) to isolate biotinylated proteins. The streptavidin-conjugated agarose beads were pre-equilibrated by addition of 1 ml of ice-cold RIPA buffer and incubation on a rotating wheel at RT for 10 min before centrifugation at 400 *g* for 2 min at RT and removal of the supernatant. Then, 1 ml of RIPA buffer and 90% of the lysate (i.e., 135 μl) were added to the streptavidin-conjugated agarose beads, and the samples were incubated on a rotating wheel overnight at 4°C. Samples were centrifuged at 400 *g* for 2 min at 4°C and beads were washed twice with 1 ml of ice-cold RIPA buffer, once with 1 ml of ice-cold 1 M KCl, once with 1 ml of ice-cold 0.1 M Na_2_CO_3_ and once with 1 ml of ice-cold 2 M urea in 10 mM Tris-HCl (pH 8.0). All washes were done for 5 min on a rotating wheel at 4°C. Biotinylated proteins were eluted from the agarose beads by boiling for 5 min at 95°C in 80 µl 4× Laemmli sample buffer containing 2 mM biotin (Sigma-Aldrich, B4501-1 G). Both total lysates and affinity purified proteins were resolved by SDS – PAGE and proteins transferred onto polyvinylidene difluoride (PVDF; Millipore, IPFL85R) membranes before being detected with specific antibodies and visualized using an Odyssey system (Li-Cor Biosciences, Lincoln, NE, USA). The primary antibodies used were anti-biotin (1:3000; Rockland, 100–4198), anti-Atg8 (1:2000 [63];), anti-HA (1:1000; Cell Signaling Technology, 3724; clone C29F4) and anti-Pgk1. The secondary antibodies used were Alexa Fluor^TM^ 680-conjugated goat anti-mouse-IgG or goat anti-rabbit IgG (1:7500). Detection of proteins and quantification of non-saturated images were performed using the Odyssey® Fc Imaging System.

### APEX2-mediated PL followed by protein mass spectrometry

Cells were grown overnight to log phase in a biotin-free SMD medium and nitrogen starved in biotin-free SD-N medium for 1 h. APEX2-mediated PL was scaled up from the equivalent of 25 OD_600_ of cells to th equivalent of 100 OD_600_ of cells to have sufficient material for the MS analyses. Experimental triplicates were collected were harvested at the indicated times before and after nitrogen starvation by centrifugation at 3,200 *g* for 2 min at 4°C. The volume in which the harvested equivalent of 100 OD_600_ of cells was immediately resuspended was increased from 300 µl to 1 ml of freezing buffer. Cells were semi-permeabilized by snap freezing, thawed on ice and APEX2-mediated PL reaction was performed for 1 min as described for the small-scale reaction, except that cells were resuspended in 1 ml of freezing buffer at RT to which 4.6 µl of 125 mM BP and 12 µl of 100 mM H_2_O_2_ were added. The equivalent of 100 OD_600_ of cells in which H_2_O_2_ addition was omitted was also collected and processed as negative controls. The reaction was stopped and the excess BP and phenoxyl radicals were removed as described for APEX2-mediated PL by western blot. Then, aliquots of the equivalent of 25 OD_600_ of cells (i.e., 250 µl) from one of the experimental replicates were pelleted, lysed, and biotinylated proteins were affinity purified and processed for western blot as described above to verify that the biotinylation reaction had worked. The equivalent of 100 OD_600_ of cells from the other experimental replicates were obtained by centrifugation at 16,200 *g* for 1 min at 4°C and snap frozen in LN2 separately. Cells pellets were stored at − 80°C until subsequent proteomic analysis. Cell pellets for MS were lysed and biotinylated proteins were affinity purified as described for the western blot procedure, except that the beads were washed differently: once with 1 ml of ice-cold 1 M KCl, once with 1 ml of ice-cold 0.1 M Na_2_CO_3,_ once with 1 ml of ice-cold 2 M urea and finally once with 50 mM Tris-HCl (pH 7.5). Proteins were reduced on beads with 10 µl of 100 mM dithiothreitol (final concentration: 1 mM) at RT for 15 min before being alkylated with 10 µl of 550 mM iodoacetamide (final concentration: 5.5 mM) in the dark for 15 min. Proteins were subjected to on beads digestion with 2 µg of trypsin (Promega, VA9000) at 37°C overnight, which was stopped by acidification with 40 µl of 50% trifluoroacetic acid (TFA) (final concentration: 2%). The supernatants containing the peptides were desalted on STAGE tips, 200 µl tips self-packed with a C18 disc (Empore C18 47 mm membranes, 3 M, 2215; Merck 66,883-U). Samples were eluted from the STAGE tips in 50 µl of 80% acetonitrile mixed with 0.1% formic acid in water. Eluates were subsequently, dried in a SpeedVac (Thermo Fisher Scientific, Bremen, Germany) before being resuspend in 20 µl of 0.1% of formic acid in water (solvent A).

### Liquid chromatography tandem mass spectrometry (LC-MS/MS) and data processing

LC-MS/MS measurements were performed on an EASY-nLC^TM^ 1200 nano-flow UHPLC system (Thermo Fisher Scientific) coupled to a Q Exactive HF-X hybrid quadrupole-Orbitrap mass spectrometer (Thermo Fisher Scientific). Five µl of solubilized peptides in solvent A were separated on a fused silica HPLC column (i.e., 75-μm internal diameter column [Fused-silica PicoTip® emitter: SilicaTip™, New Objective] self-packed with ReproSil-Pur 120 C18-AQ, 1.9 μm [Dr. Maisch, r119.aq.] to a length of 20 cm) using a gradient of solvent A and solvent B (0.1% formic acid in 80% acetonitrile in water) from 5% solvent B to 30% over 85 min at a 250 nl/min flow rate. The spray voltage was set to 2.3 kV with a capillary temperature of 250°C. The mass spectrometer was operated in data independent acquisition (DIA) mode with MS scans acquired at a resolution of 120k covering a m/z range from 370 to 1200, followed by 35 consecutive MS/MS scan windows of 24 m/z acquired at a 30k resolution with 1 m/z overlap. Stepped normalized collision energy was set as 25.5, 27 and 30. MS raw data was analyzed with Spectronaut^R^ (version 16 February 220903.53000) [66] by searching against both *S. cerevisiae* proteome (Uniprot, March 2016) and common contaminants. Pulsar search was performed allowing for a maximum of 3 missed cleavages. Cysteine carbamidomethylation was set as fixed modification. Protein N-terminal acetylation and methionine oxidation were set as variable modifications. DIA analysis cross-run normalization was turned off. When not mentioned otherwise, all other settings were left to default Biognosys (BGS) factory settings.

### Proteomic data analysis

Spectronaut^R^ output was analyzed with an in-house Python^TM^ code. Protein quantity values were log_2_ transformed and samples were median normalized. Samples were grouped per timepoint and missing values in the negative control samples of each group were imputed with random values drawn from a normal distribution of a mean 1.8 lower than the sample distribution and a standard deviation of 0.3. Sample groups containing more than one missing value after imputation in the negative control were discarded from subsequent statistical analysis. Thus, values from at least 3 independent experiments were used to determine statistical significance. This was evaluated using two-tailed t-testing to calculate the p-values. FDR correction was performed using the Benjamini-Hochberg method. The MS data of the statistically significant hits are presented in Table S1. The significant hits were subsequently individually examined in Pubmed (https://pubmed.ncbi.nlm.nih.gov/) and Saccharomyces Genome Database (https://www.yeastgenome.org/) to determine whether the identified proteins have previously been associated with autophagy in yeast or have previously been described to be Atg8 or Atg9 interactors. This information is presented in Tables S2 (for APEX2-Atg8 in growing conditions), S3 (for APEX2-Atg8 in nitrogen starvation conditions), S4 (for Atg9-APEX2 in growing conditions) and S5 (for Atg9-APEX2 in nitrogen starvation conditions).

GO Terms enrichment was performed in R using the clusterProfiler package [67,68] using Biological Process terms in the hits that were enriched a minimum of 2-fold with a p-value below 0.05. PPINs were retrieved from STRING database [69] through the Cytoscape string app [70] with a confidence cutoff of 0.4 and illustrated in Cytoscape using the omics visualizer app [71]. Proteins selected for network representations were hits enriched with a p-value <0.05 and a minimum of 2-fold change in one of the timepoints, and a negative fold-change or missing value for the other timepoint.

## Supplementary Material

Supplemental Material

Supplementary figures R3.docx
