# Supplementary material for: An APEX2-based proximity-dependent biotinylation assay with temporal specificity to study protein interactions during autophagy in the yeast Saccharomyces cerevisiae
Source: Autophagy. 2024 Jul 3;20(10):2323–37. doi: 10.1080/15548627.2024.2366749 (PMC11423678; doi:10.1080/15548627.2024.2366749)
Supplement: Supplemental Material [file KAUP_A_2366749_SM8137.zip › Information_S1.pdf]

APEX2-mediated PL followed by western blot

Day 1

Semi-permeabilization of yeast cells by snap freezing

1. Harvest 25 OD_{600} equivalents of yeast cells expressing the APEX2 fusion protein of interest by centrifugation at 3,200 *g* for 2 min at 4°C.
2. Discard the supernatant, immediately resuspend the cell pellets in 300 µl of ice-cold Freezing buffer (see **Buffer solutions** section) and transfer the cells to ice-cold 2 ml microcentrifuge tubes.
3. Flash-freeze the resuspensions/tubes in liquid nitrogen and store them at -80°C at least 1 day.

II Pause point: After step 3, storage of snap frozen cells at -80°C is possible for months without affecting biotin phenol (BP) labelling efficiency.

Day 2

BP labelling

4. Freshly prepare the stock solutions of H_2O_2, Trolox and sodium ascorbate (See **Stock solutions** section).
5. Thaw yeast cells on ice, it takes approximately 10 min for 25 OD_{600}.
6. In the meantime, freshly prepare the ice-cold Quenching buffer (See **Buffer solutions** section).

⚠ **CRITICAL**: The Quenching buffer should be freshly prepared each time and used within 30 min.

7. Centrifuge the cells at 16,200 *g* for 1 min at 4°C, discard the supernatant and resuspend the cells in 300 µl of freezing buffer at room-temperature (RT).
8. Add 1.2 µl of 125 mM BP (final concentration: 0.5 mM) and mix gently. Make sure you also have a negative control in which BP is not added.
9. Immediately add 3.3 µl of 100 mM H_2O_2 (final concentration: 1 mM) and mix gently again. Make sure you also have a negative control in which H_2O_2 is not added.

   ⚠ **CRITICAL**: Use freshly prepared H_2O_2 stock solution (See **Stock Solutions** section).

10. Incubate for 1 min (or any other chosen time) at RT and stop the reaction by adding 1 ml of ice-cold Quenching buffer (See **Buffer solutions** section) and placing the tubes on ice before centrifuging at 16,200 *g* for 30-60 s at 4°C. Discard the supernatant.
11. Remove the excess BP and phenoxyl radicals by washing 5 times with 1 ml ice-cold Quenching buffer. Resuspend the cells in the Quenching buffer by pipetting up and down, do not vortex, and each time, centrifuge at 16,200 *g* for 30-60 s at 4°C. Discard the supernatant.

Cell lysis

12. Resuspend the 25 OD_{600} cell pellets in 50 µl of supplemented SDS-lysis buffer (See **Buffer solutions** section) and add 25 µl of glass beads.
13. Heat the resuspension at 55°C for 30 min under agitation (700 rpm) using a thermo-shaker to lyse cells. Every 10 min, samples are vortexed at RT for 1 min before placing them back in the thermos-shaker.
14. In the meantime, freshly prepare the sodium deoxycholate stock and the supplemented RIPA buffer (See **Buffer solutions** section).
15. Add 100 µl of supplemented RIPA buffer and vortex briefly.

16. Clarify the lysates by centrifugation at 17,000 *g* for 5 min at 4°C. Keep the clarified lysates on ice.
17. In the meantime, equilibrate streptavidin beads. Take 100 µl of streptavidin bead slurry and centrifuge at 400 *g* for 2 min at RT. Discard carefully the buffer in which the beads were suspended and add 1 ml of RIPA buffer, incubate them for 10 min at RT on a rotatory wheel and then put the equilibrated beads on ice.
18. Mix 15 µl of the clarified lysates (10%) with 45 µl of 4x Laemmli sample buffer (See **Buffer solutions** section) and boil for 5 min at 95°C.

Isolation of biotinylated proteins

19. Add the remaining 135 µl of the clarified lysates onto equilibrated streptavidin beads.
20. Incubate the slurry together with the clarified lysate on a rotating wheel overnight at 4°C.

Day 3

21. Prepare washing buffers (i.e, RIPA buffer, 1 M KCl, 0.1 M Na_2CO_3 and 2 M urea) and put them on ice (See **Stock solutions** and **Buffer solutions** section).
22. Centrifuge the beads at 400 *g* for 2 min at 4°C and discard the supernatant.
23. Wash the beads with a succession of twice 1 ml of ice-cold RIPA buffer, 1 ml of ice-cold 1 M KCl, 1 ml of ice-cold 0.1 M Na_2CO_3, 1 ml of ice-cold 2 M urea and again with 1 ml of ice-cold RIPA buffer by incubating on a rotating wheel for 5 min at 4°C and then centrifuging at 400 *g* for 2 min at 4°C. Discard supernatant.
24. Elute the biotinylated proteins from the beads by boiling them in 80 µl of 4x Laemmli sample buffer containing 2 mM biotin (See **Stock solutions** and **Buffer solutions** section) for 5 min at 95°C.

II Pause point: The eluates can be frozen and stored at -20°C or -80°C until western blotting.

Stock solutions

- 50% glycerol

Mix equal proportions of glycerol and milliQ water to achieve the desired volume. Sterilise and store at RT.

- 1 M potassium acetate (KOAc) (MW: 98.15)

Weight 24.54 g of KOAc and add 200 ml of milliQ water. Adjust the volume to 250 ml with milliQ water. Store at RT.

- 1 M magnesium acetate (MgOAc) (MW: 142.394)

Weight 35.6 g of MgOAc and add 200 ml of milliQ water. Adjust the volume to 250 ml with milliQ water. Store at RT.

- 0.1 M HEPES, pH 7.2. (MW: 238.30)

Weight 2.38 g of HEPES and add 80 ml of milliQ water. Adjust the pH to 7.2 with solid NaOH (1.5 pellets). Then adjust the volume to 100 ml with milliQ water. Store at RT.

⚠ **Note:** HEPES will not go into solution without NaOH. Add 1 pellet immediately, then proceed carefully to not go over the desired pH.

- 40% glucose

Weight 40 g of glucose and add 100 ml of milliQ water. Sterilise and store at RT.

- 125 mM biotin phenol in DMSO (MW: 363.47)

Weight 45.25 mg of BP and dissolve in 1 ml of DMSO. Aliquot 10-20 µl of the solution in tubes and store at -80°C. The 125 mM stock may need to be sonicated before use.

- 100 mM H_2O_2 (MW: 34.0147)

Dilute 10.2 µl of 30% H_2O_2 (98.8 M) with 989.8 µl of water to make a 100 mM stock.

⚠ **CRITICAL:** Do not store this solution, it must be freshly prepared before use.

- 1 M sodium ascorbate (MW: 198.11)

Weigh 198.11 mg of sodium ascorbate and dissolve in 1 ml of milliQ water to make 1 M stock.

⚠ **CRITICAL:** Do not store this solution, it must be freshly prepared before making the Quenching buffer

- 1 M NaN_3 (MW: 65.01)

Weigh 325.05 mg of sodium azide and dissolve in 5 ml of milliQ water to make a 1 M stock. Aliquots can be stored at -20°C for several months.

- 500 mM Trolox (MW: 250.29)

Weigh 125.144 mg of Trolox and dissolve in 1 ml of DMSO to make a 500 mM stock. Sonicate it well.

⚠ **CRITICAL:** Do not store this solution, it must be freshly prepared before making the Quenching buffer

- 10% SDS (MW: 288.372)

Weight 50 g and add 250 ml of milliQ water. Adjust the volume to 500 ml with water. Store at RT.

- 1 M Tris-HCl, pH 9 (MW: 121.14)

Weight 60.57 g and add 250 ml of milliQ water. Adjust the pH to 9 with HCl and adjust the volume to 500 ml milliQ water. Store at RT.

- 1 M dithiothreitol (DTT) (MW: 154.253)

Dissolve 1.54 g of DTT in 8 ml of milliQ water. Adjust volume to 10 ml with milliQ water, make 1 ml aliquots, and store wrapped in aluminum foil at -20°C.

- 100 mM PMSF (MW: 174.2)

Dissolve 0.174 g of PMSF in 10 ml of isopropanol. Make 500 µl aliquots. Store at -20° C.

- 25x complete EDTA-free protease inhibitor

Dissolve one cOmplete EDTA-free tablet in 2 ml of milliQ water. The stock solution can be stored at -20°C for at least 12 weeks.

- 1 M Tris-HCl, pH 7.5 (MW: 121.14)

Weight 60.57 g of Tris and add 250 ml of milliQ water. Adjust the pH to 7.5 with HCl and adjust the volume to 500 ml with milliQ water. Store at RT.

- 2.5 M NaCl (MW: 58.44)

Weight 73.05 g of NaCl and add 250 ml of milliQ water. Adjust the volume to 500 ml with milliQ water. Store at RT.

- 10 % sodium deoxycholate

Weight 0.1 g and add 900 µl of milliQ water. Adjust the volume to 1 ml with milliQ water.

⚠ **CRITICAL:** Do not store this solution, it must be freshly prepared and protected from light.

- 1 M KCl (MW: 74.55)

Weight 18.63 g and add 200 ml of milliQ water. Adjust the volume to 250 ml with milliQ water. Store at 4°C.

- 0.1 M Na_2CO_3 (MW: 105.99)

Weight 2.64 g and add 200 ml of milliQ water. Adjust the volume to 250 ml with milliQ water. Store at 4°C.

⚠ **Note:** this solution is not buffered, and it will give a pH of around 11.5.

- 1 M Tris-HCl, pH 8 (MW: 121.14)

Weight 60.57 grams and add 250 ml of milliQ water. Adjust the pH to 8 with HCl and adjust the volume to 500 ml with milliQ water. Store at RT.

- 100 mM biotin in DMSO (MW: 244. 31)

Weight 244.31 mg of biotin; dissolve in 1 ml of DMSO (final concentration: 100 mM). Make 1 ml aliquots and store them at -20 °C. These aliquots can be stored for several months.

Buffer solutions

- Freezing buffer

for 100 ml:

30 ml of 50% glycerol (final concentration: 15%)

15 ml of 1 M KOAc (final concentration: 150 mM)

200 µl of 1 M MgOAc (final concentration: 2 mM)

20 ml of 0.1 M HEPES/NaOH, pH 7.2 (final concentration: 20 mM)

1.25 ml of 40% glucose (final concentration: 1%)

Adjust to the final volume with milliQ water.

⚠ **Note:** Store part at 4°C, and part at RT.

⚠ **CRITICAL:** For long-term storage, this solution must be filter sterilised or prepared with sterile solutions.

- PBS buffer

for 100 ml:

0.8 g NaCl (final concentration: 0.8 %)

0.02 g KCl (final concentration: 0.02 %)

0.144 Na_2HPO_4 (final concentration: 0.144 %)

0.024 g KH_2PO_4 (final concentration: 0.024 %)

Adjust to the final volume with milliQ water.

⚠ **Note:** Set the pH to 7.2 with HCl and then autoclave.

- Quenching buffer (freshly made)

for 100 ml:

1 ml of 1 M sodium ascorbate (final concentration: 10 mM)

1 ml of 1 M NaN_3 (final concentration: 10 mM)

1 ml of 0.5 M Trolox (final concentration: 5 mM)

97 ml of PBS

⚠ **CRITICAL:** Do not store this solution, it must be freshly prepared before use.

- SDS-lysis buffer

for 100 ml:

50 ml of 10% SDS (final concentration: 5%)

5 ml of 1 M Tris/HCl, pH 9 (final concentration: 50 mM)

Supplemented with:

10 ml of 1 M DTT (final concentration: 100 mM)

1 ml of 1 M sodium ascorbate (final concentration: 10 mM)

1 ml of 1 M NaN_3 (final concentration: 10 mM)

1 ml of 0.5 M Trolox (final concentration: 5 mM)

1 ml of 100 mM PMSF (final concentration: 1 mM)

4 ml of 25x complete EDTA-free protease inhibitor (final concentration: 1X)

Adjust to the final volume with milliQ water.

⚠ **CRITICAL:** Do not store this solution, it must be freshly supplemented before use.

- RIPA buffer (store at 4°C)

for 100 ml:

5 ml of 1 M Tris-HCl, pH 7.5 (final concentration: 50 mM)

6 ml of 2.5 M NaCl (final concentration: 150 mM)

1 ml of 10% SDS (final concentration: 0.1%)

1 ml of 100% Triton X-100 (final concentration: 1%)

Supplemented with:

5 ml of 10% sodium deoxycholate (final concentration: 0.5%)1 ml of 1 M sodium ascorbate (final concentration: 10 mM)

1 ml of 1 M NaN_3 (final concentration: 10 mM)

1 ml of 0.5 M Trolox (final concentration: 5 mM)

1 ml of 100 mM PMSF (final concentration: 1 mM)

4 ml of 25X Complete EDTA-free protease inhibitor (final concentration: 1X)

Adjust to the final volume with milliQ water.

⚠ **CRITICAL:** Do not store this solution, must be freshly supplemented before use.

- 2 M urea in 10 mM Tris-HCl, pH 8 (MW: 60.06)

Weight 30.03 g of urea and add 2.5 ml of 1 M Tris-HCl pH 8 (final concentration 10 mM) and 240 ml of milliQ water. Adjust the volume to 250 ml with milliQ water.

⚠ **CRITICAL:** Do not store this solution, must be freshly prepared before use.

- 4x Laemmli sample buffer

for 100 ml:

35 ml of 1M Tris-HCl, pH 6.8 (final concentration 350 mM)

8 g of SDS (final concentration 8%)

40 ml of 100 % glycerol (final concentration 40%)

0.2 g of bromophenol blue (final concentration 0.2 %)

Supplemented with:

2 ml of 100 mM biotin (final concentration 2 mM)

10 ml ß-mercaptoethanol (final concentration 10%) or 2 ml of 1 M DTT (final concentration 20 mM)

Adjust to the final volume with milliQ water.

⚠ **Note:** Add biotin before adding the reducing agent (i.e., β-mercaptoethanol or DTT) to avoid its precipitation. Supplement before use.
