# Supplementary material for: An APEX2-based proximity-dependent biotinylation assay with temporal specificity to study protein interactions during autophagy in the yeast Saccharomyces cerevisiae
Source: Autophagy. 2024 Jul 3;20(10):2323–37. doi: 10.1080/15548627.2024.2366749 (PMC11423678; doi:10.1080/15548627.2024.2366749)
Supplement: Supplemental Material [file KAUP_A_2366749_SM8137.zip › Table_S2.docx]

# **Table S2. Atg8 interactors in rich medium.** Known roles of the detected proteins in yeast autophagy are indicated, as well if they were identified in other autophagy-related proteomics analyses.

| Enriched interactors (BH corrected p-value < 0.05) | | |
| --- | --- | --- |
| **Protein** | **Autophagy-related function(s) in yeast** | **Other MS analyses** |
| Atg2 | Atg machinery core component [1,2] | [3] |
| Bna2 | - |  |
| Cha1 | - | [3] |
| Coi1 | - |  |
| Elo1 |  |  |
| Erv1 | - | [3] |
| Hrr25 | Kinase regulating selective autophagy via phosphorylation of SARs [4-6] and macroautophagy via Sec24 [7]; regulated by Ypt1 [8] | [3] |
| Hxt1 | - | [3] |
| Mxr1 | - |  |
| Pai3 | Inhibitor of Pep4 [9], which is the major vacuolar protease essential for the degradation of autophagosomal cargoes [10] |  |
| Pdr5 | - | [3] |
| Pfd1 | - |  |
| Psg1 | - | [3] |
| Pun1 | - |  |
| Rpl42a; Rpl42b | Subunit of the 60S ribosome, which is selectively degraded by autophagy [11] |  |
| Rrp14 | - | [3] |
| Sco1 | - |  |
| Sco2 | - |  |
| Sft1 | Possibly involved in Atg9 trafficking [12] | [13] |
| Shr3 | - |  |
| Tif4631 | Possibly degraded in the vacuole upon autophagy induction [14] | [3] |
| Tim11 | - | [3] |
| Vam3 | SNARE involved in the fusion of autophagosomes with the vacuole [15]; binding partner of Atg8 [16] |  |
| Ycr090c | - |  |
| Yet3 | - | [3] |
| Ygr127w | - |  |
| Ynl208w | - |  |
| Enriched interactors (p-value < 0.05) | | |
| **Protein** | **Autophagy-related function(s) in yeast** | **Other MS analyses** |
| Abp1 | Cargo of Cue5-mediated aggrephagy [17]; regulates Arp2/3 complex-mediated actin assembly, which is involved in Atg9 trafficking [18] | [3] |
| Acb1 | Secretory autophagosome cargo [19]; negative regulator of autophagy [20] |  |
| Adh5 | - | [3] |
| Aim39 | - | [3] |
| Ams1 | Cvt pathway cargo [21] | [22] |
| Ape4 | Cvt pathway cargo [23] | [22] |
| Asn1 | Candidate autophagosomal cargo [24] | [3] |
| Atg3 | Atg machinery core component. E2-like enzyme involved in Atg8 conjugation to PE [25]; directly interacts with Atg8 [26] | [3,22] |
| Atg8 | Atg8 can self-interact [27] | [3,13] |
| Atg11 | Atg machinery core component, adaptor protein for all the selective types of autophagy [28-31] | [3] |
| Atg19 | Cvt pathway autophagy receptor [32]; directly binds Atg8 [33] | [3,22] |
| Atg21 | Atg machinery core component involved in the recruitment of the Atg12–Atg5-Atg16 complex to the PAS [34]; directly interacts with Atg8 [35] |  |
| Atg27 | Binds to Atg9 and it is involved in its trafficking [36] |  |
| Atg42 | Vacuolar protease, which is essential for the degradation of the autophagosomal cargoes [37] | [3] |
| Bcy1 | Negative regulatory subunit of PKA, which negatively regulates autophagy [38-42] | [3] |
| Bna1 | - | [3] |
| Bud27 | Possible regulator of autophagy induction via TORC1 [43] |  |
| Cab1 | - |  |
| Cbr1 | - | [3] |
| Ccp1 | - |  |
| Ccs1 | - | [3] |
| Cdc12 | - | [3] |
| Cdc28 | - | [3] |
| Cdc3 | - | [3] |
| Cdc33 | Translation initiation factor that interacts with Psp2 and Dhh1 through Eap1 and Ded1, which positively regulates autophagy by promoting Atg1 and Atg13 translation [44-47] | [3] |
| Cdc42 | - | [3] |
| Cff1 | - |  |
| Chc1 | Involved in Atg27 trafficking [48] and Ede1-mediated selective autophagy of aberrant CME protein condensates [3] | [3] |
| Cho2 | Involved in phosphatidylcholine biosynthesis, which is important for phagophore closure [49] | [3] |
| Cki1 | Involved in phosphatidylcholine biosynthesis, which is important for phagophore closure [49] |  |
| Cmc2 | - |  |
| Cnb1 | - |  |
| Cox6 | - | [3] |
| Cpr6 | - | [3] |
| Crm1 | - | [3] |
| Crs1 | - |  |
| Cse1 | - |  |
| Ctt1 | - |  |
| Cub1 | - | [3] |
| Cyt1 | - |  |
| Dcs1 | - |  |
| Ddr48 | - |  |
| Dys1 | - |  |
| Ecm4 | - |  |
| Ede1 | SAR for the selective autophagy of aberrant CME protein condensates [3] | [3] |
| Emi2 | - |  |
| End3 | Involved in Ede1-mediated selective autophagy of aberrant CME protein condensates [3]; required for ER-phagy [50] |  |
| Ent5 | Involved in Atg27 trafficking [48] | [3] |
| Erg8 | - |  |
| Etr1 | - |  |
| Fmp10 | - | [3] |
| Frs2 | Candidate autophagosomal cargo [24] | [3] |
| Fsf1 | - |  |
| Fsh1 | - |  |
| Gbp2 | - | [3] |
| Gcn1 | - | [3] |
| Get4 | - |  |
| Gfa1 | - | [3] |
| Gga1 | - |  |
| Glo2 | - |  |
| Glr1 | - | [3] |
| Gpd2 | - | [3] |
| Gre1 | - |  |
| Grh1 | Involved in secretory autophagy [19] |  |
| Grx2 | Candidate autophagosomal cargo [24] | [3] |
| Gut2 | - |  |
| Has1 | - |  |
| Hat2 | - | [3] |
| Hsp12 | - |  |
| Hxk1 | - |  |
| Hxt7 | Candidate autophagosomal cargo [51] |  |
| Igo1 | Phosphorylated Igo1 directly inhibits the Cdc55 phosphatase [52], which is required for sufficient Atg13 dephosphorylation and autophagy induction after TORC1 inactivation [53]; required for pre-meiotic autophagy [54] |  |
| Iki3 | - | [3] |
| Imd4 | - | [3] |
| Inp53/Sjl3 | Redundant with Sac1 and Ymr1 in the formation and maturation of autophagosomes, respectively [55,56] |  |
| Isd11 | - | [3] |
| Kes1 | Involved in regulating the Cvt pathway and autophagy by integrating lipid metabolism with TORC1 signaling and nitrogen sensing **[57,58]** | [3] |
| Lap2 | - |  |
| Lap3 | Cvt pathway cargo [59] |  |
| Lsb3 | - | [3] |
| Lsp1 | - | [3] |
| Lys2 | - |  |
| Mam3 | - | [3] |
| Map1 | - | [3] |
| Mbf1 | - |  |
| Mcr1 | - |  |
| Mdm35 | - |  |
| Mia40 | - | [3] |
| Mic60 | - |  |
| Mpm1 | - | [3] |
| Mrn1 | - | [3] |
| Mrp8 | - | [3] |
| Msc1 | - |  |
| Msc7 | - |  |
| Mtc1 | - | [3] |
| Nop2 | - | [3] |
| Npa3 | - | [3] |
| Nsg2 | - | [3] |
| Om45 | - | [3] |
| Ora1 | - |  |
| Osh7 | Involved in piecemeal microautophagy of the nucleus [60] | [3] |
| Pfk26 | - | [3] |
| Pho13 | - |  |
| Pin3 | - | [3] |
| Pma1 | - |  |
| Pmi40 | - |  |
| Pre2 | Subunit of the 26S proteasome, which is targeted by selective autophagy [61-63] | [3] |
| Prm8 | - |  |
| Prs5 | - | [3] |
| Ptk2 | - | [3] |
| Pub1 | Component of stress granules, which are degraded by autophagy [64] | [3] |
| Qri1 | - | [3] |
| Ras2 | Autophagy regulator [65] |  |
| Rci37 | - |  |
| Rdi1 | - |  |
| Rfc1 | - | [3] |
| Rio2 | - | [3] |
| Rli1 | - |  |
| Rnr2 | - | [3] |
| Rot2 | - |  |
| Rpg1 | - | [3] |
| Rpl27a | Subunit of the 60S ribosome, which is selectively degraded by autophagy [11]; candidate autophagosomal cargo [24] |  |
| Rpl33a | Subunit of the 60S ribosome, which is selectively degraded by autophagy [11]; candidate autophagosomal cargo [24] | [3] |
| Rpl7a | Subunit of the 60S ribosome, which is selectively degraded by autophagy [11]; candidate autophagosomal cargo [24] | [3] |
| Rpl7b | Subunit of the 60S ribosome, which is selectively degraded by autophagy [11]; candidate autophagosomal cargo [24] | [3] |
| Rpl8b | Subunit of the 60S ribosome, which is selectively degraded by autophagy [11]; candidate autophagosomal cargo [24] | [3] |
| Rpn5 | Subunit of the 26S proteasome, which is targeted by selective autophagy [61-63] |  |
| Rpn6 | Subunit of the 26S proteasome, which is targeted by selective autophagy [61-63] | [3] |
| Rpn7 | Subunit of the 26S proteasome, which is targeted by selective autophagy [61-63] | [3] |
| Rps11A; Rps11B | - |  |
| Rps23A; Rps23B | - |  |
| Rps31 | - | [3] |
| Rsc8 | Subunit of the Rsc1-RSC chromatin remodeling complex, which is required for autophagy induction [66] | [3] |
| Rsp5 | Ubiquitin ligase involved in the selective autophagy of aggregates, proteasomes, mitochondria and possibly ribosomes [17,63,67,68]; involved in microautophagy of vacuolar membrane proteins and proteasomes [69,70] | [3] |
| Rtc3 | - |  |
| Rtn2 | - | [3] |
| Rvb2 | Helicase subunit of the Ino80-chromatin remodeling complex, which is involved in the transcriptional repression of *ATG* genes [71] | [3] |
| Sap185 | - | [3] |
| Scs2 | Involved in ER-phagy [50] | [3] |
| Sec16 | Subunit of COPII vesicles, which are a membrane source for autophagosome biogenesis [13,72,73] and are involved in Atg9 sorting out of the ER [74] | [3,13] |
| Sec27 | - | [3] |
| Sec9 | Involved in Atg9 trafficking [75] | [3] |
| Shp1 | Binding partner of Atg8 involved in autophagosome formation [76] |  |
| Slf1 | - | [3] |
| Sna4 | Vacuolar protein degraded by microautophagy [77] |  |
| Snx3 | - |  |
| Atg24 | Mediates lipid trafficking promoting autophagy and vacuole membrane fusion [78]; involved in the Cvt pathway [79], proteaphagy [80], possibly mitophagy [81], Atg27 retrograde trafficking/recycling from vacuoles [82,83] and vacuolar targeting of transcription factors controlling *ATG* gene expression [84]; interacts with the Atg1 kinase complex [79] | [3] |
| Spe3 | - |  |
| Sse2 | - |  |
| Sss1 | - | [3] |
| Sti1 | Candidate autophagosomal cargo [24] |  |
| Stm1 | Upon autophagy induction, acts as a 80S ribosome preservation factor [85] | [3] |
| Sui2 | - | [3] |
| Sui3 | - | [3] |
| Svf1 | - | [3] |
| Thi20 | - |  |
| Tif3 | - | [3] |
| Tif35 | - | [3] |
| Tif6 | - | [3] |
| Tim23 | - | [3] |
| Tma22 | - | [3] |
| Tmt1 | - |  |
| Tpk2 | Catalytic subunit of PKA, which regulates autophagy [39,41,86] |  |
| Tpm2 | - |  |
| Tps3 | - | [3] |
| Trm1 | - | [3] |
| Tub1 | - | [3] |
| Ty1b-pr2; Ty1b-m11 | Cvt pathway cargo [87] |  |
| Uba4 | - | [3] |
| Ubc1 | E2 conjugating enzyme involved in autophagic degradation of 26S proteasomes [63] |  |
| Ugo1 | - |  |
| Uip4 | - |  |
| Ura6 | - | [3] |
| Utr2 | - |  |
| Utr4 | - |  |
| Vht1 | - |  |
| Vma6 | Subunit of the V-ATPase involved in acidification of the vacuolar lumen, which is essential for the degradation of autophagosomal cargoes [88] |  |
| Vps24 | ESCRT-III component possibly involved in autophagosome closure [89], microautophagy [77] and secretory autophagy [90] |  |
| Vps35 | Involved in Atg9 trafficking [91] |  |
| Vps74 | Phosphatidylinositol-4-phosphate binding protein (PtdIns4P) that interacts with the catalytic domain of Sac1 regulating its function [92]; Sac1 restrains PtdIns4P accumulation in autophagosomes, which inhibits their fusion with vacuoles [93] |  |
| Wwm1 | - | [3] |
| Ydl086w | - |  |
| Ydr391c | - |  |
| Yet1 | - | [3] |
| Yjr154w | - |  |
| Ykt6 | SNARE involved in Atg9 trafficking and autophagosome fusion with vacuoles [75,94,95] |  |
| Yml131w | - |  |
| Ymr090w | - |  |
| Yor289w | - |  |
| Ypt1 | Essential for autophagy progression [96]; recruited to the PAS by Atg9 vesicles and the TRAPPIII complex [96,97] | [13] |
| Ypt31 | Important for autophagy progression [98] | [13] |
| Zeo1 | - |  |

# **References**

1. Shintani T, Suzuki K, Kamada Y, et al. Apg2p functions in autophagosome formation on the perivacuolar structure. J Biol Chem. 2001 Aug 10;276(32):30452-60.

2. Wang CW, Kim J, Huang WP, et al. Apg2 is a novel protein required for the cytoplasm to vacuole targeting, autophagy, and pexophagy pathways. J Biol Chem. 2001 Aug 10;276(32):30442-51.

3. Wilfling F, Lee CW, Erdmann PS, et al. A Selective Autophagy Pathway for Phase-Separated Endocytic Protein Deposits. Mol Cell. 2020 Dec 3;80(5):764-778 e7.

4. Pfaffenwimmer T, Reiter W, Brach T, et al. Hrr25 kinase promotes selective autophagy by phosphorylating the cargo receptor Atg19. EMBO Rep. 2014 Aug;15(8):862-70.

5. Tanaka C, Tan LJ, Mochida K, et al. Hrr25 triggers selective autophagy-related pathways by phosphorylating receptor proteins. J Cell Biol. 2014 Oct 13;207(1):91-105.

6. Mochida K, Ohsumi Y, Nakatogawa H. Hrr25 phosphorylates the autophagic receptor Atg34 to promote vacuolar transport of alpha-mannosidase under nitrogen starvation conditions. FEBS Lett. 2014 Nov 3;588(21):3862-9.

7. Davis S, Wang J, Zhu M, et al. Sec24 phosphorylation regulates autophagosome abundance during nutrient deprivation. Elife. 2016 Nov 18;5.

8. Wang J, Davis S, Menon S, et al. Ypt1/Rab1 regulates Hrr25/CK1delta kinase activity in ER-Golgi traffic and macroautophagy. J Cell Biol. 2015 Jul 20;210(2):273-85.

9. Schu P, Wolf DH. The proteinase yscA-inhibitor, IA3, gene. Studies of cytoplasmic proteinase inhibitor deficiency on yeast physiology. FEBS Lett. 1991 May 20;283(1):78-84.

10. Takeshige K, Baba M, Tsuboi S, et al. Autophagy in yeast demonstrated with proteinase-deficient mutants and conditions for its induction. Journal of Cell Biology. 1992;119(2):301-311.

11. Kraft C, Deplazes A, Sohrmann M, et al. Mature ribosomes are selectively degraded upon starvation by an autophagy pathway requiring the Ubp3p/Bre5p ubiquitin protease. Nat Cell Biol. 2008 May;10(5):602-10.

12. Zou S, Sun D, Liang Y. The Roles of the SNARE Protein Sed5 in Autophagy in Saccharomyces cerevisiae. Mol Cells. 2017 Sep 30;40(9):643-654.

13. Graef M, Friedman JR, Graham C, et al. ER exit sites are physical and functional core autophagosome biogenesis components. Mol Biol Cell. 2013 Sep;24(18):2918-31.

14. Berset C, Trachsel H, Altmann M. The TOR (target of rapamycin) signal transduction pathway regulates the stability of translation initiation factor eIF4G in the yeast *Saccharomyces cerevisia*. Proceedings of the National Academy of Sciences. 1998;95(8):4264-4269.

15. Darsow T, Rieder SE, Emr SD. A Multispecificity Syntaxin Homologue, Vam3p, Essential for Autophagic and Biosynthetic Protein Transport to the Vacuole. Journal of Cell Biology. 1997;138(3):517-529.

16. Legesse-Miller A, Sagiv Y, Glozman R, et al. Aut7p, a Soluble Autophagic Factor, Participates in Multiple Membrane Trafficking Processes. Journal of Biological Chemistry. 2000;275(42):32966-32973.

17. Lu K, Psakhye I, Jentsch S. Autophagic clearance of polyQ proteins mediated by ubiquitin-Atg8 adaptors of the conserved CUET protein family. Cell. 2014 Jul 31;158(3):549-63.

18. Monastyrska I, He C, Geng J, et al. Arp2 Links Autophagic Machinery with the Actin Cytoskeleton. Molecular Biology of the Cell. 2008;19(5):1962-1975.

19. Duran JM, Anjard C, Stefan C, et al. Unconventional secretion of Acb1 is mediated by autophagosomes. J Cell Biol. 2010 Feb 22;188(4):527-36.

20. Montegut L, Joseph A, Chen H, et al. DBI/ACBP is a targetable autophagy checkpoint involved in aging and cardiovascular disease. Autophagy. 2023 Jul;19(7):2166-2169.

21. Hutchins MU, Klionsky DJ. Vacuolar Localization of Oligomeric α-Mannosidase Requires the Cytoplasm to Vacuole Targeting and Autophagy Pathway Components in Saccharomyces cerevisiae. Journal of Biological Chemistry. 2001;276(23):20491-20498.

22. Tomioka Y, Kotani T, Kirisako H, et al. TORC1 inactivation stimulates autophagy of nucleoporin and nuclear pore complexes. J Cell Biol. 2020 Jul 6;219(7).

23. Yuga M, Gomi K, Klionsky DJ, et al. Aspartyl Aminopeptidase Is Imported from the Cytoplasm to the Vacuole by Selective Autophagy in Saccharomyces cerevisiae. Journal of Biological Chemistry. 2011;286(15):13704-13713.

24. Suzuki K, Nakamura S, Morimoto M, et al. Proteomic profiling of autophagosome cargo in Saccharomyces cerevisiae. PLoS One. 2014;9(3):e91651.

25. Ichimura Y, Kirisako T, Takao T, et al. A ubiquitin-like system mediates protein lipidation. Nature. 2000;408(6811):488-492.

26. Yamaguchi M, Noda NN, Nakatogawa H, et al. Autophagy-related protein 8 (Atg8) family interacting motif in Atg3 mediates the Atg3-Atg8 interaction and is crucial for the cytoplasm-to-vacuole targeting pathway. J Biol Chem. 2010 Sep 17;285(38):29599-607.

27. Nakatogawa H, Ichimura Y, Ohsumi Y. Atg8, a ubiquitin-like protein required for autophagosome formation, mediates membrane tethering and hemifusion. Cell. 2007 Jul 13;130(1):165-78.

28. Shintani T, Huang W-P, Stromhaug PE, et al. Mechanism of Cargo Selection in the Cytoplasm to Vacuole Targeting Pathway. Developmental Cell. 2002;3(6):825-837.

29. Suzuki K, Kamada Y, Ohsumi Y. Studies of Cargo Delivery to the Vacuole Mediated by Autophagosomes in Saccharomyces cerevisiae. Developmental Cell. 2002;3(6):815-824.

30. He C, Song H, Yorimitsu T, et al. Recruitment of Atg9 to the preautophagosomal structure by Atg11 is essential for selective autophagy in budding yeast. J Cell Biol. 2006 Dec 18;175(6):925-35.

31. Matscheko N, Mayrhofer P, Rao Y, et al. Atg11 tethers Atg9 vesicles to initiate selective autophagy. PLoS Biol. 2019 Jul;17(7):e3000377.

32. Scott SV, Guan J, Hutchins MU, et al. Cvt19 Is a Receptor for the Cytoplasm-to-Vacuole Targeting Pathway. Molecular Cell. 2001;7(6):1131-1141.

33. Abert C, Kontaxis G, Martens S. Accessory Interaction Motifs in the Atg19 Cargo Receptor Enable Strong Binding to the Clustered Ubiquitin-related Atg8 Protein. J Biol Chem. 2016 Sep 2;291(36):18799-808.

34. Harada K, Kotani T, Kirisako H, et al. Two distinct mechanisms target the autophagy-related E3 complex to the pre-autophagosomal structure. Elife. 2019 Feb 27;8.

35. Juris L, Montino M, Rube P, et al. PI3P binding by Atg21 organises Atg8 lipidation. EMBO J. 2015 Apr 1;34(7):955-73.

36. Legakis JE, Yen W-L, Klionsky DJ. A Cycling Protein Complex Required for Selective Autophagy. Autophagy. 2007;3(5):422-432.

37. Parzych KR, Ariosa A, Mari M, et al. A newly characterized vacuolar serine carboxypeptidase, Atg42/Ybr139w, is required for normal vacuole function and the terminal steps of autophagy in the yeast Saccharomyces cerevisiae. Mol Biol Cell. 2018 May 1;29(9):1089-1099.

38. Johnson KE, Cameron S, Toda T, et al. Expression in Escherichia coli of BCY1, the regulatory subunit of cyclic AMP-dependent protein kinase from Saccharomyces cerevisiae. Purification and characterization. Journal of Biological Chemistry. 1987;262(18):8636-8642.

39. Stephan JS, Yeh Y-Y, Ramachandran V, et al. The Tor and PKA signaling pathways independently target the Atg1/Atg13 protein kinase complex to control autophagy. Proceedings of the National Academy of Sciences. 2009;106(40):17049-17054.

40. Soulard A, Cremonesi A, Moes S, et al. The rapamycin-sensitive phosphoproteome reveals that TOR controls protein kinase A toward some but not all substrates. Mol Biol Cell. 2010 Oct 1;21(19):3475-86.

41. Yorimitsu T, Zaman S, Broach JR, et al. Protein kinase A and Sch9 cooperatively regulate induction of autophagy in Saccharomyces cerevisiae. Mol Biol Cell. 2007 Oct;18(10):4180-9.

42. Schmelzle T, Beck T, Martin DE, et al. Activation of the RAS/cyclic AMP pathway suppresses a TOR deficiency in yeast. Mol Cell Biol. 2004 Jan;24(1):338-51.

43. Gutierrez-Santiago F, Cintas-Galan M, Martin-Exposito M, et al. A High-Copy Suppressor Screen Reveals a Broad Role of Prefoldin-like Bud27 in the TOR Signaling Pathway in Saccharomyces cerevisiae. Genes (Basel). 2022 Apr 24;13(5).

44. Yin Z, Liu X, Ariosa A, et al. Psp2, a novel regulator of autophagy that promotes autophagy-related protein translation. Cell Res. 2019 Dec;29(12):994-1008.

45. Liu X, Yao Z, Jin M, et al. Dhh1 promotes autophagy-related protein translation during nitrogen starvation. PLoS Biol. 2019 Apr;17(4):e3000219.

46. Lahiri V, Metur SP, Hu Z, et al. Post-transcriptional regulation of ATG1 is a critical node that modulates autophagy during distinct nutrient stresses. Autophagy. 2022 Jul;18(7):1694-1714.

47. Gulay S, Gupta N, Lorsch JR, et al. Distinct interactions of eIF4A and eIF4E with RNA helicase Ded1 stimulate translation in vivo. Elife. 2020 May 29;9.

48. Segarra VA, Sharma A, Lemmon SK. Atg27p localization is clathrin- and Ent3p/5p-dependent. MicroPubl Biol. 2021 Published 2021 Mar 29.(2578-9430 (Electronic)).

49. Polyansky A, Shatz O, Fraiberg M, et al. Phospholipid imbalance impairs autophagosome completion. EMBO J. 2022 Dec 1;41(23):e110771.

50. Liu D, Mari M, Li X, et al. ER-phagy requires the assembly of actin at sites of contact between the cortical ER and endocytic pits. Proc Natl Acad Sci U S A. 2022 Feb 8;119(6).

51. Krampe S, Boles E. Starvation-induced degradation of yeast hexose transporter Hxt7p is dependent on endocytosis, autophagy and the terminal sequences of the permease. FEBS Lett. 2002 Feb 27;513(2-3):193-6.

52. Bontron S, Jaquenoud M, Vaga S, et al. Yeast endosulfines control entry into quiescence and chronological life span by inhibiting protein phosphatase 2A. Cell Rep. 2013 Jan 31;3(1):16-22.

53. Yeasmin AM, Waliullah TM, Kondo A, et al. Orchestrated Action of PP2A Antagonizes Atg13 Phosphorylation and Promotes Autophagy after the Inactivation of TORC1. PLoS One. 2016;11(12):e0166636.

54. Sarkar S, Dalgaard JZ, Millar JB, et al. The Rim15-endosulfine-PP2ACdc55 signalling module regulates entry into gametogenesis and quiescence via distinct mechanisms in budding yeast. PLoS Genet. 2014 Jun;10(6):e1004456.

55. Muramoto M, Yamakuchi Y, Konishi R, et al. Essential roles of phosphatidylinositol 4-phosphate phosphatases Sac1p and Sjl3p in yeast autophagosome formation. Biochim Biophys Acta Mol Cell Biol Lipids. 2022 Sep;1867(9):159184.

56. Cebollero E, van der Vaart A, Zhao M, et al. Phosphatidylinositol-3-phosphate clearance plays a key role in autophagosome completion. Curr Biol. 2012 Sep 11;22(17):1545-53.

57. LeBlanc MA, McMaster CR. Lipid binding requirements for oxysterol-binding protein Kes1 inhibition of autophagy and endosome-trans-Golgi trafficking pathways. J Biol Chem. 2010 Oct 29;285(44):33875-84.

58. Mousley CJ, Yuan P, Gaur NA, et al. A sterol-binding protein integrates endosomal lipid metabolism with TOR signaling and nitrogen sensing. Cell. 2012 Feb 17;148(4):702-15.

59. Kageyama T, Suzuki K, Ohsumi Y. Lap3 is a selective target of autophagy in yeast, Saccharomyces cerevisiae. Biochem Biophys Res Commun. 2009 Jan 16;378(3):551-7.

60. Kvam E, Goldfarb DS. Nvj1p is the outer-nuclear-membrane receptor for oxysterol-binding protein homolog Osh1p in Saccharomyces cerevisiae. J Cell Sci. 2004 Oct 1;117(Pt 21):4959-68.

61. Waite KA, De-La Mota-Peynado A, Vontz G, et al. Starvation Induces Proteasome Autophagy with Different Pathways for Core and Regulatory Particles. J Biol Chem. 2016 Feb 12;291(7):3239-53.

62. Marshall RS, McLoughlin F, Vierstra RD. Autophagic Turnover of Inactive 26S Proteasomes in Yeast Is Directed by the Ubiquitin Receptor Cue5 and the Hsp42 Chaperone. Cell Rep. 2016 Aug 9;16(6):1717-1732.

63. Marshall RS, Vierstra RD. A trio of ubiquitin ligases sequentially drives ubiquitylation and autophagic degradation of dysfunctional yeast proteasomes. Cell Rep. 2022 Mar 15;38(11):110535.

64. Buchan JR, Kolaitis RM, Taylor JP, et al. Eukaryotic stress granules are cleared by autophagy and Cdc48/VCP function. Cell. 2013 Jun 20;153(7):1461-74.

65. Budovskaya YV, Stephan JS, Reggiori F, et al. The Ras/cAMP-dependent protein kinase signaling pathway regulates an early step of the autophagy process in Saccharomyces cerevisiae. J Biol Chem. 2004 May 14;279(20):20663-71.

66. Yu F, Imamura Y, Ueno M, et al. The yeast chromatin remodeler Rsc1-RSC complex is required for transcriptional activation of autophagy-related genes and inhibition of the TORC1 pathway in response to nitrogen starvation. Biochem Biophys Res Commun. 2015 Sep 4;464(4):1248-1253.

67. Belgareh-Touze N, Cavellini L, Cohen MM. Ubiquitination of ERMES components by the E3 ligase Rsp5 is involved in mitophagy. Autophagy. 2017 Jan 2;13(1):114-132.

68. Kraft C, Peter M. Is the Rsp5 ubiquitin ligase involved in the regulation of ribophagy? Autophagy. 2008 Aug;4(6):838-40.

69. Yang X, Zhang W, Wen X, et al. TORC1 regulates vacuole membrane composition through ubiquitin- and ESCRT-dependent microautophagy. J Cell Biol. 2020 Mar 2;219(3).

70. Li J, Hochstrasser M. Selective microautophagy of proteasomes is initiated by ESCRT-0 and is promoted by proteasome ubiquitylation. J Cell Sci. 2022 Feb 15;135(4).

71. Li XA-O, Mei Q, Yu Q, et al. The TORC1 activates Rpd3L complex to deacetylate Ino80 and H2A.Z and repress autophagy. Sci Adv. 2023;9(2375-2548 (Electronic)):eade8312.

72. Suzuki K, Kubota Y, Sekito T, et al. Hierarchy of Atg proteins in pre-autophagosomal structure organization. Genes Cells. 2007 Feb;12(2):209-18.

73. Tan D, Cai Y, Wang J, et al. The EM structure of the TRAPPIII complex leads to the identification of a requirement for COPII vesicles on the macroautophagy pathway. Proc Natl Acad Sci U S A. 2013 Nov 26;110(48):19432-7.

74. Mari M, Griffith J, Rieter E, et al. An Atg9-containing compartment that functions in the early steps of autophagosome biogenesis. J Cell Biol. 2010 Sep 20;190(6):1005-22.

75. Nair U, Jotwani A, Geng J, et al. SNARE proteins are required for macroautophagy. Cell. 2011 Jul 22;146(2):290-302.

76. Krick R, Bremer S, Welter E, et al. Cdc48/p97 and Shp1/p47 regulate autophagosome biogenesis in concert with ubiquitin-like Atg8. J Cell Biol. 2010 Sep 20;190(6):965-73.

77. Morshed S, Tasnin MN, Ushimaru T. ESCRT machinery plays a role in microautophagy in yeast. BMC Mol Cell Biol. 2020 Oct 7;21(1):70.

78. Ma M, Kumar S, Purushothaman L, et al. Lipid trafficking by yeast Snx4 family SNX-BAR proteins promotes autophagy and vacuole membrane fusion. Mol Biol Cell. 2018 Sep 1;29(18):2190-2200.

79. Nice DC, Sato TK, Stromhaug PE, et al. Cooperative binding of the cytoplasm to vacuole targeting pathway proteins, Cvt13 and Cvt20, to phosphatidylinositol 3-phosphate at the pre-autophagosomal structure is required for selective autophagy. J Biol Chem. 2002 Aug 16;277(33):30198-207.

80. Nemec AA, Howell LA, Peterson AK, et al. Autophagic clearance of proteasomes in yeast requires the conserved sorting nexin Snx4. J Biol Chem. 2017 Dec 29;292(52):21466-21480.

81. Okamoto K, Kondo-Okamoto N, Ohsumi Y. Mitochondria-anchored receptor Atg32 mediates degradation of mitochondria via selective autophagy [Research Support, Non-U.S. Gov't]. Dev Cell. 2009 Jul;17(1):87-97.

82. Ma M, Burd CG, Chi RJ. Distinct complexes of yeast Snx4 family SNX-BARs mediate retrograde trafficking of Snc1 and Atg27. Traffic. 2017 Feb;18(2):134-144.

83. Suzuki SW, Emr SD. Retrograde trafficking from the vacuole/lysosome membrane. Autophagy. 2018;14(9):1654-1655.

84. Hanley SE, Willis SD, Cooper KF. Snx4-assisted vacuolar targeting of transcription factors defines a new autophagy pathway for controlling ATG expression. Autophagy. 2021 Nov;17(11):3547-3565.

85. Van Dyke N, Chanchorn E, Van Dyke MW. The Saccharomyces cerevisiae protein Stm1p facilitates ribosome preservation during quiescence. Biochem Biophys Res Commun. 2013 Jan 11;430(2):745-50.

86. Yu Q, Gong X, Tong Y, et al. Phosphorylation of Jhd2 by the Ras-cAMP-PKA(Tpk2) pathway regulates histone modifications and autophagy. Nat Commun. 2022 Sep 27;13(1):5675.

87. Suzuki K, Morimoto M, Kondo C, et al. Selective autophagy regulates insertional mutagenesis by the Ty1 retrotransposon in Saccharomyces cerevisiae. Dev Cell. 2011 Aug 16;21(2):358-65.

88. Nakamura N, Matsuura A, Wada Y, et al. Acidification of Vacuoles Is Required for Autophagic Degradation in the Yeast, *Saccharomyces cerevisiae*. The Journal of Biochemistry. 1997;121(2):338-344.

89. Zhou F, Wu Z, Zhao M, et al. Rab5-dependent autophagosome closure by ESCRT. J Cell Biol. 2019 Jun 3;218(6):1908-1927.

90. Bruns C, McCaffery JM, Curwin AJ, et al. Biogenesis of a novel compartment for autophagosome-mediated unconventional protein secretion. J Cell Biol. 2011 Dec 12;195(6):979-92.

91. Marquardt L, Taylor M, Kramer F, et al. Vacuole fragmentation depends on a novel Atg18-containing retromer-complex. Autophagy. 2023 Jan;19(1):278-295.

92. Wood CS, Hung CS, Huoh YS, et al. Local control of phosphatidylinositol 4-phosphate signaling in the Golgi apparatus by Vps74 and Sac1 phosphoinositide phosphatase. Mol Biol Cell. 2012 Jul;23(13):2527-36.

93. Zhang H, Zhou J, Xiao P, et al. PtdIns4P restriction by hydrolase SAC1 decides specific fusion of autophagosomes with lysosomes. Autophagy. 2021 Aug;17(8):1907-1917.

94. Gao J, Reggiori F, Ungermann C. A novel in vitro assay reveals SNARE topology and the role of Ykt6 in autophagosome fusion with vacuoles. J Cell Biol. 2018 Oct 1;217(10):3670-3682.

95. Bas L, Papinski D, Licheva M, et al. Reconstitution reveals Ykt6 as the autophagosomal SNARE in autophagosome-vacuole fusion. J Cell Biol. 2018 Oct 1;217(10):3656-3669.

96. Lynch-Day MA, Bhandari D, Menon S, et al. Trs85 directs a Ypt1 GEF, TRAPPIII, to the phagophore to promote autophagy. Proc Natl Acad Sci U S A. 2010 Apr 27;107(17):7811-6.

97. Kakuta S, Yamamoto H, Negishi L, et al. Atg9 vesicles recruit vesicle-tethering proteins Trs85 and Ypt1 to the autophagosome formation site. J Biol Chem. 2012 Dec 28;287(53):44261-9.

98. Zou S, Chen Y, Liu Y, et al. Trs130 participates in autophagy through GTPases Ypt31/32 in Saccharomyces cerevisiae. Traffic. 2013 Feb;14(2):233-46.
