# Supplementary material for: An APEX2-based proximity-dependent biotinylation assay with temporal specificity to study protein interactions during autophagy in the yeast Saccharomyces cerevisiae
Source: Autophagy. 2024 Jul 3;20(10):2323–37. doi: 10.1080/15548627.2024.2366749 (PMC11423678; doi:10.1080/15548627.2024.2366749)
Supplement: Supplemental Material [file KAUP_A_2366749_SM8137.zip › Table_S3.docx]

**Table S3. Atg8 interactors upon 1 h of nitrogen starvation.** Known roles of the detected proteins in yeast autophagy are indicated, as well if they were identified in other autophagy-related proteomics analyses.

| Enriched interactors (BH corrected p-value < 0.05) | | |
| --- | --- | --- |
| **Protein** | **Autophagy-related function(s) in yeast** | **Other MS analyses** |
| Abz2 | - |  |
| Ade1 | - |  |
| Adh5 | - | [1] |
| Akl1 | - | [1] |
| Ala1 | Candidate autophagosomal cargo [2] | [1] |
| Aro3 | - |  |
| Atg2 | Atg machinery core component [3,4] | [1] |
| Atg21 | Atg machinery core component involved in the recruitment of the Atg12–Atg5-Atg16 complex to the PAS [5]. It directly interacts with Atg8 [6] |  |
| Atp4 | - |  |
| Bet4 | Involved in the geranylisation of Ypt1 and Sec4 proteins [7], which are involved in autophagy [8,9] |  |
| Bfr1 | - |  |
| Bna2 | - |  |
| Ccp1 | - |  |
| Cdc42 | - | [1] |
| Chc1 | Involved in Atg27 trafficking [10] and in Ede1-mediated selective autophagy of aberrant CME protein condensates [1] |  |
| Clu1 | - | [1] |
| Cmc2 | - |  |
| Cnb1 | - |  |
| Cop1 | - | [1] |
| Cpa2 | - | [1] |
| Cub1 | - | [1] |
| Ddr48 | - |  |
| Dnm1 | Involved in mitophagy and pexophagy, via an interaction with Atg11 [11,12] | [1] |
| Efb1 | Candidate autophagosomal cargo [2] | [1] |
| Erg11 | - | [1] |
| Erg5 | - | [1] |
| Erg8 | - |  |
| Glc8 | - |  |
| Gpd2 | - | [1] |
| Gpp1 | - | [1] |
| Grx2 | - | [1] |
| Gvp36 | Cargo of Cue5-mediated aggrephagy [13]. As Atg9, involved in sphingolipid homeostasis [14] | [1,15] |
| Hom3 | - | [1] |
| Hse1 | Together with Vps27, required for microautophagy induction [16] | [1] |
| Hsp104 | Candidate autophagosomal cargo [2] | [1] |
| Hsp12 | - |  |
| Hsp42 | Involved in proteasome turnover by selective autophagy [17,18] | [1] |
| Hsp60 | - | [1] |
| Hsp78 | - | [1] |
| Hxk1 | - |  |
| Kap123 | Candidate autophagosomal cargo [2] | [1] |
| Kap95 | Candidate autophagosomal cargo [2] | [1] |
| Lsp1 | - | [1] |
| Mic60 | - |  |
| Mns1 | - |  |
| Mri1 | - |  |
| Msc1 | - |  |
| Nop56 | - | [1] |
| Om45 | - | [1] |
| Pai3 | Inhibitor of Pep4 [19], the major vacuolar protease essential for the degradation of autophagosomal cargoes [20] |  |
| Pdi1 | - | [1] |
| Pmi40 | - |  |
| Prc1 | Vacuolar protease essential for the degradation of autophagosomal cargoes [20] |  |
| Pre9 | Subunit of the 26S proteasome, which is targeted by selective autophagy [17,18,21] | [1] |
| Prp43 | - | [1] |
| Prs5 | - | [1] |
| Prt1 | - | [1] |
| Pub1 | Component of stress granules, which are degraded by autophagy [22] | [1] |
| Rib3 | - |  |
| Rpn2 | Subunit of the 26S proteasome, which is targeted by selective autophagy [17,18,21] | [1] |
| Rpp1a | Subunit of the 60S ribosome, which is selectively degraded by autophagy [23] | [1] |
| Rtc3 | - |  |
| Rtg2 | - |  |
| Sac6 | - | [1,15] |
| Sam4 | - |  |
| Sec4 | Required for Atg9 trafficking and autophagy [9] | [1,15] |
| Sec66 | - | [1] |
| Sey1 | - | [1] |
| Sft1 | Possibly involved in Atg9 trafficking [24] | [15] |
| Sis1 | - | [1] |
| Skp1 | - | [1] |
| Sla1 | Involved in Ede1-mediated selective autophagy of aberrant CME protein condensates [1] | [1] |
| Atg24 | Mediates lipid trafficking promoting autophagy and vacuole membrane fusion [25]; involved in the Cvt pathway [26], proteaphagy [27], possibly mitophagy [28], Atg27 retrograde trafficking/recycling from vacuoles [29,30] and vacuolar targeting of transcription factors controlling *ATG* gene expression [31]; interacts with the Atg1 kinase complex [26] | [1] |
| Ssa2 | Candidate autophagosomal cargo [2] | [1] |
| Ssd1 | Possible positive regulator of autophagy [32] | [1] |
| Sti1 | Candidate autophagosomal cargo [2] |  |
| Sui3 | - | [1] |
| Thr4 | Candidate autophagosomal cargo [2] | [1] |
| Tif35 | - | [1] |
| Tim11 | - | [1] |
| Tpk2 | Catalytic subunit of PKA, which regulates autophagy [33-35] |  |
| Tpm2 | - |  |
| Tsa1 | Cargo of Cue5-mediated aggrephagy [13]; candidate  autophagosomal cargo [2] |  |
| Tub1 | - | [1] |
| Ubc1 | E2 conjugating enzyme involved in autophagic degradation of 26S proteasomes [18] |  |
| Ura10 | - |  |
| Vac8 | Essential to organize the Atg machinery in proximity of the vacuole [36-38]; involved in micronucleophagy [39] | [1] |
| Vma1 | Subunit of the V-ATPase involved in acidification of the vacuolar lumen, which is essential for the degradation of autophagosomal cargoes [40] | [1] |
| Vma13 | Subunit of the V-ATPase involved in acidification of the vacuolar lumen, which is essential for the degradation of autophagosomal cargoes [40] |  |
| Vma4 | Subunit of the V-ATPase involved in acidification of the vacuolar lumen, which is essential for the degradation of autophagosomal cargoes [40] |  |
| Vph1 | Subunit of the V-ATPase involved in acidification of the vacuolar lumen, which is essential for the degradation of autophagosomal cargoes [40]; selectively degraded by ESCRT-dependent microautophagy of the vacuole [41] |  |
| Vps35 | Involved in Atg9 trafficking [42] |  |
| Yak1 | Inhibited by TORC1, involved in TORC1 feedback control circuits [43] | [1] |
| Ybl029c-a | - |  |
| Ydl124w | - |  |
| Ygr127w | - |  |
| Ykt6 | SNARE involved in Atg9 trafficking and autophagosome fusion with vacuoles [44-46] |  |
| Ylr225c | - |  |
| Ynl115c | - |  |
| Ynl134c | Candidate autophagosomal cargo [2] |  |
| Ynl208w | - |  |
| Ypl247c | - |  |
| Enriched interactors (p-value < 0.05) | | |
| **Protein** | **Autophagy-related function(s) in yeast** | **Other MS analyses** |
| Aat2 | Candidate autophagosomal cargo [2] |  |
| Abf1 | - | [1] |
| Abp1 | Cargo of Cue5-mediated aggrephagy [13]; regulates ARP2/3 complex-mediated actin assembly, which is involved in Atg9 trafficking [47] | [1,15] |
| Acb1 | Secretory autophagosome cargo [48]; negative regulator of autophagy [49] | - |
| Ade16 | - | [1] |
| Ade17 | - | [1] |
| Ade6 | - |  |
| Adh1 | Candidate autophagosomal cargo [2]; degraded by autophagy upon zink starvation [50] | [1] |
| Adk1 | - |  |
| Adp1 | - |  |
| Ahp1 | Candidate autophagosomal cargo [2] | [1] |
| Ald2 | - | [1] |
| Ald6 | Selective autophagosomal cargo [51], negative regulator of autophagy which is transcriptionally targeted by Stb5 [52] | [1] |
| Amd1 | - | [1] |
| Anp1 | - |  |
| Ape1 | Cvt pathway cargo [53] | [1,54] |
| Ape2 | - |  |
| Ape4 | Cvt pathway cargo [55] | [54] |
| Ara1 | - |  |
| Arc15 | Part of the Arp2/3 complex, which regulates Atg9 trafficking during selective types of autophagy [47] |  |
| Arg4 | - |  |
| Aro2 | - | [1] |
| Aro7 | - |  |
| Aro8 | - |  |
| Arp3 | Part of the Arp2/3 complex, which regulates Atg9 trafficking during selective types of autophagy [47] | [1] |
| Asc1 | Candidate autophagosomal cargo [2] | [1] |
| Atg3 | Atg machinery core component. E2-like enzyme involved in Atg8 conjugation to PE [56]; directly interacts with Atg8 [57] | [1,54] |
| Atg8 | Atg8 can self-interact [58] | [1,15] |
| Atg27 | Binds to Atg9 and involved in its trafficking [59] |  |
| Atg42 | Vacuolar protease essential for the degradation of autophagosomal cargoes [60] |  |
| Atg46 | Negatively regulates autophagy and transcriptionally targeted by the negative regulator of autophagy Stb5 [52] |  |
| Ayr1 | Triacylglycerol lipase involved in autophagy [61] |  |
| Bcy1 | Negative regulatory subunit of PKA, which negatively regulates autophagy [33,35,62-64] | [1] |
| Bmh1 | Participates in the regulation of *ATG8* transcription [65]; candidate autophagosomal cargo [2] | [1] |
| Bmh2 | Participates in the regulation of *ATG8* transcription [65]; candidate autophagosomal cargo [2] | [1] |
| Bna1 | - | [1] |
| Caf20 | - | [1] |
| Cap2 | - |  |
| Car1 | - |  |
| Cbc2 | - | [1] |
| Ccs1 | - | [1] |
| Cct3 | - | [1] |
| Cct4 | - | [1] |
| Cdc12 | - | [1] |
| Cdc28 | - | [1] |
| Cdc33 | Translation initiation factor that interacts with Psp2 and Dhh1 through Eap1 and Ded1, which positively regulates autophagy by promoting Atg1 and Atg13 translation [66-69] | [1] |
| Cdc39 | Subunit of the Ccr4-Not1 core complex, which regulates mRNA levels of several *ATG* genes [70] | [1] |
| Cdc48 | Binding partner of Atg8 involved in autophagosome formation [71], ribophagy [72], micronucleophagy [71] and granulophagy [22] | [1,15] |
| Cdc60 | Leucyl tRNA synthetase which is a leucine sensor for TORC1 and is involved in its activation [73,74]; candidate autophagosomal cargo [2] | [1] |
| Ckb1 | Regulatory subunit of casein kinase 2, which regulates the function of Atg32 during mitophagy [75] | [1] |
| Ckb2 | Regulatory subunit of casein kinase 2, which regulates the function of Atg32 during mitophagy [75] | [1] |
| Cki1 | Involved in the biosynthesis of phosphatidylcholine, which is important for phagophore closure [76] |  |
| Cmk2 | - | [1] |
| Cmp2 | - | [1] |
| Cog7 | Subunit of the COG complex, which is involved in autophagy [77] |  |
| Coi1 | - |  |
| Cox19 | - |  |
| Cpr1 | Cargo of Cue5-mediated aggrephagy [13] | [1,15] |
| Cpr3 | - |  |
| Csr1 | - |  |
| Cyc3 | - | [1] |
| Cys4 | Candidate autophagosomal cargo [2] | [1] |
| Dak1 | - |  |
| Dcs1 | - |  |
| Ded1 | Regulates *ATG1* expression at the posttranscriptional level [68]; component of stress granules, which are degraded by autophagy [22] | [1] |
| Dhh1 | Promotes Atg1 and Atg13 translation during nitrogen starvation while promoting the degradation of *ATG* transcripts during nutrient-rich condition, regulating autophagy [67,78] | [1] |
| Dph5 | - |  |
| Dtd1 | - |  |
| Dys1 | - |  |
| Ecm33 | Negative regulator of autophagy [79] | [1] |
| Eft1 | Candidate autophagosomal cargo [2] | [1] |
| Eis1 | - | [1] |
| Elg1 | - |  |
| Emi2 | - |  |
| End3 | Involved in Ede1-mediated selective autophagy of aberrant CME protein condensates [1]; required for ER-phagy [80] | [1] |
| Eno1 | - |  |
| Ent5 | Involved in Atg27 trafficking [10] | [1] |
| Erg13 | - | [1] |
| Erg20 | - |  |
| Ero1 | - |  |
| Erv1 | - | [1] |
| Erv25 | - | [1,15] |
| Faa1 | Acyl-CoA synthetase that localizes to forming autophagosomes which is essential for phagophore expansion [81] | [1] |
| Fas1 | Selective autophagosomal cargo [82] | [1,54] |
| Fba1 | Degraded by autophagy upon zink starvation [50] | [1] |
| Fmp10 | - | [1] |
| Fmp40 | - |  |
| Fms1 | - |  |
| Frd1 | - | [1] |
| Frs2 | Candidate autophagosomal cargo [2] | [1] |
| Fum1 | - |  |
| Fun12 | Candidate autophagosomal cargo [2] | [1] |
| Gas1 | - |  |
| Gcd11 | - | [1] |
| Gcs1 | - | [1] |
| Gcy1 | - |  |
| Get3 | - | [1] |
| Get4 | - |  |
| Gfa1 | - | [1] |
| Gga1 | - |  |
| Gln4 | - |  |
| Gos1 | Involved in Atg9 trafficking [83] | [1,15] |
| Gpm1 | Candidate autophagosomal cargo [2] | [1] |
| Gpm2 | - |  |
| Gpd2 | - |  |
| Gre1 | - |  |
| Grh1 | Involved in secretory autophagy [48] |  |
| Grs1 | Candidate autophagosomal cargo [2] | [1] |
| Grx1 | - | [1] |
| Gsp1 | Candidate autophagosomal cargo [2] |  |
| Gtt1 | - |  |
| Hek2 | - |  |
| Hem13 | - |  |
| Hem15 | - |  |
| His3 | - |  |
| Hmf1 | - | [1] |
| Hoc1 | - |  |
| Hom2 | Candidate autophagosomal cargo [2] | [1] |
| Hom6 | Candidate autophagosomal cargo [2] |  |
| Hri1 | - |  |
| Hsp26 | Component of stress granules, which are degraded by autophagy [22] | [1] |
| Hsp31 | Negatively regulates TORC1 and, thus, positively regulates autophagy in response to carbon starvation [84] |  |
| Hsp82 | - |  |
| Hts1 | - |  |
| Hxt7 | Candidate autophagosomal cargo [85] |  |
| Ifa38 | - |  |
| Igo1 | Phosphorylated Igo1 directly inhibits the Cdc55 phosphatase [86], which is required for sufficient Atg13 dephosphorylation and autophagy induction after TORC1 inactivation [87]; required for pre-meiotic autophagy [88] |  |
| Iki3 | - | [1] |
| Ils1 | Candidate autophagosomal cargo [2] | [1] |
| Ino1 | - |  |
| Inp53 | Redundant with Sac1 and Ymr1 in the formation and maturation of autophagosomes, respectively [89,90] |  |
| Kar2 | - | [1] |
| Kex1 | - | [1] |
| Ktr1 | - | [1] |
| Leu1 | Candidate autophagosomal cargo [2] | [1] |
| Lia1 | Candidate autophagosomal cargo [2] |  |
| Log1 | - |  |
| Lys2 | - |  |
| Map1 | - | [1] |
| Map2 | - |  |
| Mbf1 | - |  |
| Mck1 | Acts in parallel to Rim15 to activate starvation-induced gene expression, exit from the mitotic cell cycle and acquisition of G_0_-specific characteristics [91]; together with the rest of GSK-3 kinases in yeast (Mrk1, Rim11 and Ygk3) is involved in the phosphorylation of Elo2 regulating very long chain fatty acid synthesis and autophagy [92]. |  |
| Mdh2 | - |  |
| Ego1 | Regulates autophagy via TORC1 [93,94]; involved in microautophagy regulation [95] | [1] |
| Mes1 | - | [1] |
| Mic26 | - | [1] |
| Mnn1 | - | [1] |
| Mpm1 | - | [1] |
| Mrn1 | - | [1] |
| Mrpl6 | - |  |
| Mrt4 | - |  |
| Mrx1 | - |  |
| Nap1 | - | [1] |
| Nde1 | - | [1] |
| Nit3 | - |  |
| Nnr2 | - |  |
| Nop58 | - | [1] |
| Nop9 | - |  |
| Npt1 | - |  |
| Osh6 | Involved in piecemeal microautophagy of the nucleus [96] | [1] |
| Paa1 | - | [1] |
| Pab1 | Component of stress granules, which are degraded by autophagy [22]; candidate autophagosomal cargo [2] | [1] |
| Pdx3 | - |  |
| Pep4 | Major vacuolar protease essential for the degradation of autophagosomal cargoes [20] |  |
| Pet191 | - |  |
| Pet9 | - | [1] |
| Pex19 | - | [1] |
| Pfk2 | Selective autophagosomal cargo [2] | [1] |
| Pgi1 | Candidate autophagosomal cargo [2] | [1] |
| Pgm1 | - |  |
| Pgm2 | - |  |
| Phb1 | - | [1] |
| Pho88 | - | [1] |
| Pil1 | Cargo of Cue5-mediated aggrephagy [13]; positive regulator of mitophagy and autophagy [97] | [1,15] |
| Pma1 | - |  |
| Pmc1 | - | [1] |
| Pmt2 | - | [1] |
| Pob3 | - | [1] |
| Pom33 | Transmembrane subunit of the nuclear pore complex, selectively degraded by autophagy [98] |  |
| Phm5 | - | [1] |
| Pre10 | Subunit of the 26S proteasome, which is targeted by selective autophagy [17,18,21] | [1] |
| Pre3 | Subunit of the 26S proteasome, which is targeted by selective autophagy [17,18,21] |  |
| Pre5 | Subunit of the 26S proteasome, which is targeted by selective autophagy [17,18,21] | [1] |
| Pre6 | Subunit of the 26S proteasome, which is targeted by selective autophagy [17,18,21] | [1] |
| Pre7 | Subunit of the 26S proteasome, which is targeted by selective autophagy [17,18,21] | [1] |
| Pro3 | - | [1] |
| Psa1 | - | [1] |
| Ptc7 | - |  |
| Ptk2 | - | [1] |
| Pup2 | Subunit of the 26S proteasome, which is targeted by selective autophagy [17,18,21] | [1] |
| Pyc2 | - |  |
| Ras2 | Autophagy regulator [99] |  |
| Ret2 | - | [1] |
| Rim1 | - | [1] |
| Rli1 | - |  |
| Rna1 | - | [1] |
| Rpb2 | - | [1] |
| Rpl14a; Rpl14b | Subunit of the 60S ribosome, which is selectively degraded by autophagy [23] |  |
| Rpl16a | Subunit of the 60S ribosome, which is selectively degraded by autophagy [23]; candidate autophagosomal cargo [2] | [1] |
| Rpl24a | Subunit of the 60S ribosome, which is selectively degraded by autophagy [23] | [1] |
| Rpl26b | Subunit of the 60S ribosome, which is selectively degraded by autophagy [23]; cargo of Cue5-mediated aggrephagy [13]; candidate autophagosomal cargo [2] | [1] |
| Rpl27a | Subunit of the 60S ribosome, which is selectively degraded by autophagy [23]; candidate autophagosomal cargo [2] |  |
| Rpl3 | Subunit of the 60S ribosome, which is selectively degraded by autophagy [23]; candidate autophagosomal cargo [2] | [1] |
| Rpl31b | Subunit of the 60S ribosome, which is selectively degraded by autophagy [23]; candidate autophagosomal cargo [2] | [1] |
| Rpl6b | Subunit of the 60S ribosome, which is selectively degraded by autophagy [23] | [1] |
| Rpl7b | Subunit of the 60S ribosome, which is selectively degraded by autophagy [23] | [1] |
| Rpl8a | Subunit of the 60S ribosome, which is selectively degraded by autophagy [23] | [1] |
| Rpn12 | Subunit of the 26S proteasome, which is targeted by selective autophagy [17,18,21] | [1] |
| Rpn6 | Subunit of the 26S proteasome, which is targeted by selective autophagy [17,18,21] | [1] |
| Rpp0 | Subunit of the 60S ribosome, which is selectively degraded by autophagy [23]; candidate autophagosomal cargo [2] |  |
| Rps21b | Candidate autophagosomal cargo [2] | [1] |
| Rps23A; Rps23B | - |  |
| Rps31 | - | [1] |
| Rpt2 | Subunit of the 26S proteasome, which is targeted by selective autophagy [17,18,21] | [1] |
| Rpt4 | Subunit of the 26S proteasome, which is targeted by selective autophagy [17,18,21] | [1] |
| Rpt6 | Subunit of the 26S proteasome, which is targeted by selective autophagy [17,18,21] | [1] |
| Rsp5 | Ubiquitin ligase involved in the selective autophagy of aggregates, proteasomes, mitochondria and possibly ribosomes [13,18,100,101]; involved in microautophagy of vacuolar membrane proteins and proteasomes [41,102] | [1] |
| Rtn1 | - | [1] |
| Rtn2 | - | [1] |
| Rvb2 | Helicase subunit of the Ino80-chromatin remodeling complex, which is involved in the transcriptional repression of *ATG* genes [103] | [1] |
| Rvs161 | - | [1] |
| Sah1 | Candidate autophagosomal cargo [2]; involved in phosphatidylcholine biosynthesis, which is important for phagophore closure [76] | [1] |
| Sba1 | - |  |
| Scd6 | Component of stress granules, which are degraded by autophagy [22] | [1] |
| Sch9 | Cooperatively regulates autophagy induction with PKA [35] | [1] |
| Sco1 | - |  |
| Scp160 | - |  |
| Scs2 | Involved in ER-phagy [80] | [1] |
| Sec26 | - | [1,15] |
| Sec28 | - | [1] |
| Sec31 | Subunit of COPII vesicles, which are a membrane source for autophagosome biogenesis [15,104,105] and are involved in Atg9 sorting out of the ER [106] | [1] |
| Sec53 | Candidate autophagosomal cargo [2] | [1] |
| Sfa1 | - |  |
| Shm2 | - | [1] |
| Slm1 | - | [1] |
| Sna4 | Vacuolar protein degraded by microautophagy [107] |  |
| Snu13 | - | [1] |
| Snx41 | Cooperates with Sxn4 and Snx42 to mediate proteasome turnover [27]; involved in Atg27 trafficking together with Snx4 [29] | [1] |
| Sop4 | - |  |
| Spe3 | - |  |
| Srp1 | - | [1] |
| Srx1 | - |  |
| Ssa4 | - | [1] |
| Ssb1 | Candidate autophagosomal cargo [2] | [1] |
| Sse1 | ­Candidate autophagosomal cargo [2] | [1] |
| Sso1 | Required for Atg9 trafficking and autophagy [46]; required for autophagosome-mediated unconventional protein secretion [48] | [1,15] |
| Sss1 | - | [1] |
| Ssz1 | Candidate autophagosomal cargo [2] | [1] |
| Ste23 | - | [1] |
| Stm1 | Upon autophagy induction, acts as a 80S ribosome preservation factor [108] | [1] |
| Syp1 | Involved in Ede1-mediated selective autophagy of aberrant CME protein condensates [1] |  |
| Tal1 | - |  |
| Tcp1 | - | [1] |
| Tdh1 | - | [1] |
| Thr1 | - |  |
| Ths1 | - | [1] |
| Tif3 | - | [1] |
| Tma19 | Cargo of Cue5-mediated aggrephagy [13]; candidate autophagosomal cargo [2]; negative regulator of autophagy [109] | [1,15] |
| Tps3 | - | [1] |
| Tsl1 | - | [1] |
| Tsr1 | - | [1] |
| Tup1 | - |  |
| Ty1B-mr1; Ty1B-bl | Cvt pathway cargo[110] | [1] |
| Ty1b-pr2; Ty1b-ml1 | Cvt pathway cargo [110] |  |
| Ty2b-c | Cvt pathway cargo [110] |  |
| Uba1 | Candidate autophagosomal cargo [2] |  |
| Ubc7 | - |  |
| Ufd4 | - | [1] |
| Ura2 | - | [1] |
| Ura3 | - |  |
| Utr4 | - |  |
| Vam10 | - |  |
| Vam3 | SNARE involved in the fusion of autophagosomes with the vacuole [111]; Atg8 binding partner [112] |  |
| Vas1 | - | [1] |
| Vma10 | Subunit of the V-ATPase involved in acidification of the vacuolar lumen, which is essential for the degradation of autophagosomal cargoes [40] |  |
| Vma2 | Subunit of the V-ATPase involved in acidification of the vacuolar lumen, which is essential for the degradation of autophagosomal cargoes [40] | [1,15] |
| Vma5 | Subunit of the V-ATPase involved in acidification of the vacuolar lumen, which is essential for the degradation of autophagosomal cargoes [40]; Candidate autophagosomal cargo [2] |  |
| Vma6 | Subunit of the V-ATPase involved in acidification of the vacuolar lumen, which is essential for the degradation of autophagosomal cargoes**) [40]** |  |
| Vps1 | Involved in Atg9 trafficking [113]; involved in pexophagy [11] | [1,15] |
| Vps21 | Regulates phagophore closure [114-116] | [15] |
| Vps24 | ESCRT-III component possibly involved in autophagosome closure [116], microautophagy [107] and secretory autophagy [117] |  |
| Vps74 | Regulates the function of Sac1 phosphatase [118], which restrains phosphatidylinositol-4-phosphate incorporation into Atg9 vesicles [119] |  |
| Vtc4 | Subunit of the vacuolar transporter chaperone complex, which is required for microautophagy [120] | [1] |
| Whi2 | Involved in amino acid sensing and negatively regulating TORC1 [121]; required for induction of mitophagy [122] | [1] |
| Wwm1 | - | [1] |
| Yck2 | - | [1] |
| Yck3 | - | [1,15] |
| Ydl086W | - |  |
| Ydr341c | - |  |
| Yet3 | - | [1] |
| Yfr006w | - |  |
| Ygl039w | - |  |
| Yhm2 | - | [1] |
| Yhr020w | Candidate autophagosomal cargo [2] | [1] |
| Ynr021w | - |  |
| Yol057w | - |  |
| Ypr1 | - |  |
| Ypr127w | - |  |
| Ypt1 | Essential for autophagy progression [8] ; recruited to the PAS by Atg9 vesicles and the TRAPPIII complex [8,123] | [15] |
| Ypt31 | Important for autophagy progression [124] | [15] |
| Zta1 | - |  |

**References**

1. Wilfling F, Lee CW, Erdmann PS, et al. A Selective Autophagy Pathway for Phase-Separated Endocytic Protein Deposits. Mol Cell. 2020 Dec 3;80(5):764-778 e7.

2. Suzuki K, Nakamura S, Morimoto M, et al. Proteomic profiling of autophagosome cargo in Saccharomyces cerevisiae. PLoS One. 2014;9(3):e91651.

3. Shintani T, Suzuki K, Kamada Y, et al. Apg2p functions in autophagosome formation on the perivacuolar structure. J Biol Chem. 2001 Aug 10;276(32):30452-60.

4. Wang CW, Kim J, Huang WP, et al. Apg2 is a novel protein required for the cytoplasm to vacuole targeting, autophagy, and pexophagy pathways. J Biol Chem. 2001 Aug 10;276(32):30442-51.

5. Harada K, Kotani T, Kirisako H, et al. Two distinct mechanisms target the autophagy-related E3 complex to the pre-autophagosomal structure. Elife. 2019 Feb 27;8.

6. Juris L, Montino M, Rube P, et al. PI3P binding by Atg21 organises Atg8 lipidation. EMBO J. 2015 Apr 1;34(7):955-73.

7. Rossi G, Yu Ja Fau - Newman AP, Newman Ap Fau - Ferro-Novick S, et al. Dependence of Ypt1 and Sec4 membrane attachment on Bet2. Nature. 1991;351(6322)(0028-0836 (Print)):158-161.

8. Lynch-Day MA, Bhandari D, Menon S, et al. Trs85 directs a Ypt1 GEF, TRAPPIII, to the phagophore to promote autophagy. Proc Natl Acad Sci U S A. 2010 Apr 27;107(17):7811-6.

9. Geng J, Nair U, Yasumura-Yorimitsu K, et al. Post-Golgi Sec Proteins Are Required for Autophagy in *Saccharomyces cerevisiae*. Molecular Biology of the Cell. 2010;21(13):2257-2269.

10. Segarra VA, Sharma A, Lemmon SK. Atg27p localization is clathrin- and Ent3p/5p-dependent. MicroPubl Biol. 2021 Published 2021 Mar 29.(2578-9430 (Electronic)).

11. Mao K, Liu X, Feng Y, et al. The progression of peroxisomal degradation through autophagy requires peroxisomal division. Autophagy. 2014 Apr;10(4):652-61.

12. Mao K, Wang K, Liu X, et al. The scaffold protein Atg11 recruits fission machinery to drive selective mitochondria degradation by autophagy. Dev Cell. 2013 Jul 15;26(1):9-18.

13. Lu K, Psakhye I, Jentsch S. Autophagic clearance of polyQ proteins mediated by ubiquitin-Atg8 adaptors of the conserved CUET protein family. Cell. 2014 Jul 31;158(3):549-63.

14. Lebesgue N, Megyeri M, Cristobal A, et al. Combining Deep Sequencing, Proteomics, Phosphoproteomics, and Functional Screens To Discover Novel Regulators of Sphingolipid Homeostasis. J Proteome Res. 2017 Feb 3;16(2):571-582.

15. Graef M, Friedman JR, Graham C, et al. ER exit sites are physical and functional core autophagosome biogenesis components. Mol Biol Cell. 2013 Sep;24(18):2918-31.

16. Morshed S, Sharmin T, Ushimaru T. TORC1 regulates ESCRT-0 complex formation on the vacuolar membrane and microautophagy induction in yeast. Biochem Biophys Res Commun. 2020 Jan 29;522(1):88-94.

17. Marshall RS, McLoughlin F, Vierstra RD. Autophagic Turnover of Inactive 26S Proteasomes in Yeast Is Directed by the Ubiquitin Receptor Cue5 and the Hsp42 Chaperone. Cell Rep. 2016 Aug 9;16(6):1717-1732.

18. Marshall RS, Vierstra RD. A trio of ubiquitin ligases sequentially drives ubiquitylation and autophagic degradation of dysfunctional yeast proteasomes. Cell Rep. 2022 Mar 15;38(11):110535.

19. Schu P, Wolf DH. The proteinase yscA-inhibitor, IA3, gene. Studies of cytoplasmic proteinase inhibitor deficiency on yeast physiology. FEBS Lett. 1991 May 20;283(1):78-84.

20. Takeshige K, Baba M, Tsuboi S, et al. Autophagy in yeast demonstrated with proteinase-deficient mutants and conditions for its induction. Journal of Cell Biology. 1992;119(2):301-311.

21. Waite KA, De-La Mota-Peynado A, Vontz G, et al. Starvation Induces Proteasome Autophagy with Different Pathways for Core and Regulatory Particles. J Biol Chem. 2016 Feb 12;291(7):3239-53.

22. Buchan JR, Kolaitis RM, Taylor JP, et al. Eukaryotic stress granules are cleared by autophagy and Cdc48/VCP function. Cell. 2013 Jun 20;153(7):1461-74.

23. Kraft C, Deplazes A, Sohrmann M, et al. Mature ribosomes are selectively degraded upon starvation by an autophagy pathway requiring the Ubp3p/Bre5p ubiquitin protease. Nat Cell Biol. 2008 May;10(5):602-10.

24. Zou S, Sun D, Liang Y. The Roles of the SNARE Protein Sed5 in Autophagy in Saccharomyces cerevisiae. Mol Cells. 2017 Sep 30;40(9):643-654.

25. Ma M, Kumar S, Purushothaman L, et al. Lipid trafficking by yeast Snx4 family SNX-BAR proteins promotes autophagy and vacuole membrane fusion. Mol Biol Cell. 2018 Sep 1;29(18):2190-2200.

26. Nice DC, Sato TK, Stromhaug PE, et al. Cooperative binding of the cytoplasm to vacuole targeting pathway proteins, Cvt13 and Cvt20, to phosphatidylinositol 3-phosphate at the pre-autophagosomal structure is required for selective autophagy. J Biol Chem. 2002 Aug 16;277(33):30198-207.

27. Nemec AA, Howell LA, Peterson AK, et al. Autophagic clearance of proteasomes in yeast requires the conserved sorting nexin Snx4. J Biol Chem. 2017 Dec 29;292(52):21466-21480.

28. Okamoto K, Kondo-Okamoto N, Ohsumi Y. Mitochondria-anchored receptor Atg32 mediates degradation of mitochondria via selective autophagy [Research Support, Non-U.S. Gov't]. Dev Cell. 2009 Jul;17(1):87-97.

29. Ma M, Burd CG, Chi RJ. Distinct complexes of yeast Snx4 family SNX-BARs mediate retrograde trafficking of Snc1 and Atg27. Traffic. 2017 Feb;18(2):134-144.

30. Suzuki SW, Emr SD. Retrograde trafficking from the vacuole/lysosome membrane. Autophagy. 2018;14(9):1654-1655.

31. Hanley SE, Willis SD, Cooper KF. Snx4-assisted vacuolar targeting of transcription factors defines a new autophagy pathway for controlling ATG expression. Autophagy. 2021 Nov;17(11):3547-3565.

32. Kramer MH, Farre JC, Mitra K, et al. Active Interaction Mapping Reveals the Hierarchical Organization of Autophagy. Mol Cell. 2017 Feb 16;65(4):761-774 e5.

33. Stephan JS, Yeh Y-Y, Ramachandran V, et al. The Tor and PKA signaling pathways independently target the Atg1/Atg13 protein kinase complex to control autophagy. Proceedings of the National Academy of Sciences. 2009;106(40):17049-17054.

34. Yu Q, Gong X, Tong Y, et al. Phosphorylation of Jhd2 by the Ras-cAMP-PKA(Tpk2) pathway regulates histone modifications and autophagy. Nat Commun. 2022 Sep 27;13(1):5675.

35. Yorimitsu T, Zaman S, Broach JR, et al. Protein kinase A and Sch9 cooperatively regulate induction of autophagy in Saccharomyces cerevisiae. Mol Biol Cell. 2007 Oct;18(10):4180-9.

36. Hollenstein DM, Licheva M, Konradi N, et al. Spatial control of avidity regulates initiation and progression of selective autophagy. Nat Commun. 2021 Dec 10;12(1):7194.

37. Hollenstein DM, Gomez-Sanchez R, Ciftci A, et al. Vac8 spatially confines autophagosome formation at the vacuole in S. cerevisiae. J Cell Sci. 2019 Nov 14;132(22).

38. Gatica D, Wen X, Cheong H, et al. Vac8 determines phagophore assembly site vacuolar localization during nitrogen starvation-induced autophagy. Autophagy. 2021 Jul;17(7):1636-1648.

39. Roberts P, Moshitch-Moshkovitz S, Kvam E, et al. Piecemeal microautophagy of nucleus in *Saccharomyces cerevisiae* [Research Support, U.S. Gov't, Non-P.H.S.

Research Support, U.S. Gov't, P.H.S.]. Mol Biol Cell. 2003 Jan;14(1):129-41.

40. Nakamura N, Matsuura A, Wada Y, et al. Acidification of Vacuoles Is Required for Autophagic Degradation in the Yeast, *Saccharomyces cerevisiae*. The Journal of Biochemistry. 1997;121(2):338-344.

41. Yang X, Zhang W, Wen X, et al. TORC1 regulates vacuole membrane composition through ubiquitin- and ESCRT-dependent microautophagy. J Cell Biol. 2020 Mar 2;219(3).

42. Marquardt L, Taylor M, Kramer F, et al. Vacuole fragmentation depends on a novel Atg18-containing retromer-complex. Autophagy. 2023 Jan;19(1):278-295.

43. Dokladal L, Stumpe M, Hu Z, et al. Phosphoproteomic responses of TORC1 target kinases reveal discrete and convergent mechanisms that orchestrate the quiescence program in yeast. Cell Rep. 2021 Dec 28;37(13):110149.

44. Gao J, Reggiori F, Ungermann C. A novel in vitro assay reveals SNARE topology and the role of Ykt6 in autophagosome fusion with vacuoles. J Cell Biol. 2018 Oct 1;217(10):3670-3682.

45. Bas L, Papinski D, Licheva M, et al. Reconstitution reveals Ykt6 as the autophagosomal SNARE in autophagosome-vacuole fusion. J Cell Biol. 2018 Oct 1;217(10):3656-3669.

46. Nair U, Jotwani A, Geng J, et al. SNARE proteins are required for macroautophagy. Cell. 2011 Jul 22;146(2):290-302.

47. Monastyrska I, He C, Geng J, et al. Arp2 Links Autophagic Machinery with the Actin Cytoskeleton. Molecular Biology of the Cell. 2008;19(5):1962-1975.

48. Duran JM, Anjard C, Stefan C, et al. Unconventional secretion of Acb1 is mediated by autophagosomes. J Cell Biol. 2010 Feb 22;188(4):527-36.

49. Montegut L, Joseph A, Chen H, et al. DBI/ACBP is a targetable autophagy checkpoint involved in aging and cardiovascular disease. Autophagy. 2023 Jul;19(7):2166-2169.

50. Kawamata T, Horie T, Matsunami M, et al. Zinc starvation induces autophagy in yeast. J Biol Chem. 2017 May 19;292(20):8520-8530.

51. Onodera J, Ohsumi Y. Ald6p is a preferred target for autophagy in yeast, *Saccharomyces cerevisiae*. J Biol Chem. 2004 Apr 16;279(16):16071-6.

52. Delorme-Axford E, Wen X, Klionsky DJ. The yeast transcription factor Stb5 acts as a negative regulator of autophagy by modulating cellular metabolism. Autophagy. 2023 Jul 2:1-14.

53. Klionsky DJ, Cueva R, Yaver DS. Aminopeptidase I of Saccharomyces cerevisiae is localized to the vacuole independent of the secretory pathway. Journal of Cell Biology. 1992;119(2):287-299.

54. Tomioka Y, Kotani T, Kirisako H, et al. TORC1 inactivation stimulates autophagy of nucleoporin and nuclear pore complexes. J Cell Biol. 2020 Jul 6;219(7).

55. Yuga M, Gomi K, Klionsky DJ, et al. Aspartyl Aminopeptidase Is Imported from the Cytoplasm to the Vacuole by Selective Autophagy in Saccharomyces cerevisiae. Journal of Biological Chemistry. 2011;286(15):13704-13713.

56. Ichimura Y, Kirisako T, Takao T, et al. A ubiquitin-like system mediates protein lipidation. Nature. 2000;408(6811):488-492.

57. Yamaguchi M, Noda NN, Nakatogawa H, et al. Autophagy-related protein 8 (Atg8) family interacting motif in Atg3 mediates the Atg3-Atg8 interaction and is crucial for the cytoplasm-to-vacuole targeting pathway. J Biol Chem. 2010 Sep 17;285(38):29599-607.

58. Nakatogawa H, Ichimura Y, Ohsumi Y. Atg8, a ubiquitin-like protein required for autophagosome formation, mediates membrane tethering and hemifusion. Cell. 2007 Jul 13;130(1):165-78.

59. Legakis JE, Yen W-L, Klionsky DJ. A Cycling Protein Complex Required for Selective Autophagy. Autophagy. 2007;3(5):422-432.

60. Parzych KR, Ariosa A, Mari M, et al. A newly characterized vacuolar serine carboxypeptidase, Atg42/Ybr139w, is required for normal vacuole function and the terminal steps of autophagy in the yeast Saccharomyces cerevisiae. Mol Biol Cell. 2018 May 1;29(9):1089-1099.

61. Shpilka T, Welter E, Borovsky N, et al. Lipid droplets and their component triglycerides and steryl esters regulate autophagosome biogenesis. EMBO J. 2015 Aug 13;34(16):2117-31.

62. Johnson KE, Cameron S, Toda T, et al. Expression in Escherichia coli of BCY1, the regulatory subunit of cyclic AMP-dependent protein kinase from Saccharomyces cerevisiae. Purification and characterization. Journal of Biological Chemistry. 1987;262(18):8636-8642.

63. Soulard A, Cremonesi A, Moes S, et al. The rapamycin-sensitive phosphoproteome reveals that TOR controls protein kinase A toward some but not all substrates. Mol Biol Cell. 2010 Oct 1;21(19):3475-86.

64. Schmelzle T, Beck T, Martin DE, et al. Activation of the RAS/cyclic AMP pathway suppresses a TOR deficiency in yeast. Mol Cell Biol. 2004 Jan;24(1):338-51.

65. Kim B, Lee Y, Choi H, et al. The trehalose-6-phosphate phosphatase Tps2 regulates ATG8 transcription and autophagy in Saccharomyces cerevisiae. Autophagy. 2021 Apr;17(4):1013-1027.

66. Yin Z, Liu X, Ariosa A, et al. Psp2, a novel regulator of autophagy that promotes autophagy-related protein translation. Cell Res. 2019 Dec;29(12):994-1008.

67. Liu X, Yao Z, Jin M, et al. Dhh1 promotes autophagy-related protein translation during nitrogen starvation. PLoS Biol. 2019 Apr;17(4):e3000219.

68. Lahiri V, Metur SP, Hu Z, et al. Post-transcriptional regulation of ATG1 is a critical node that modulates autophagy during distinct nutrient stresses. Autophagy. 2022 Jul;18(7):1694-1714.

69. Gulay S, Gupta N, Lorsch JR, et al. Distinct interactions of eIF4A and eIF4E with RNA helicase Ded1 stimulate translation in vivo. Elife. 2020 May 29;9.

70. Yin Z, Zhang Z, Lei Y, et al. Bidirectional roles of the Ccr4-Not complex in regulating autophagy before and after nitrogen starvation. Autophagy. 2023 Feb;19(2):415-425.

71. Krick R, Bremer S, Welter E, et al. Cdc48/p97 and Shp1/p47 regulate autophagosome biogenesis in concert with ubiquitin-like Atg8. J Cell Biol. 2010 Sep 20;190(6):965-73.

72. Ossareh-Nazari B, Bonizec M, Cohen M, et al. Cdc48 and Ufd3, new partners of the ubiquitin protease Ubp3, are required for ribophagy. EMBO Rep. 2010 Jul;11(7):548-54.

73. Duran RV, Hall MN. Leucyl-tRNA synthetase: double duty in amino acid sensing. Cell Res. 2012 Aug;22(8):1207-9.

74. Han JM, Jeong SJ, Park MC, et al. Leucyl-tRNA synthetase is an intracellular leucine sensor for the mTORC1-signaling pathway. Cell. 2012 Apr 13;149(2):410-24.

75. Kanki T, Kurihara Y, Jin X, et al. Casein kinase 2 is essential for mitophagy. EMBO Rep. 2013 Sep;14(9):788-94.

76. Polyansky A, Shatz O, Fraiberg M, et al. Phospholipid imbalance impairs autophagosome completion. EMBO J. 2022 Dec 1;41(23):e110771.

77. Yen WL, Shintani T, Nair U, et al. The conserved oligomeric Golgi complex is involved in double-membrane vesicle formation during autophagy. J Cell Biol. 2010 Jan 11;188(1):101-14.

78. Hu G, McQuiston T, Bernard A, et al. A conserved mechanism of TOR-dependent RCK-mediated mRNA degradation regulates autophagy. Nat Cell Biol. 2015 Jul;17(7):930-942.

79. Umekawa M, Ujihara M, Nakai D, et al. Ecm33 is a novel factor involved in efficient glucose uptake for nutrition-responsive TORC1 signaling in yeast. FEBS Lett. 2017 Nov;591(22):3721-3729.

80. Liu D, Mari M, Li X, et al. ER-phagy requires the assembly of actin at sites of contact between the cortical ER and endocytic pits. Proc Natl Acad Sci U S A. 2022 Feb 8;119(6).

81. Schutter M, Giavalisco P, Brodesser S, et al. Local Fatty Acid Channeling into Phospholipid Synthesis Drives Phagophore Expansion during Autophagy. Cell. 2020 Jan 9;180(1):135-149 e14.

82. Shpilka T, Welter E, Borovsky N, et al. Fatty acid synthase is preferentially degraded by autophagy upon nitrogen starvation in yeast. Proc Natl Acad Sci U S A. 2015 Feb 3;112(5):1434-9.

83. Ohashi Y, Munro S. Membrane delivery to the yeast autophagosome from the Golgi-endosomal system. Mol Biol Cell. 2010 Nov 15;21(22):3998-4008.

84. Miller-Fleming L, Antas P, Pais TF, et al. Yeast DJ-1 superfamily members are required for diauxic-shift reprogramming and cell survival in stationary phase. Proceedings of the National Academy of Sciences. 2014;111(19):7012-7017.

85. Krampe S, Boles E. Starvation-induced degradation of yeast hexose transporter Hxt7p is dependent on endocytosis, autophagy and the terminal sequences of the permease. FEBS Lett. 2002 Feb 27;513(2-3):193-6.

86. Bontron S, Jaquenoud M, Vaga S, et al. Yeast endosulfines control entry into quiescence and chronological life span by inhibiting protein phosphatase 2A. Cell Rep. 2013 Jan 31;3(1):16-22.

87. Yeasmin AM, Waliullah TM, Kondo A, et al. Orchestrated Action of PP2A Antagonizes Atg13 Phosphorylation and Promotes Autophagy after the Inactivation of TORC1. PLoS One. 2016;11(12):e0166636.

88. Sarkar S, Dalgaard JZ, Millar JB, et al. The Rim15-endosulfine-PP2ACdc55 signalling module regulates entry into gametogenesis and quiescence via distinct mechanisms in budding yeast. PLoS Genet. 2014 Jun;10(6):e1004456.

89. Muramoto M, Yamakuchi Y, Konishi R, et al. Essential roles of phosphatidylinositol 4-phosphate phosphatases Sac1p and Sjl3p in yeast autophagosome formation. Biochim Biophys Acta Mol Cell Biol Lipids. 2022 Sep;1867(9):159184.

90. Cebollero E, van der Vaart A, Zhao M, et al. Phosphatidylinositol-3-phosphate clearance plays a key role in autophagosome completion. Curr Biol. 2012 Sep 11;22(17):1545-53.

91. Quan Z, Cao L, Tang Y, et al. The Yeast GSK-3 Homologue Mck1 Is a Key Controller of Quiescence Entry and Chronological Lifespan. PLOS Genetics. 2015;11(6):e1005282.

92. Zimmermann C, Santos A, Gable K, et al. TORC1 inhibits GSK3-mediated Elo2 phosphorylation to regulate very long chain fatty acid synthesis and autophagy. Cell Rep. 2013 Nov 27;5(4):1036-46.

93. Kira S, Kumano Y, Ukai H, et al. Dynamic relocation of the TORC1-Gtr1/2-Ego1/2/3 complex is regulated by Gtr1 and Gtr2. Mol Biol Cell. 2016 Jan 15;27(2):382-96.

94. Varlakhanova NV, Mihalevic MJ, Bernstein KA, et al. Pib2 and the EGO complex are both required for activation of TORC1. J Cell Sci. 2017 Nov 15;130(22):3878-3890.

95. Dubouloz F, Deloche O, Wanke V, et al. The TOR and EGO protein complexes orchestrate microautophagy in yeast. Mol Cell. 2005 Jul 1;19(1):15-26.

96. Kvam E, Goldfarb DS. Nvj1p is the outer-nuclear-membrane receptor for oxysterol-binding protein homolog Osh1p in Saccharomyces cerevisiae. J Cell Sci. 2004 Oct 1;117(Pt 21):4959-68.

97. Pal A, Paripati AK, Deolal P, et al. Eisosome protein Pil1 regulates mitochondrial morphology, mitophagy, and cell death in Saccharomyces cerevisiae. J Biol Chem. 2022 Nov;298(11):102533.

98. Lee C-W, Wilfling F, Ronchi P, et al. Selective autophagy degrades nuclear pore complexes. Nature Cell Biology. 2020;22(2):159-166.

99. Budovskaya YV, Stephan JS, Reggiori F, et al. The Ras/cAMP-dependent protein kinase signaling pathway regulates an early step of the autophagy process in Saccharomyces cerevisiae. J Biol Chem. 2004 May 14;279(20):20663-71.

100. Belgareh-Touze N, Cavellini L, Cohen MM. Ubiquitination of ERMES components by the E3 ligase Rsp5 is involved in mitophagy. Autophagy. 2017 Jan 2;13(1):114-132.

101. Kraft C, Peter M. Is the Rsp5 ubiquitin ligase involved in the regulation of ribophagy? Autophagy. 2008 Aug;4(6):838-40.

102. Li J, Hochstrasser M. Selective microautophagy of proteasomes is initiated by ESCRT-0 and is promoted by proteasome ubiquitylation. J Cell Sci. 2022 Feb 15;135(4).

103. Li XA-O, Mei Q, Yu Q, et al. The TORC1 activates Rpd3L complex to deacetylate Ino80 and H2A.Z and repress autophagy. Sci Adv. 2023;9(2375-2548 (Electronic)):eade8312.

104. Suzuki K, Kubota Y, Sekito T, et al. Hierarchy of Atg proteins in pre-autophagosomal structure organization. Genes Cells. 2007 Feb;12(2):209-18.

105. Tan D, Cai Y, Wang J, et al. The EM structure of the TRAPPIII complex leads to the identification of a requirement for COPII vesicles on the macroautophagy pathway. Proc Natl Acad Sci U S A. 2013 Nov 26;110(48):19432-7.

106. Mari M, Griffith J, Rieter E, et al. An Atg9-containing compartment that functions in the early steps of autophagosome biogenesis. J Cell Biol. 2010 Sep 20;190(6):1005-22.

107. Morshed S, Tasnin MN, Ushimaru T. ESCRT machinery plays a role in microautophagy in yeast. BMC Mol Cell Biol. 2020 Oct 7;21(1):70.

108. Van Dyke N, Chanchorn E, Van Dyke MW. The Saccharomyces cerevisiae protein Stm1p facilitates ribosome preservation during quiescence. Biochem Biophys Res Commun. 2013 Jan 11;430(2):745-50.

109. Vojtova J, Hasek J. Mmi1, the Yeast Ortholog of Mammalian Translationally Controlled Tumor Protein (TCTP), Negatively Affects Rapamycin-Induced Autophagy in Post-Diauxic Growth Phase. Cells. 2020 Jan 7;9(1).

110. Suzuki K, Morimoto M, Kondo C, et al. Selective autophagy regulates insertional mutagenesis by the Ty1 retrotransposon in Saccharomyces cerevisiae. Dev Cell. 2011 Aug 16;21(2):358-65.

111. Darsow T, Rieder SE, Emr SD. A Multispecificity Syntaxin Homologue, Vam3p, Essential for Autophagic and Biosynthetic Protein Transport to the Vacuole. Journal of Cell Biology. 1997;138(3):517-529.

112. Legesse-Miller A, Sagiv Y, Glozman R, et al. Aut7p, a Soluble Autophagic Factor, Participates in Multiple Membrane Trafficking Processes. Journal of Biological Chemistry. 2000;275(42):32966-32973.

113. Arlt H, Raman B, Filali-Mouncef Y, et al. The dynamin Vps1 mediates Atg9 transport to the sites of autophagosome formation. J Biol Chem. 2023 May;299(5):104712.

114. Chen Y, Zhou F, Zou S, et al. A Vps21 endocytic module regulates autophagy. Mol Biol Cell. 2014 Oct 15;25(20):3166-77.

115. Zhou F, Zou S, Chen Y, et al. A Rab5 GTPase module is important for autophagosome closure. PLoS Genet. 2017 Sep;13(9):e1007020.

116. Zhou F, Wu Z, Zhao M, et al. Rab5-dependent autophagosome closure by ESCRT. J Cell Biol. 2019 Jun 3;218(6):1908-1927.

117. Bruns C, McCaffery JM, Curwin AJ, et al. Biogenesis of a novel compartment for autophagosome-mediated unconventional protein secretion. J Cell Biol. 2011 Dec 12;195(6):979-92.

118. Wood CS, Hung CS, Huoh YS, et al. Local control of phosphatidylinositol 4-phosphate signaling in the Golgi apparatus by Vps74 and Sac1 phosphoinositide phosphatase. Mol Biol Cell. 2012 Jul;23(13):2527-36.

119. Zhang H, Zhou J, Xiao P, et al. PtdIns4P restriction by hydrolase SAC1 decides specific fusion of autophagosomes with lysosomes. Autophagy. 2021 Aug;17(8):1907-1917.

120. Uttenweiler A, Schwarz H, Neumann H, et al. The vacuolar transporter chaperone (VTC) complex is required for microautophagy. Mol Biol Cell. 2007 Jan;18(1):166-75.

121. Chen X, Wang G, Zhang Y, et al. Whi2 is a conserved negative regulator of TORC1 in response to low amino acids. PLoS Genet. 2018 Aug;14(8):e1007592.

122. Mendl N, Occhipinti A, Muller M, et al. Mitophagy in yeast is independent of mitochondrial fission and requires the stress response gene WHI2. J Cell Sci. 2011 Apr 15;124(Pt 8):1339-50.

123. Kakuta S, Yamamoto H, Negishi L, et al. Atg9 vesicles recruit vesicle-tethering proteins Trs85 and Ypt1 to the autophagosome formation site. J Biol Chem. 2012 Dec 28;287(53):44261-9.

124. Zou S, Chen Y, Liu Y, et al. Trs130 participates in autophagy through GTPases Ypt31/32 in Saccharomyces cerevisiae. Traffic. 2013 Feb;14(2):233-46.
