# Supplementary material for: An APEX2-based proximity-dependent biotinylation assay with temporal specificity to study protein interactions during autophagy in the yeast Saccharomyces cerevisiae
Source: Autophagy. 2024 Jul 3;20(10):2323–37. doi: 10.1080/15548627.2024.2366749 (PMC11423678; doi:10.1080/15548627.2024.2366749)
Supplement: Supplemental Material [file KAUP_A_2366749_SM8137.zip › Table_S4.pdf]

Table S4. Atg9 interactors in rich medium. Known roles of the detected proteins in yeast autophagy are indicated, as well if they were identified in other autophagy-related proteomics analyses.

Enriched interactors (BH corrected p-value < 0.05)		
**Protein**	**Autophagy-related function(s) in yeast**	**Other MS analyses**
Acb1	Secretory autophagosome cargo [1]; negative regulator of autophagy [2]	
Ade12	-	
Aim36	-	
Aim39	-	
Ald5	-	
Aro2	-	
Aro8	-	
Arp3	Part of the Arp2/3 complex, which regulates Atg9 trafficking during selective types of autophagy [3]; involved in ER-phagy [4]	
Atg11	Atg machinery core component, adaptor protein for all types of selective autophagy [5,6]; directly interacts with Atg9 [7,8]	
Bat1	-	
Bna2	-	
Cab1	-	
Car2	-	
Ccc1	-	
Ccp1	-	
Ccs1	-	
Cdc10	Septin found in close proximity of Atg9, possibly involved in Atg9 trafficking [9]	
Cdc12	-	
Cmc2	-	
Coa4	-	
Coi1	-	
Cox13	-	
Ctt1	-	
Cwh41	-	
Cyc8	-	
Dpc7	-	
Ddr48	-	
Dhh1	Promotes Atg1 and Atg13 translation during nitrogen starvation while promoting the degradation of *ATG* transcripts during nutrient-rich condition, regulating autophagy [10,11]	
Dld1	-	
Dug1	-	
Dys1	-	
Emi2	-	
Erv1	-	

Fra1	-	
Frs2	Candidate autophagosomal cargo [12]	
Fur1	-	
Gcs1	-	
Gcv2	-	
Gdh1	Candidate autophagosomal cargo [12]	
Glk1	Candidate autophagosomal cargo [12]	
Gua1	-	
Guk1	-	
Gut2	-	
Hri1	-	
Hsp12	-	
Hts1	-	
Igo1	Phosphorylated Igo1 directly inhibits the Cdc55 phosphatase [13], which is required for sufficient Atg13 dephosphorylation and autophagy induction after TORC1 inactivation [14]; required for pre-meiotic autophagy [15]	
Ino1	-	
Ipp1	Candidate autophagosomal cargo [12]	
Krs1	Candidate autophagosomal cargo [12]	
Leu2	-	
Lys12	-	
Mam3	-	
Mcr1	-	
Met13	-	
Met17	Candidate autophagosomal cargo [12]	
Mgr1	Subunit of the i-AAA protease complex, which is involved in the proteolytical processing of Atg32 necessary to regulate mitophagy [16]	
Mic60	-	
Mns1	-	
Mpm1	-	
Mrp35	-	
Ncp1	-	
Npt1	-	
Nup159	Autophagy receptor for the selective autophagy of nuclear pore complexes [17,18]; required for Snx4-assisted vacuolar targeting of transcription factors controlling *ATG* gene expression [19]	
Om45	-	
Pai3	Inhibitor of Pep4 [20], the major vacuolar protease essential for the degradation of autophagosomal cargoes [21]	
Pep4	Major vacuolar protease, which is essential for the degradation of autophagosomal cargoes [21]	
Pet117	-	
Pup2	Subunit of the 26S proteasome, which is targeted by selective autophagy [22-24]	
Pwp1	-	
Rdi1	-	
Ret2	-	

Rfs1	-	[25]
Rie1	-	
Rpb3	-	
Rpn12	Subunit of the 26S proteasome, which is targeted for selective autophagy [22-24]	
Rpn6	Subunit of the 26S proteasome, which is targeted by selective autophagy [22-24]	
Sco1	-	
Sco2	-	
Spe3	-	
Sup45	-	
Ths1	-	
Tim11	-	
Tim50	-	
Tpi1	-	
Tpk1	Catalytic subunit of PKA, which regulates autophagy [26-28]	
Tps1	-	
Tub2	-	
Tum1	-	
Tyw1	-	
Uba1	Candidate autophagosomal cargo [12]	
Ura3	-	
Ura4	-	
Uso1	-	
Vma6	Subunit of the V-ATPase involved in acidification of the vacuolar lumen, which is essential for the degradation of autophagosomal cargoes [29]	
Vph1	Subunit of the V-ATPase involved in acidification of the vacuolar lumen, which is essential for the degradation of autophagosomal cargoes [29]; selectively degraded by ESCRT-dependent microautophagy of the vacuole [30]	
Vps35	Involved in Atg9 trafficking [31]	
Vtc4	Subunit of the Vacuolar transporter chaperone complex, which is required for microautophagy [32]	
Wwm1	-	
Ydl012c	-	
Yfr006w	-	
Yjr154w	-	
Ykl091c		
Ynl134c	Candidate autophagosomal cargo [12]	
Ynl208w	-	
Enriched interactors (p-value < 0.05)		
**Protein**	**Autophagy-related function(s) in yeast**	**Other MS analyses**
Ahp1	Candidate autophagosomal cargo [12]	
Aim18	-	
Aim6	-	
Alg9	-	
Apl5	Involved in the Atg27 trafficking [33]	[34]

Apm3	Involved in the Atg27 trafficking [33]	
Aro1	-	
Arp2	Interacts with Atg9 and required for its trafficking during selective types of autophagy [4]; involved in ER-phagy [4]	
Atg2	Atg machinery core component [35,36], which also interacts with Atg9 [37]	
Atg9	Atg9 self-interacts by forming a trimer [38-40]	[25,41,42]
Atg17	Atg machinery core component involved in autophagy initiation and Atg9 trafficking [43,44], autophagosome closure [45] and autophagosome fusion with vacuoles [46]; interacts with Atg9 [43]	
Atg27	Binds to Atg9 and it is involved in its trafficking [47]	[25,41,42]
Bgl2	-	
Blm10	Proteasome activator which is degraded by autophagy both bound to the core particle of the proteasome and in its unbound form [48]	
Caj1	-	
Cap2	-	
Cct3	-	
Cdc11	Septin involved in autophagy [9]	
Cdc24	-	
Cdc28	-	
Cdc3	-	
Cdc37	-	
Cdc48	Binding partner of Atg8 involved in autophagosome formation [53], ribophagy [49], micronucleophagy [50] and granulophagy [51]	[41]
Cir2	-	
Cox17	-	
Cyc1	-	
Cyc7	-	
Dcp1	Subunit of the Dcp1-Dcp2 decapping complex, which is involved in the regulation of *ATG* mRNA stability [11]	
Dld3	Candidate autophagosomal cargo [12]	
Doa1	-	
Dus4	-	
Ecm14	-	
End3	Involved in selective autophagy of aberrant CME protein assemblies via the SAR Ede1 [52]; required for ER-phagy [4]	
Erg20	-	
Erg6	-	
Erv25	-	[41]
Fcy1	-	
Fmp46	-	
Frs1	-	
Fsh1	-	
Gdh2	-	
Gdi1	-	
Glc8	-	
Glo2	-	
Gnd1	Candidate autophagosomal cargo [12]	

Gnd2	-	
Gpn2	-	
Gsh1	-	
Gvp36	Cargo of Cue5-mediated aggrephagy [53]. As Atg9, involved in sphingolipid homeostasis [54]	[25,41]
Hat2	-	
His7	-	
Hog1	Mitophagy regulator [55,56]	
Hsp26	-	
Ira1	-	
Irr1	-	
Kre2	-	
Lsm2	Subunit of the Pat1-Lsm complex, which stabilizes *ATG* mRNAs during autophagy [57]	
Mae1	-	
Mdh1	-	
Mdm35	-	
Met22	-	
Met3	-	
Met6	-	[25]
Mia40	-	
Mix23	-	
Mrx18	-	
Mtc1	-	
Mtr4	-	
New1	-	
Npc2	Essential for the formation of raft-like vacuolar microdomains and lipid droplets engulfment by vacuoles via microlipophagy [58]	
Npl4	-	
Nup57	Subunit of the nuclear pore complex degraded by selective autophagy [17]	
Nup85	Subunit of the nuclear pore complex degraded by selective autophagy [17]	
Osh7	Involved in piecemeal microautophagy of the nucleus [59]	
Oye2	-	
Pfd1	-	
Pga3	-	
Pgi1	Candidate autophagosomal cargo [12]	
Pho81	-	
Pir1	-	
Pnc1	-	
Pop2	Subunit of the Ccr4-Not1 core complex, which regulates mRNA levels of several *ATG* genes [60]	
Pre7	Subunit of the 26S proteasome, which is targeted by selective autophagy [22-24]	
Prm8	-	
Ptc7	-	
Pub1	Component of stress granules, which are degraded by autophagy [51]	

Puf4	-	
Pup1	Subunit of the 26S proteasome, which is targeted by selective autophagy [22-24]	
Ras2	Autophagy regulator [28]	
Rcf2	-	
Rci37	-	
Rna15	-	
Rpb2	-	
Rpn10	Subunit of the 26S proteasome, which is targeted by selective autophagy [22-24]	
Rpo21	-	
Rps10A; Rps10B	-	
Rpt4	Subunit of the 26S proteasome, which is targeted by selective autophagy [22-24]	
Rpt6	Subunit of the 26S proteasome, which is targeted by selective autophagy [22-24]	
Rsp5	Ubiquitin ligase involved in the selective autophagy of aggregates, the proteasomes, mitochondria and possibly ribosomes [24,53,61,62]; involved in microautophagy of vacuolar membrane proteins and proteasomes [30,63]	
Rtc3	-	
Sam1	-	
Scd6	Component of stress granules, which are degraded by autophagy [51]	
Sds22	-	
Sec14	Important to regulate the levels of PtdIns4P, which are important for Atg9 trafficking and thus autophagy [64]	
Sec16	Subunit of COPII vesicles, which are a membrane source for autophagosome biogenesis [41,65,66] and are involved in Atg9 sorting out of the ER [67]	
Sec31	Subunit of COPII vesicles, which are a membrane source for autophagosome biogenesis [41,65,66] and are involved in Atg9 sorting out of the ER [67]	
Ser1	-	
Sfm1	-	
Ski2	-	
Skp1	-	
Slm1	-	
Smi1	-	
Sna4	Vacuolar protein degraded by microautophagy [68]	
Sol3	-	
Sqt1	-	
Srp1	-	
Ssa1	-	
Sso2	Required for Atg9 trafficking and autophagy [69]	
Tbf1	-	
Thi20	-	
Tif6	-	

Tkl1	-	
Tpm2	-	
Trp3	-	
Trx1	Autophagy regulator [70]; candidate autophagosomal cargo [12]	
Tub3	-	
Ubc7	-	
Ura6	-	
Utr2	-	
Vac14	-	
Vma1	Subunit of the V-ATPase involved in acidification of the vacuolar lumen, which is essential for the degradation of autophagosomal cargoes [29]	
Vma13	Subunit of the V-ATPase involved in acidification of the vacuolar lumen, which is essential for the degradation of autophagosomal cargoes [29]	
Vma2	Subunit of the V-ATPase involved in acidification of the vacuolar lumen, which is essential for the degradation of autophagosomal cargoes [29]	
Vma5	Subunit of the V-ATPase involved in acidification of the vacuolar lumen, which is essential for the degradation of autophagosomal cargoes [29]; candidate autophagosomal cargo [12]	
Vps1	Involved in Atg9 trafficking [71]; involved in pexophagy [72]	[41]
Vps29	Involved in Atg9 trafficking [31]	
Vps34	Atg machinery core component generating phosphatidylinositol 3-phosphate, which is essential for autophagosome formation [73]	
Ybl055c	-	
Yck1	-	[41]
Ycp4	-	
Ydj1	Candidate autophagosomal cargo [12]	
Ydl086w	-	
Ygr017w	-	
Yhc1	-	
Yij108w	-	
Yjr096w	-	
Ymr196w	-	
Ypr127w	-	
Yrr1	-	
Zwf1	Glucose-6-phosphate dehydrogenase that negatively modulates autophagy [74]	

References

1. Duran JM, Anjard C, Stefan C, et al. Unconventional secretion of Acb1 is mediated by autophagosomes. J Cell Biol. 2010 Feb 22;188(4):527-36.

2. Montegut L, Joseph A, Chen H, et al. DBI/ACBP is a targetable autophagy checkpoint involved in aging and cardiovascular disease. Autophagy. 2023 Jul;19(7):2166-2169.

3. Monastyrska I, He C, Geng J, et al. Arp2 Links Autophagic Machinery with the Actin Cytoskeleton. Molecular Biology of the Cell. 2008;19(5):1962-1975.

4. Liu D, Mari M, Li X, et al. ER-phagy requires the assembly of actin at sites of contact between the cortical ER and endocytic pits. Proc Natl Acad Sci U S A. 2022 Feb 8;119(6).

5. Shintani T, Huang W-P, Stromhaug PE, et al. Mechanism of cargo selection in the cytoplasm to vacuole targeting pathway. Dev Cell. 2002 Dec;3(6):825-37.

6. Suzuki K, Kamada Y, Ohsumi Y. Studies of cargo delivery to the vacuole mediated by autophagosomes in *Saccharomyces cerevisiae*. Dev Cell. 2002 Dec;3(6):815-24.

7. He C, Song H, Yorimitsu T, et al. Recruitment of Atg9 to the preautophagosomal structure by Atg11 is essential for selective autophagy in budding yeast. J Cell Biol. 2006 Dec 18;175(6):925-35.

8. Matscheko N, Mayrhofer P, Rao Y, et al. Atg11 tethers Atg9 vesicles to initiate selective autophagy. PLoS Biol. 2019 Jul;17(7):e3000377.

9. Barve G, Sridhar S, Aher A, et al. Septins are involved at the early stages of macroautophagy in S. cerevisiae. J Cell Sci. 2018 Feb 22;131(4).

10. Liu X, Yao Z, Jin M, et al. Dhh1 promotes autophagy-related protein translation during nitrogen starvation. PLoS Biol. 2019 Apr;17(4):e3000219.

11. Hu G, McQuiston T, Bernard A, et al. A conserved mechanism of TOR-dependent RCK-mediated mRNA degradation regulates autophagy. Nat Cell Biol. 2015 Jul;17(7):930-942.

12. Suzuki K, Nakamura S, Morimoto M, et al. Proteomic profiling of autophagosome cargo in Saccharomyces cerevisiae. PLoS One. 2014;9(3):e91651.

13. Bontron S, Jaquenoud M, Vaga S, et al. Yeast endosulfines control entry into quiescence and chronological life span by inhibiting protein phosphatase 2A. Cell Rep. 2013 Jan 31;3(1):16-22.

14. Yeasmin AM, Waliullah TM, Kondo A, et al. Orchestrated Action of PP2A Antagonizes Atg13 Phosphorylation and Promotes Autophagy after the Inactivation of TORC1. PLoS One. 2016;11(12):e0166636.

15. Sarkar S, Dalgaard JZ, Millar JB, et al. The Rim15-endosulfine-PP2ACdc55 signalling module regulates entry into gametogenesis and quiescence via distinct mechanisms in budding yeast. PLoS Genet. 2014 Jun;10(6):e1004456.

16. Wang K, Jin M, Liu X, et al. Proteolytic processing of Atg32 by the mitochondrial i-AAA protease Yme1 regulates mitophagy. Autophagy. 2013 Nov 1;9(11):1828-36.

17. Tomioka Y, Kotani T, Kirisako H, et al. TORC1 inactivation stimulates autophagy of nucleoporin and nuclear pore complexes. J Cell Biol. 2020 Jul 6;219(7).

18. Lee CW, Wilfling F, Ronchi P, et al. Selective autophagy degrades nuclear pore complexes. Nat Cell Biol. 2020 Feb;22(2):159-166.

19. Hanley SE, Willis SD, Cooper KF. Snx4-assisted vacuolar targeting of transcription factors defines a new autophagy pathway for controlling ATG expression. Autophagy. 2021 Nov;17(11):3547-3565.

20. Schu P, Wolf DH. The proteinase yscA-inhibitor, IA3, gene. Studies of cytoplasmic proteinase inhibitor deficiency on yeast physiology. FEBS Lett. 1991 May 20;283(1):78-84.

21. Takeshige K, Baba M, Tsuboi S, et al. Autophagy in yeast demonstrated with proteinase-deficient mutants and conditions for its induction. Journal of Cell Biology. 1992;119(2):301-311.

22. Waite KA, De-La Mota-Peynado A, Vontz G, et al. Starvation Induces Proteasome Autophagy with Different Pathways for Core and Regulatory Particles. J Biol Chem. 2016 Feb 12;291(7):3239-53.

23. Marshall RS, McLoughlin F, Vierstra RD. Autophagic Turnover of Inactive 26S Proteasomes in Yeast Is Directed by the Ubiquitin Receptor Cue5 and the Hsp42 Chaperone. Cell Rep. 2016 Aug 9;16(6):1717-1732.

24. Marshall RS, Vierstra RD. A trio of ubiquitin ligases sequentially drives ubiquitylation and autophagic degradation of dysfunctional yeast proteasomes. Cell Rep. 2022 Mar 15;38(11):110535.

25. Kakuta S, Yamamoto H, Negishi L, et al. Atg9 vesicles recruit vesicle-tethering proteins Trs85 and Ypt1 to the autophagosome formation site. J Biol Chem. 2012 Dec 28;287(53):44261-9.

26. Stephan JS, Yeh Y-Y, Ramachandran V, et al. The Tor and PKA signaling pathways independently target the Atg1/Atg13 protein kinase complex to control autophagy. Proceedings of the National Academy of Sciences. 2009;106(40):17049-17054.

27. Yorimitsu T, Zaman S, Broach JR, et al. Protein kinase A and Sch9 cooperatively regulate induction of autophagy in Saccharomyces cerevisiae. Mol Biol Cell. 2007 Oct;18(10):4180-9.

28. Budovskaya YV, Stephan JS, Reggiori F, et al. The Ras/cAMP-dependent protein kinase signaling pathway regulates an early step of the autophagy process in Saccharomyces cerevisiae. J Biol Chem. 2004 May 14;279(20):20663-71.

29. Nakamura N, Matsuura A, Wada Y, et al. Acidification of Vacuoles Is Required for Autophagic Degradation in the Yeast, *Saccharomyces cerevisiae*. The Journal of Biochemistry. 1997;121(2):338-344.

30. Yang X, Zhang W, Wen X, et al. TORC1 regulates vacuole membrane composition through ubiquitin- and ESCRT-dependent microautophagy. J Cell Biol. 2020 Mar 2;219(3).

31. Marquardt L, Taylor M, Kramer F, et al. Vacuole fragmentation depends on a novel Atg18-containing retromer-complex. Autophagy. 2023 Jan;19(1):278-295.

32. Uttenweiler A, Schwarz H, Neumann H, et al. The vacuolar transporter chaperone (VTC) complex is required for microautophagy. Mol Biol Cell. 2007 Jan;18(1):166-75.

33. Segarra VA, Boettner DR, Lemmon SK. Atg27 tyrosine sorting motif is important for its trafficking and Atg9 localization. Traffic. 2015 Apr;16(4):365-78.

34. Schoppe J, Mari M, Yavavli E, et al. AP-3 vesicle uncoating occurs after HOPS-dependent vacuole tethering. EMBO J. 2020 Oct 15;39(20):e105117.

35. Shintani T, Suzuki K, Kamada Y, et al. Apg2p functions in autophagosome formation on the perivacuolar structure. J Biol Chem. 2001 Aug 10;276(32):30452-60.

36. Wang CW, Kim J, Huang WP, et al. Apg2 is a novel protein required for the cytoplasm to vacuole targeting, autophagy, and pexophagy pathways. J Biol Chem. 2001 Aug 10;276(32):30442-51.

37. Gomez-Sanchez R, Rose J, Guimaraes R, et al. Atg9 establishes Atg2-dependent contact sites between the endoplasmic reticulum and phagophores. J Cell Biol. 2018 Aug 6;217(8):2743-2763.

38. Reggiori F, Shintani T, Nair U, et al. Atg9 cycles between mitochondria and the pre-autophagosomal structure in yeasts. Autophagy. 2005 Jul;1(2):101-9.

39. He C, Baba M, Cao Y, et al. Self-interaction is critical for Atg9 transport and function at the phagophore assembly site during autophagy. Mol Biol Cell. 2008 Dec;19(12):5506-16.

40. Matoba K, Kotani T, Tsutsumi A, et al. Atg9 is a lipid scramblase that mediates autophagosomal membrane expansion. Nat Struct Mol Biol. 2020 Dec;27(12):1185-1193.

41. Graef M, Friedman JR, Graham C, et al. ER exit sites are physical and functional core autophagosome biogenesis components. Mol Biol Cell. 2013 Sep;24(18):2918-31.

42. Sawa-Makarska J, Baumann V, Coudevylle N, et al. Reconstitution of autophagosome nucleation defines Atg9 vesicles as seeds for membrane formation. Science. 2020 Sep 4;369(6508).

43. Sekito T, Kawamata T, Ichikawa R, et al. Atg17 recruits Atg9 to organize the pre-autophagosomal structure. Genes Cells. 2009 May;14(5):525-38.

44. Rao Y, Perna MG, Hofmann B, et al. The Atg1-kinase complex tethers Atg9-vesicles to initiate autophagy. Nat Commun. 2016 Jan 12;7:10338.

45. Zhou F, Wu Z, Zhao M, et al. Rab5-dependent autophagosome closure by ESCRT. J Cell Biol. 2019 Jun 3;218(6):1908-1927.

46. Liu X, Mao K, Yu AYH, et al. The Atg17-Atg31-Atg29 Complex Coordinates with Atg11 to Recruit the Vam7 SNARE and Mediate Autophagosome-Vacuole Fusion. Curr Biol. 2016 Jan 25;26(2):150-160.

47. Legakis JE, Yen W-L, Klionsky DJ. A Cycling Protein Complex Required for Selective Autophagy. Autophagy. 2007;3(5):422-432.

48. Burris A, Waite KA, Reuter Z, et al. Proteasome activator Blm10 levels and autophagic degradation directly impact the proteasome landscape. J Biol Chem. 2021 Jan-Jun;296:100468.

49. Ossareh-Nazari B, Bonizec M, Cohen M, et al. Cdc48 and Ufd3, new partners of the ubiquitin protease Ubp3, are required for ribophagy. EMBO Rep. 2010 Jul;11(7):548-54.

50. Krick R, Bremer S, Welter E, et al. Cdc48/p97 and Shp1/p47 regulate autophagosome biogenesis in concert with ubiquitin-like Atg8. J Cell Biol. 2010 Sep 20;190(6):965-73.

51. Buchan JR, Kolaitis RM, Taylor JP, et al. Eukaryotic stress granules are cleared by autophagy and Cdc48/VCP function. Cell. 2013 Jun 20;153(7):1461-74.

52. Wilfling F, Lee CW, Erdmann PS, et al. A Selective Autophagy Pathway for Phase-Separated Endocytic Protein Deposits. Mol Cell. 2020 Dec 3;80(5):764-778 e7.

53. Lu K, Psakhye I, Jentsch S. Autophagic clearance of polyQ proteins mediated by ubiquitin-Atg8 adaptors of the conserved CUET protein family. Cell. 2014 Jul 31;158(3):549-63.

54. Lebesgue N, Megyeri M, Cristobal A, et al. Combining Deep Sequencing, Proteomics, Phosphoproteomics, and Functional Screens To Discover Novel Regulators of Sphingolipid Homeostasis. J Proteome Res. 2017 Feb 3;16(2):571-582.

55. Mao K, Wang K, Zhao M, et al. Two MAPK-signaling pathways are required for mitophagy in Saccharomyces cerevisiae. J Cell Biol. 2011 May 16;193(4):755-67.

56. Aoki Y, Kanki T, Hirota Y, et al. Phosphorylation of Serine 114 on Atg32 mediates mitophagy. Mol Biol Cell. 2011 Sep;22(17):3206-17.

57. Gatica D, Hu G, Liu X, et al. The Pat1-Lsm Complex Stabilizes ATG mRNA during Nitrogen Starvation-Induced Autophagy. Mol Cell. 2019 Jan 17;73(2):314-324 e4.

58. Tsuji T, Fujimoto M, Tatematsu T, et al. Niemann-Pick type C proteins promote microautophagy by expanding raft-like membrane domains in the yeast vacuole. Elife. 2017 Jun 7;6.

59. Kvam E, Goldfarb DS. Nvj1p is the outer-nuclear-membrane receptor for oxysterol-binding protein homolog Osh1p in Saccharomyces cerevisiae. J Cell Sci. 2004 Oct 1;117(Pt 21):4959-68.

60. Yin Z, Zhang Z, Lei Y, et al. Bidirectional roles of the Ccr4-Not complex in regulating autophagy before and after nitrogen starvation. Autophagy. 2023 Feb;19(2):415-425.

61. Belgareh-Touze N, Cavellini L, Cohen MM. Ubiquitination of ERMES components by the E3 ligase Rsp5 is involved in mitophagy. Autophagy. 2017 Jan 2;13(1):114-132.

62. Kraft C, Peter M. Is the Rsp5 ubiquitin ligase involved in the regulation of ribophagy? Autophagy. 2008 Aug;4(6):838-40.

63. Li J, Hochstrasser M. Selective microautophagy of proteasomes is initiated by ESCRT-0 and is promoted by proteasome ubiquitylation. J Cell Sci. 2022 Feb 15;135(4).

64. Wang K, Yang Z, Liu X, et al. Phosphatidylinositol 4-kinases are required for autophagic membrane trafficking. J Biol Chem. 2012 Nov 2;287(45):37964-72.

65. Suzuki K, Kubota Y, Sekito T, et al. Hierarchy of Atg proteins in pre-autophagosomal structure organization. Genes Cells. 2007 Feb;12(2):209-18.

66. Tan D, Cai Y, Wang J, et al. The EM structure of the TRAPPIII complex leads to the identification of a requirement for COPII vesicles on the macroautophagy pathway. Proc Natl Acad Sci U S A. 2013 Nov 26;110(48):19432-7.

67. Mari M, Griffith J, Rieter E, et al. An Atg9-containing compartment that functions in the early steps of autophagosome biogenesis. J Cell Biol. 2010 Sep 20;190(6):1005-22.

68. Morshed S, Tasnin MN, Ushimaru T. ESCRT machinery plays a role in microautophagy in yeast. BMC Mol Cell Biol. 2020 Oct 7;21(1):70.

69. Nair U, Jotwani A, Geng J, et al. SNARE proteins are required for macroautophagy. Cell. 2011 Jul 22;146(2):290-302.

70. Perez-Perez ME, Zaffagnini M, Marchand CH, et al. The yeast autophagy protease Atg4 is regulated by thioredoxin. Autophagy. 2014;10(11):1953-64.

71. Arlt H, Raman B, Filali-Mouncef Y, et al. The dynamin Vps1 mediates Atg9 transport to the sites of autophagosome formation. J Biol Chem. 2023 May;299(5):104712.

72. Mao K, Liu X, Feng Y, et al. The progression of peroxisomal degradation through autophagy requires peroxisomal division. Autophagy. 2014 Apr;10(4):652-61.

73. Kihara A, Noda T, Ishihara N, et al. Two distinct Vps34 phosphatidylinositol 3-kinase complexes function in autophagy and carboxypeptidase Y sorting in *Saccharomyces cerevisiae*. J Cell Biol. 2001 Feb 5;152(3):519-30.

74. Delorme-Axford E, Wen X, Klionsky DJ. The yeast transcription factor Stb5 acts as a negative regulator of autophagy by modulating cellular metabolism. Autophagy. 2023 Jul 2:1-14.
