# Supplementary material for: An APEX2-based proximity-dependent biotinylation assay with temporal specificity to study protein interactions during autophagy in the yeast Saccharomyces cerevisiae
Source: Autophagy. 2024 Jul 3;20(10):2323–37. doi: 10.1080/15548627.2024.2366749 (PMC11423678; doi:10.1080/15548627.2024.2366749)
Supplement: Supplemental Material [file KAUP_A_2366749_SM8137.zip › Table_S5.docx]

# **Table S5. Atg9 interactors upon 1 h of nitrogen starvation.** Known roles of the detected proteins in yeast autophagy are indicated, as well if they were identified in other autophagy-related proteomics analyses.

| Enriched interactors (BH corrected p-value < 0.05) | | |
| --- | --- | --- |
| **Protein** | **Autophagy-related function(s) in yeast** | **Other MS analyses** |
| Aim36 | - |  |
| Atg9 | Atg9 self-interacts by forming a trimer [1-3] | [4-6] |
| Atg21 | Atg machinery core component involved in the recruitment of the Atg12–Atg5-Atg16 complex to the PAS [7] |  |
| Atg23 | Binds to Atg9 and it is involved in its trafficking [8]; membrane tethering by Atg23 is essential for autophagy [9] | [4-6] |
| Atg27 | Binds to Atg9 and it is involved in its trafficking [8] | [4-6] |
| Bmh1 | Participates in the regulation of *ATG8* transcription [10]; candidate autophagosomal cargo [11] |  |
| Bna2 | - |  |
| Ccp1 | - |  |
| Cmc2 | - |  |
| Cox17 | - |  |
| Cox5a | - |  |
| Csc1 | - |  |
| Ddr48 | - |  |
| Egd2 | Candidate autophagosomal cargo [11] |  |
| Ent3 | Involved in Atg27 trafficking [12] |  |
| Erv1 | - |  |
| Fra1 | - |  |
| Gfd2 | - |  |
| Glc7 | - |  |
| Gvp36 | Cargo of Cue5-mediated aggrephagy [13]. As Atg9, involved in sphingolipid homeostasis [14] | [4,6] |
| Hbt1 | - |  |
| Hri1 | - |  |
| Hsp12 | - |  |
| Igd1 | - |  |
| Igo1 | Phosphorylated Igo1 directly inhibits the Cdc55 phosphatase [15], which is required for sufficient Atg13 dephosphorylation and autophagy induction after TORC1 inactivation [16]; required for pre-meiotic autophagy [17] |  |
| Ino1 | - |  |
| Lia1 | Candidate autophagosomal cargo [11] |  |
| Mam3 | - |  |
| Mca1 | - |  |
| Met17 | - |  |
| Met3 | - |  |
| Mia40 | - |  |
| Mic60 | - |  |
| Mnr2 | Interacts with Atg9 in a large-scale split ubiquitin screen [18] |  |
| Om45 | - |  |
| Pai3 | Inhibitor of Pep4 [19], the major vacuolar protease essential for the degradation of autophagosomal cargoes [20] |  |
| Pep4 | Major vacuolar protease essential for the degradation of autophagosomal cargoes [20] |  |
| Ras1 | Autophagy regulator [21] |  |
| Rpn12 | Subunit of the 26S proteasome, which is targeted by selective autophagy [22-24] |  |
| Rtc3 | - |  |
| Rvs167 | - |  |
| Scd6 | Component of stress granules, which are degraded by autophagy [25] |  |
| Sco1 | - |  |
| Sco2 | - |  |
| Shp1 | Binding partner of Atg8 involved in autophagosome formation [26] |  |
| Smi1 | - |  |
| Spe3 | - |  |
| Tim11 | - |  |
| Tim50 | - |  |
| Uip4 | - |  |
| Vma5 | Subunit of the V-ATPase involved in acidification of the vacuolar lumen, which is essential for the degradation of the autophagosomal cargoes [27] |  |
| Vps35 | Involved in Atg9 trafficking [28] |  |
| Wwm1 | - |  |
| Yfr006w | - |  |
| Ynl208w | - |  |
| Enriched interactors (p-value < 0.05) | | |
| **Protein** | **Autophagy-related function(s) in yeast** | **Other MS analyses** |
| Acb1 | Secretory autophagosome cargo [29]; negative regulator of autophagy [30] | - |
| Acf2 | Possible negative regulator of mitophagy [31] |  |
| Adh5 | - |  |
| Aim18 | - |  |
| Aim2 | - |  |
| Aip1 | - |  |
| Ape3 | - |  |
| Arc1 | - |  |
| Ard1 | Involved in mitophagy induction in yeast [32] |  |
| Arp2 | Interacts with Atg9 and required for its trafficking during selective types of autophagy [33]; involved in ER-phagy [34] |  |
| Asp1 | - |  |
| Atg1 | Atg machinery core component, interacts with Atg9 via Atg13 [35] or Atg17 [36]; directly phosphorylates Atg9 [37] |  |
| Atg14 | Atg machinery core component generating phosphatidylinositol 3-phosphate, which is essential for autophagosome formation [38]; possibly recruited by Atg9 to the PAS [35] |  |
| Atg17 | Atg machinery core component involved in autophagy initiation and Atg9 trafficking [36,39], autophagosome closure [40] and autophagosome fusion with vacuoles [41]; interacts with Atg9 [39] |  |
| Bgl2 | - |  |
| Bmh2 | Participates in the regulation of *ATG8* transcription [10]; candidate autophagosomal cargo [11] |  |
| Bna1 | - |  |
| Cap2 | - |  |
| Ccs1 | - |  |
| Cdc10 | Septin found in close proximity of Atg9, possibly involved in Atg9 trafficking [42] |  |
| Cdc11 | Septin involved in autophagy [42] |  |
| Cdc55 | Required together with Rts1 for sufficient Atg13 dephosphorylation and autophagy induction after TORC1 inactivation [16]; promotes microautophagy [43] |  |
| Coa4 | - |  |
| Ctt1 | - |  |
| Cyb2 | - |  |
| Cub1 | - |  |
| Cvm1 | - |  |
| Dcp1 | Subunit of the Dcp1-Dcp2 decapping complex, which is involved in the regulation of *ATG* mRNA stability [44] |  |
| Ddi1 | - |  |
| Dld1 | - |  |
| Doa1 | - |  |
| Dop1 | - |  |
| Dys1 | - |  |
| Ecm19 | - |  |
| Fmp10 | - |  |
| Gdh1 | - |  |
| Glc8 | - |  |
| Grx1 | - |  |
| Guk1 | - |  |
| Hpa3 | - |  |
| Hsp104 | Candidate autophagosomal cargo [11] |  |
| Hsp26 | - |  |
| Hsp42 | Involved in proteasome turnover by selective autophagy [23,24] |  |
| Iki1 | - |  |
| Ira1 | - |  |
| Kap123 | Candidate autophagosomal cargo [11] |  |
| Leu2 | - |  |
| Lsc2 | - |  |
| Lsg1 | - |  |
| Lsm2 | Subunit of the Pat1-Lsm complex, which stabilizes *ATG* mRNA during autophagy [45] |  |
| Mbf1 | - |  |
| Mcr1 | - |  |
| Mpm1 | - |  |
| Npc2 | Essential for the formation of raft-like vacuolar microdomains and lipid droplets engulfment by vacuoles via microlipophagy [46] |  |
| Npl6 | Subunit of the Rsc1-RSC chromatin remodeling complex, which is required for autophagy induction [47] |  |
| Pep12 | Involved in autophagosome closure [48] | [4] |
| Vps11 | Subunit of the HOPS tethering complex, which is required for autophagosome-vacuole fusion [49] |  |
| Pnc1 | - |  |
| Pst2 | - |  |
| Ptc5 | - |  |
| Ptc7 | - |  |
| Ptp1 | - |  |
| Pup2 | Subunit of the 26S proteasome, which is targeted by selective autophagy [22-24] |  |
| Pwp1 | - |  |
| Rcf2 | - |  |
| Rdi1 | - |  |
| Ret2 | - |  |
| Rfs1 | - | [6] |
| Rie1 | - |  |
| Rim20 | - |  |
| Rpb3 | - |  |
| Rpn6 | Subunit of the 26S proteasome, which is targeted by selective autophagy [22-24] |  |
| Rpn7 | Subunit of the 26S proteasome, which is targeted by selective autophagy [22-24] |  |
| Rpp2a | Subunit of the 60S ribosome, which is selectively degraded by autophagy [50] |  |
| Rrp9 | - |  |
| Rtn2 | - |  |
| Scw4 | - |  |
| Sec13 | Subunit of COPII vesicles, which are a membrane source for autophagosome biogenesis [4,51,52] and are involved in Atg9 sorting out of the ER [53] |  |
| Sec28 | - |  |
| Sec31 | Subunit of COPII vesicles, which are a membrane source for autophagosome biogenesis [4,51,52] and are involved in Atg9 sorting out of the ER [53] |  |
| Sec9 | Involved in Atg9 trafficking [54] |  |
| Sfb2 | Subunit of COPII vesicles, which are a membrane source for autophagosome biogenesis [4,51,52] and are involved in Atg9 sorting out of the ER [53] |  |
| Sft1 | Possibly involved in Atg9 trafficking [55] | [4,5] |
| Sgt2 | Candidate autophagosomal cargo [11] |  |
| Skp1 | - |  |
| Sna4 | Vacuolar protein degraded by microautophagy [56] |  |
| Ssa1 | Candidate autophagosomal cargo [11] |  |
| Sse1 | Candidate autophagosomal cargo [11] |  |
| Sti1 | Candidate autophagosomal cargo [11] |  |
| Stv1 | Subunit of the V-ATPase involved in acidification of the vacuolar lumen, which is essential for the degradation of autophagosomal cargoes [27] | [4] |
| Sui1 | - |  |
| Syn8 | - | [6] |
| Tif1 | Candidate autophagosomal cargo [11] |  |
| Tif6 | - |  |
| Tlg2 | SNARE involved in Atg9 trafficking and autophagy [54] | [4] |
| Tpa1 | - |  |
| Tpi1 | Candidate autophagosomal cargo [11] |  |
| Tpk1 | Catalytic subunit of PKA, which regulates autophagy [21,57,58] |  |
| Tpm2 | - |  |
| Trx1 | Autophagy regulator [59] |  |
| Trx2 | Autophagy regulator [59] |  |
| Tsa1 | Cargo of Cue5-mediated aggrephagy [13]; candidate autophagosomal cargo [11] |  |
| Tsa2 | - |  |
| Tub3 | - |  |
| Ubp15 | - |  |
| Ura3 | - |  |
| Vac14 | - |  |
| Vma2 | Subunit of the V-ATPase involved in acidification of the vacuolar lumen, which is essential for the degradation of autophagosomal cargoes **[27]** |  |
| Vma4 | Subunit of the V-ATPase involved in acidification of the vacuolar lumen, which is essential for the degradation of autophagosomal cargoes **[27]** |  |
| Vma6 | Subunit of the V-ATPase involved in acidification of the vacuolar lumen, which is essential for the degradation of autophagosomal cargoes **[27]** |  |
| Vps29 | Involved in Atg9 trafficking [28] |  |
| Vps34 | Atg machinery core component generating phosphatidylinositol 3-phosphate, which is essential for autophagosome formation [60] |  |
| Ybr085c-a | - |  |
| Yck1 | - | [4] |
| Ydr391c | - |  |
| Yel073c | - |  |
| Yhi9 | - |  |
| Yhr138c | - |  |
| Ykl065w-a | - |  |
| Ykl069w | - |  |
| Ykl091c |  |  |
| Yml131w | - |  |
| Ymr099c | - |  |
| Zrc1 | - |  |

**References**

1. Reggiori F, Shintani T, Nair U, et al. Atg9 cycles between mitochondria and the pre-autophagosomal structure in yeasts. Autophagy. 2005 Jul;1(2):101-9.

2. He C, Baba M, Cao Y, et al. Self-interaction is critical for Atg9 transport and function at the phagophore assembly site during autophagy. Mol Biol Cell. 2008 Dec;19(12):5506-16.

3. Matoba K, Kotani T, Tsutsumi A, et al. Atg9 is a lipid scramblase that mediates autophagosomal membrane expansion. Nat Struct Mol Biol. 2020 Dec;27(12):1185-1193.

4. Graef M, Friedman JR, Graham C, et al. ER exit sites are physical and functional core autophagosome biogenesis components. Mol Biol Cell. 2013 Sep;24(18):2918-31.

5. Sawa-Makarska J, Baumann V, Coudevylle N, et al. Reconstitution of autophagosome nucleation defines Atg9 vesicles as seeds for membrane formation. Science. 2020 Sep 4;369(6508).

6. Kakuta S, Yamamoto H, Negishi L, et al. Atg9 vesicles recruit vesicle-tethering proteins Trs85 and Ypt1 to the autophagosome formation site. J Biol Chem. 2012 Dec 28;287(53):44261-9.

7. Harada K, Kotani T, Kirisako H, et al. Two distinct mechanisms target the autophagy-related E3 complex to the pre-autophagosomal structure. Elife. 2019 Feb 27;8.

8. Legakis JE, Yen W-L, Klionsky DJ. A Cycling Protein Complex Required for Selective Autophagy. Autophagy. 2007;3(5):422-432.

9. Hawkins WD, Leary KA, Andhare D, et al. Dimerization-dependent membrane tethering by Atg23 is essential for yeast autophagy. Cell Rep. 2022 Apr 19;39(3):110702.

10. Kim B, Lee Y, Choi H, et al. The trehalose-6-phosphate phosphatase Tps2 regulates ATG8 transcription and autophagy in Saccharomyces cerevisiae. Autophagy. 2021 Apr;17(4):1013-1027.

11. Suzuki K, Nakamura S, Morimoto M, et al. Proteomic profiling of autophagosome cargo in Saccharomyces cerevisiae. PLoS One. 2014;9(3):e91651.

12. Segarra VA, Sharma A, Lemmon SK. Atg27p localization is clathrin- and Ent3p/5p-dependent. MicroPubl Biol. 2021 Published 2021 Mar 29.(2578-9430 (Electronic)).

13. Lu K, Psakhye I, Jentsch S. Autophagic clearance of polyQ proteins mediated by ubiquitin-Atg8 adaptors of the conserved CUET protein family. Cell. 2014 Jul 31;158(3):549-63.

14. Lebesgue N, Megyeri M, Cristobal A, et al. Combining Deep Sequencing, Proteomics, Phosphoproteomics, and Functional Screens To Discover Novel Regulators of Sphingolipid Homeostasis. J Proteome Res. 2017 Feb 3;16(2):571-582.

15. Bontron S, Jaquenoud M, Vaga S, et al. Yeast endosulfines control entry into quiescence and chronological life span by inhibiting protein phosphatase 2A. Cell Rep. 2013 Jan 31;3(1):16-22.

16. Yeasmin AM, Waliullah TM, Kondo A, et al. Orchestrated Action of PP2A Antagonizes Atg13 Phosphorylation and Promotes Autophagy after the Inactivation of TORC1. PLoS One. 2016;11(12):e0166636.

17. Sarkar S, Dalgaard JZ, Millar JB, et al. The Rim15-endosulfine-PP2ACdc55 signalling module regulates entry into gametogenesis and quiescence via distinct mechanisms in budding yeast. PLoS Genet. 2014 Jun;10(6):e1004456.

18. Miller JP, Lo RS, Ben-Hur A, et al. Large-scale identification of yeast integral membrane protein interactions. Proceedings of the National Academy of Sciences. 2005;102(34):12123-12128.

19. Schu P, Wolf DH. The proteinase yscA-inhibitor, IA3, gene. Studies of cytoplasmic proteinase inhibitor deficiency on yeast physiology. FEBS Lett. 1991 May 20;283(1):78-84.

20. Takeshige K, Baba M, Tsuboi S, et al. Autophagy in yeast demonstrated with proteinase-deficient mutants and conditions for its induction. Journal of Cell Biology. 1992;119(2):301-311.

21. Budovskaya YV, Stephan JS, Reggiori F, et al. The Ras/cAMP-dependent protein kinase signaling pathway regulates an early step of the autophagy process in Saccharomyces cerevisiae. J Biol Chem. 2004 May 14;279(20):20663-71.

22. Waite KA, De-La Mota-Peynado A, Vontz G, et al. Starvation Induces Proteasome Autophagy with Different Pathways for Core and Regulatory Particles. J Biol Chem. 2016 Feb 12;291(7):3239-53.

23. Marshall RS, McLoughlin F, Vierstra RD. Autophagic Turnover of Inactive 26S Proteasomes in Yeast Is Directed by the Ubiquitin Receptor Cue5 and the Hsp42 Chaperone. Cell Rep. 2016 Aug 9;16(6):1717-1732.

24. Marshall RS, Vierstra RD. A trio of ubiquitin ligases sequentially drives ubiquitylation and autophagic degradation of dysfunctional yeast proteasomes. Cell Rep. 2022 Mar 15;38(11):110535.

25. Buchan JR, Kolaitis RM, Taylor JP, et al. Eukaryotic stress granules are cleared by autophagy and Cdc48/VCP function. Cell. 2013 Jun 20;153(7):1461-74.

26. Krick R, Bremer S, Welter E, et al. Cdc48/p97 and Shp1/p47 regulate autophagosome biogenesis in concert with ubiquitin-like Atg8. J Cell Biol. 2010 Sep 20;190(6):965-73.

27. Nakamura N, Matsuura A, Wada Y, et al. Acidification of Vacuoles Is Required for Autophagic Degradation in the Yeast, *Saccharomyces cerevisiae*. The Journal of Biochemistry. 1997;121(2):338-344.

28. Marquardt L, Taylor M, Kramer F, et al. Vacuole fragmentation depends on a novel Atg18-containing retromer-complex. Autophagy. 2023 Jan;19(1):278-295.

29. Duran JM, Anjard C, Stefan C, et al. Unconventional secretion of Acb1 is mediated by autophagosomes. J Cell Biol. 2010 Feb 22;188(4):527-36.

30. Montegut L, Joseph A, Chen H, et al. DBI/ACBP is a targetable autophagy checkpoint involved in aging and cardiovascular disease. Autophagy. 2023 Jul;19(7):2166-2169.

31. Bockler S, Westermann B. Mitochondrial ER contacts are crucial for mitophagy in yeast. Dev Cell. 2014 Feb 24;28(4):450-8.

32. Eiyama A, Okamoto K. Protein N-terminal Acetylation by the NatA Complex Is Critical for Selective Mitochondrial Degradation. J Biol Chem. 2015 Oct 9;290(41):25034-44.

33. Monastyrska I, He C, Geng J, et al. Arp2 Links Autophagic Machinery with the Actin Cytoskeleton. Molecular Biology of the Cell. 2008;19(5):1962-1975.

34. Liu D, Mari M, Li X, et al. ER-phagy requires the assembly of actin at sites of contact between the cortical ER and endocytic pits. Proc Natl Acad Sci U S A. 2022 Feb 8;119(6).

35. Suzuki SW, Yamamoto H, Oikawa Y, et al. Atg13 HORMA domain recruits Atg9 vesicles during autophagosome formation. Proc Natl Acad Sci U S A. 2015 Mar 17;112(11):3350-5.

36. Rao Y, Perna MG, Hofmann B, et al. The Atg1-kinase complex tethers Atg9-vesicles to initiate autophagy. Nat Commun. 2016 Jan 12;7:10338.

37. Papinski D, Schuschnig M, Reiter W, et al. Early steps in autophagy depend on direct phosphorylation of Atg9 by the Atg1 kinase. Mol Cell. 2014 Feb 6;53(3):471-83.

38. Ishihara N, Hamasaki M, Yokota S, et al. Autophagosome requires specific early Sec proteins for its formation and NSF/SNARE for vacuolar fusion. Mol Biol Cell. 2001 Nov;12(11):3690-702.

39. Sekito T, Kawamata T, Ichikawa R, et al. Atg17 recruits Atg9 to organize the pre-autophagosomal structure. Genes Cells. 2009 May;14(5):525-38.

40. Zhou F, Wu Z, Zhao M, et al. Rab5-dependent autophagosome closure by ESCRT. J Cell Biol. 2019 Jun 3;218(6):1908-1927.

41. Liu X, Mao K, Yu AYH, et al. The Atg17-Atg31-Atg29 Complex Coordinates with Atg11 to Recruit the Vam7 SNARE and Mediate Autophagosome-Vacuole Fusion. Curr Biol. 2016 Jan 25;26(2):150-160.

42. Barve G, Sridhar S, Aher A, et al. Septins are involved at the early stages of macroautophagy in S. cerevisiae. J Cell Sci. 2018 Feb 22;131(4).

43. Sharmin T, Morshed S, Ushimaru T. PP2A promotes ESCRT-0 complex formation on vacuolar membranes and microautophagy induction after TORC1 inactivation. Biochem Biophys Res Commun. 2020 Apr 9;524(3):614-620.

44. Hu G, McQuiston T, Bernard A, et al. A conserved mechanism of TOR-dependent RCK-mediated mRNA degradation regulates autophagy. Nat Cell Biol. 2015 Jul;17(7):930-942.

45. Gatica D, Hu G, Liu X, et al. The Pat1-Lsm Complex Stabilizes ATG mRNA during Nitrogen Starvation-Induced Autophagy. Mol Cell. 2019 Jan 17;73(2):314-324 e4.

46. Tsuji T, Fujimoto M, Tatematsu T, et al. Niemann-Pick type C proteins promote microautophagy by expanding raft-like membrane domains in the yeast vacuole. Elife. 2017 Jun 7;6.

47. Yu F, Imamura Y, Ueno M, et al. The yeast chromatin remodeler Rsc1-RSC complex is required for transcriptional activation of autophagy-related genes and inhibition of the TORC1 pathway in response to nitrogen starvation. Biochem Biophys Res Commun. 2015 Sep 4;464(4):1248-1253.

48. Zhou F, Zou S, Chen Y, et al. A Rab5 GTPase module is important for autophagosome closure. PLoS Genet. 2017 Sep;13(9):e1007020.

49. Bas L, Papinski D, Licheva M, et al. Reconstitution reveals Ykt6 as the autophagosomal SNARE in autophagosome-vacuole fusion. J Cell Biol. 2018 Oct 1;217(10):3656-3669.

50. Kraft C, Deplazes A, Sohrmann M, et al. Mature ribosomes are selectively degraded upon starvation by an autophagy pathway requiring the Ubp3p/Bre5p ubiquitin protease. Nat Cell Biol. 2008 May;10(5):602-10.

51. Suzuki K, Kubota Y, Sekito T, et al. Hierarchy of Atg proteins in pre-autophagosomal structure organization. Genes Cells. 2007 Feb;12(2):209-18.

52. Tan D, Cai Y, Wang J, et al. The EM structure of the TRAPPIII complex leads to the identification of a requirement for COPII vesicles on the macroautophagy pathway. Proc Natl Acad Sci U S A. 2013 Nov 26;110(48):19432-7.

53. Mari M, Griffith J, Rieter E, et al. An Atg9-containing compartment that functions in the early steps of autophagosome biogenesis. J Cell Biol. 2010 Sep 20;190(6):1005-22.

54. Nair U, Jotwani A, Geng J, et al. SNARE proteins are required for macroautophagy. Cell. 2011 Jul 22;146(2):290-302.

55. Zou S, Sun D, Liang Y. The Roles of the SNARE Protein Sed5 in Autophagy in Saccharomyces cerevisiae. Mol Cells. 2017 Sep 30;40(9):643-654.

56. Morshed S, Tasnin MN, Ushimaru T. ESCRT machinery plays a role in microautophagy in yeast. BMC Mol Cell Biol. 2020 Oct 7;21(1):70.

57. Stephan JS, Yeh Y-Y, Ramachandran V, et al. The Tor and PKA signaling pathways independently target the Atg1/Atg13 protein kinase complex to control autophagy. Proceedings of the National Academy of Sciences. 2009;106(40):17049-17054.

58. Yorimitsu T, Zaman S, Broach JR, et al. Protein kinase A and Sch9 cooperatively regulate induction of autophagy in Saccharomyces cerevisiae. Mol Biol Cell. 2007 Oct;18(10):4180-9.

59. Perez-Perez ME, Zaffagnini M, Marchand CH, et al. The yeast autophagy protease Atg4 is regulated by thioredoxin. Autophagy. 2014;10(11):1953-64.

60. Kihara A, Noda T, Ishihara N, et al. Two distinct Vps34 phosphatidylinositol 3-kinase complexes function in autophagy and carboxypeptidase Y sorting in *Saccharomyces cerevisiae*. J Cell Biol. 2001 Feb 5;152(3):519-30.
