# Supplementary material for: An APEX2-based proximity-dependent biotinylation assay with temporal specificity to study protein interactions during autophagy in the yeast Saccharomyces cerevisiae
Source: Autophagy. 2024 Jul 3;20(10):2323–37. doi: 10.1080/15548627.2024.2366749 (PMC11423678; doi:10.1080/15548627.2024.2366749)
Supplement: Supplementary figures R3.docx [file KAUP_A_2366749_SM8608.docx]

**
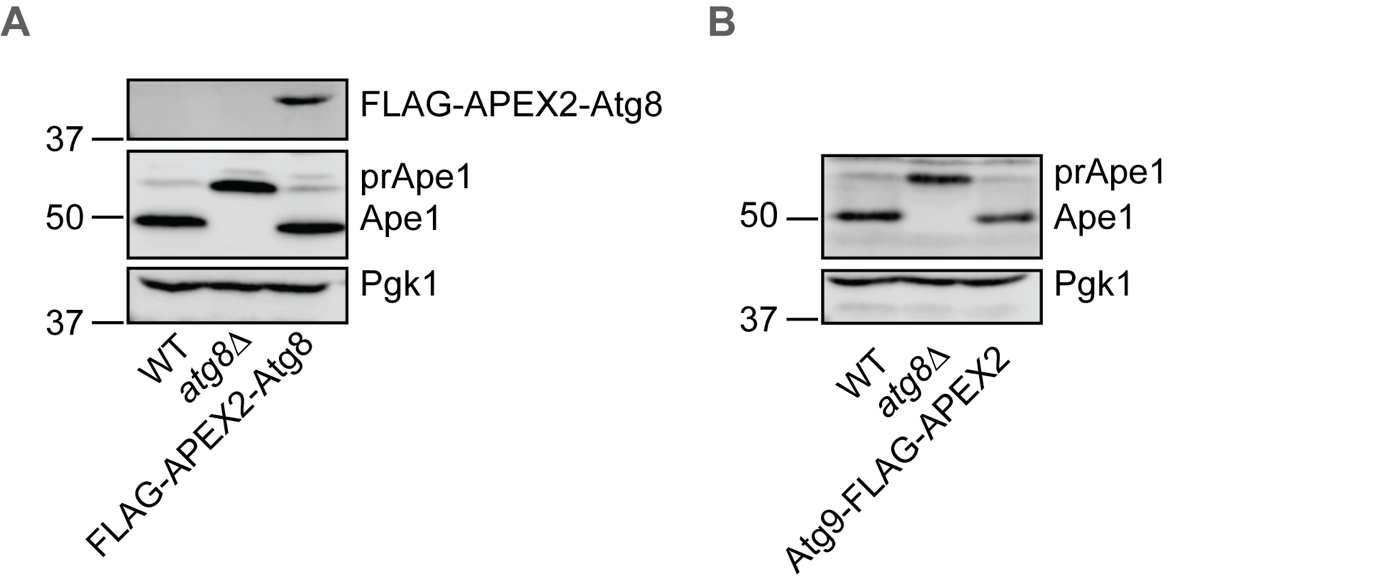
**

**Figure S1.** Analysis of the functionality of the APEX2-fusion protein used in this study. (**A**) The FLAG-APEX2-Atg8 fusion protein is functional. WT (YSBN5), *atg8∆* (YFMLY099) and FLAG-APEX2-Atg8-expressing *atg8∆* (YFMLY141) strains were grown in YPD medium overnight before collecting them to extract proteins by TCA precipitation. The expression of the fusion protein and prApe1 maturation were analyzed by western blot using anti-FLAG and anti-Ape1 antibodies, respectively. (**B**) WT (YSBN5), *atg8∆* (YFMLY099) and Atg9-FLAG-APEX2-expressing (YFMLY170) cells were grown and analyzed as in panel A. Endogenously tagged Atg9 cannot be detected with the anti-FLAG antibody. Pgk1 served as the loading control. Strains expressing Atg8, FLAG-APEX2-Atg8 or Atg9-FLAG-APEX2 processed precursor Ape1 (prApe1) into mature Ape1, while *atg8∆* cells displayed a block in prApe1 maturation.


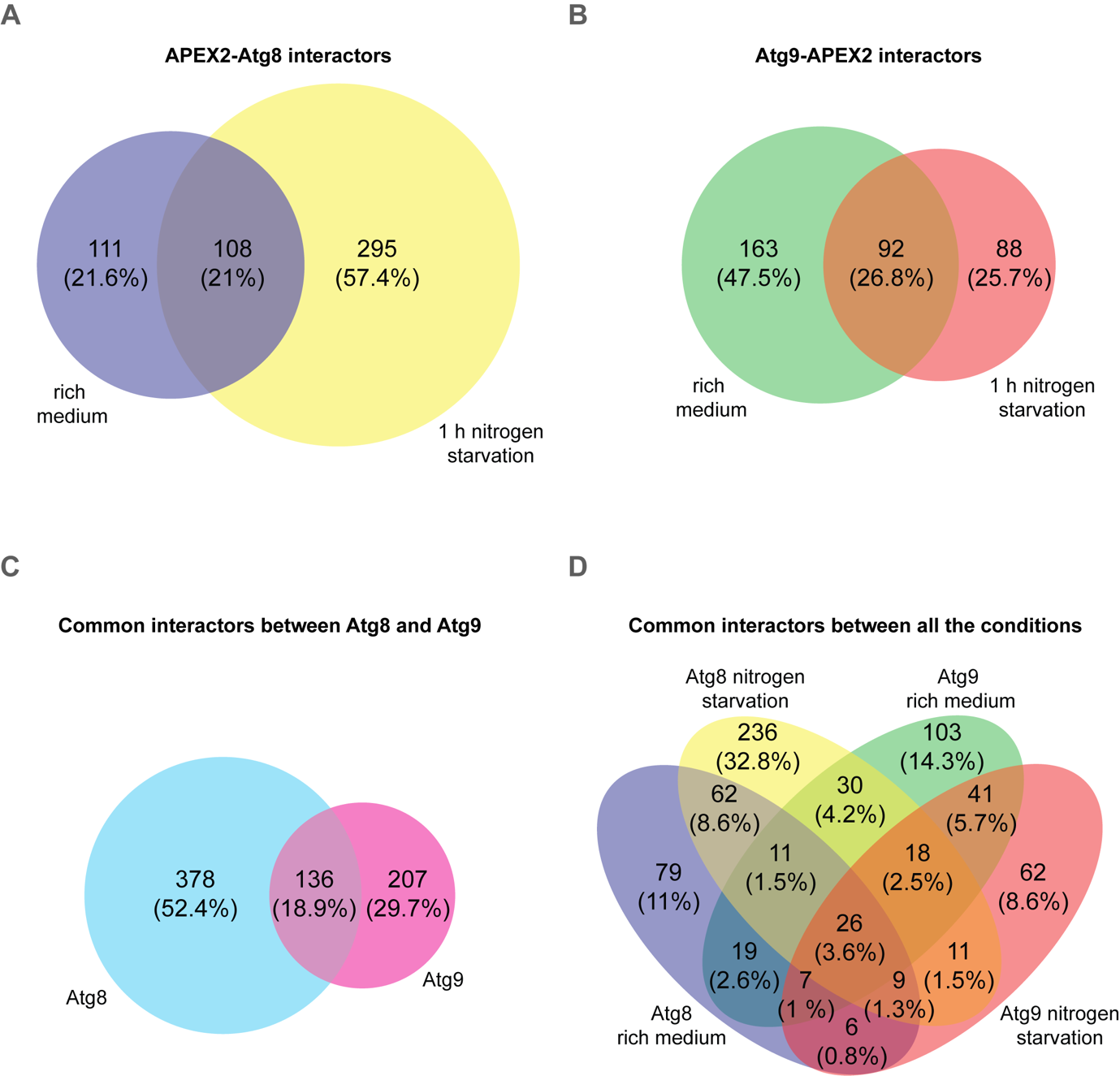


**Figure S2.** APEX2-Atg8 or Atg9-APEX2 interactors detected using the novel PL procedure. (**A**) Venn diagram showing the number and percentage of APEX2-Atg8 interactors detected either in rich medium (violet) or after 1 h of nitrogen starvation (yellow). (**B**) Venn diagram depicting the number and percentage of Atg9-APEX2 interactors detected in rich medium (green) or after 1 h of nitrogen starvation (red). (**C**) Venn diagram showing the number and percentage of common interactors between the APEX2-Atg8 (blue) and Atg9-APEX2 samples (pink). (**D**) Venn diagram showing the number and percentage of unique and common interactors between the 4 analyzed samples: APEX2-Atg8 in rich medium (violet), APEX2-Atg8 after 1 h of nitrogen starvation (yellow), Atg9-APEX2 in rich medium (green) and Atg9-APEX2 after 1 h of nitrogen starvation (red).


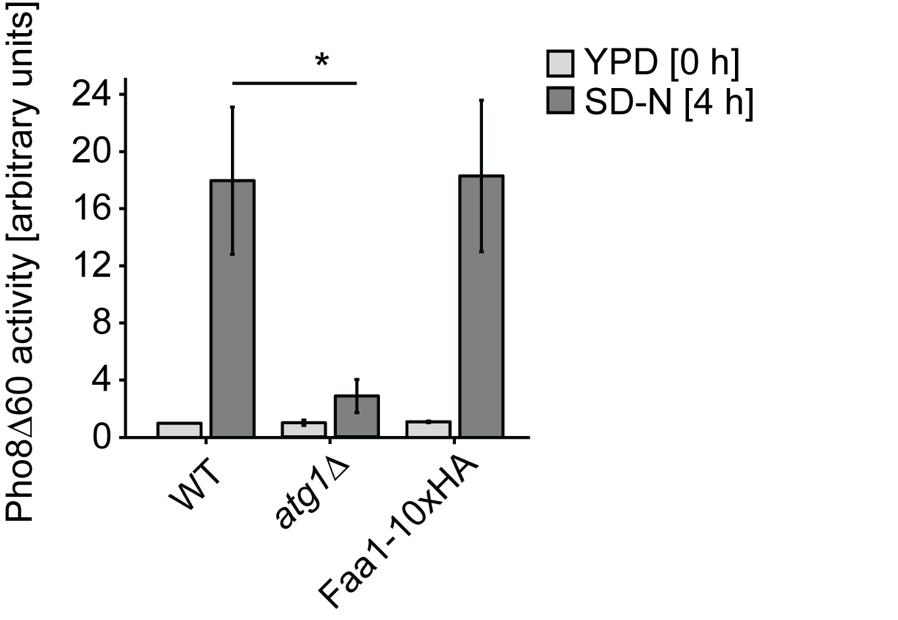


**Figure S3.** The Faa1-10xHA fusion protein is functional. The WLY176 (WT), RGY352 (*atg1∆*) and PVY048 (Faa1-10xHA) strains were grown in YPD to exponential phase before inducing autophagy by nitrogen starvation for 4 h in SD-N medium. Aliquots of cells were collected before and after nitrogen starvation, and processed for the measurement of Pho8∆60 activity as described in Materials and Methods.
